# Novel *C. elegans* models of Lewy body disease reveal pathological protein interactions and widespread miRNA dysregulation

**DOI:** 10.1007/s00018-024-05383-0

**Published:** 2024-08-30

**Authors:** Rongzhen Li, Xiaobing Huang, Linjing Shen, Tianjiao Zhang, Ning Liu, Xiangqing Hou, Garry Wong

**Affiliations:** 1 Cancer Centre, Centre of Reproduction, Development and Aging, Faculty of Health Sciences, University of Macau, E12-3005 Avenida da Universidade, Macau, 999078 China; 2 Department of Biochemistry, School of Medicine, Southern University of Science and Technology, Shenzhen, 518055 China; 3 School of Pharmacy and Food Engineering, Wuyi University, Jiangmen, 529020 China; 4 Guangzhou National Laboratory, Guangzhou International Bio Island, Guangzhou, 510005 China; 5 College of Pharmacy, Shenzhen Technology University, Shenzhen, 518118 China

**Keywords:** Amyloid β, α-Synuclein, Tau protein, TAR-DNA-binding protein 43, Parkinson’s disease

## Abstract

Lewy body diseases (LBD) comprise a group of complex neurodegenerative conditions originating from accumulation of misfolded alpha-synuclein (α-syn) in the form of Lewy bodies. LBD pathologies are characterized by α-syn deposition in association with other proteins such as Amyloid β (Aβ), Tau, and TAR-DNA-binding protein. To investigate the complex interactions of these proteins, we constructed 2 novel transgenic overexpressing (OE) *C. elegans* strains (α-syn_A53T_;Tau_pro-agg_ (OE) and α-syn_A53T_;Aβ1-42;Tau_pro-agg_ (OE)) and compared them with previously established Parkinson’s, Alzheimer’s, and Lewy Body Dementia disease models. The LBD models presented here demonstrate impairments including uncoordinated movement, egg-laying deficits, altered serotonergic and cholinergic signaling, memory and posture deficits, as well as dopaminergic neuron damage and loss. Expression levels of total and prone to aggregation α-syn protein were increased in α-syn_A53T_;Aβ_1-42_ but decreased in α-syn_A53T_;Tau_pro-agg_ animals when compared to α-syn_A53T_ animals suggesting protein interactions. These alterations were also observed at the mRNA level suggesting a pre-transcriptional mechanism. miRNA-seq revealed that *cel-miR-1018* was upregulated in LBD models α-syn_A53T_, α-syn_A53T_;Aβ_1-42_, and α-syn_A53T_;Tau_pro-agg_ compared with WT. *cel-miR-58c* was upregulated in α-syn_A53T_;Tau_pro-agg_ but downregulated in α-syn_A53T_ and α-syn_A53T_;Aβ_1-42_ compared with WT. *cel-miR-41-3p* and *cel-miR-355-5p* were significantly downregulated in 3 LBD models. Our results obtained in a model organism provide evidence of interactions between different pathological proteins and alterations in specific miRNAs that may further exacerbate or ameliorate LBD pathology.

**Supplementary Information:**

The online version contains supplementary material available at 10.1007/s00018-024-05383-0.

## Introduction

Lewy body diseases (LBD) are neurodegenerative conditions caused by accumulation of misfolded alpha-synuclein protein (α-syn) that form Lewy bodies in the central nervous system [1]. LBD can be divided into at least 3 clinicopathologic entities: Parkinson’s disease (PD), Parkinson’s disease dementia (PDD), and Dementia with Lewy bodies (DLB) based on the clinical symptoms and varying distribution of Lewy bodies. PD is the most common neurodegenerative movement disorder with central features including motor symptoms caused by neuronal loss in the substantia nigra and striatum. PD can be characterized by tremors, stiffness, bradykinesia, and neuropsychiatric symptoms including autonomic dysfunction and sleep disturbances. It is estimated that about 80% of PD patients eventually develop to PDD [2]. In PDD and DLB, Lewy bodies form in the cytoplast of cortical neurons and are characterized by cognitive impairment with cognitive fluctuation and parkinsonism. DLB and PDD are distinguishable based on the order and time interval of dementia and motor symptoms. Specifically, DLB is defined as patients with dementia before motor symptoms or dementia occurring within one year after motor symptoms. PDD is defined as patients who do not develop dementia until one year after motor symptoms appear. Furthermore, PDD with severe motor symptoms show neuronal loss in the substantia nigra which is similar to PD. However, 25% of patients with DLB do not present any motor symptoms and this subset shows preserved neurons in the substantia nigra [3].

The major component of Lewy bodies is α-syn, a 14.5 kDa natively unfolded protein whose monomers can aggregate to form soluble prefibrillar intermediate oligomers that convert to highly ordered β-sheet fibrils [4]. In patients with LBD, the ratio of α-syn tetramers-oligomers decreases [5]; subsequently, α-syn monomers bind to vesicle and lipid membranes to promote the aggregation of α-syn [6]. The Lewy body is a complex inclusion that consists of α-syn and > 90 identified components including organelles such as impaired mitochondria, lysosomes, and vesicles [7]. Subsets of LBD are related by links with several proteins like α-syn, Amyloid β (Aβ), Tau, as well as TAR-DNA-binding protein 43 (TDP-43). In the cases with dementia, 19.2% of patients displayed quadruple misfolded proteins including α-syn, Aβ, Tau and TDP-43 whereas 20.6% of patients presented with misfolded α-syn, Aβ, and Tau [8]. In the participants without clinical symptoms of dementia the following was observed: 12% presented the misfolded α-syn and Aβ; 11% of patients presented the misfolded α-syn and Tau; 3% of patients presented the misfolded α-syn and TDP-43; more than 19% of patients presented the misfolded α-syn, Aβ and Tau; and 2% of patients presented with α-syn, Tau, and TDP-43 or α-syn, Aβ, and TDP-43 [9]. Aβ plaques were observed in 50% of patients with DLB [10].Tau aggregation is observed in 25% cases of DLB [11] and the co-aggregation of α-syn and Tau were found in the same tangles [12]. Aβ can interact with α-syn and then induce the aggregation of α-syn which can lead to neurodegeneration [13]. α-Syn aggregation also induces disassembly of microtubules and causes the aggregation of Tau protein [14]. Moreover, TDP-43 frequently co-occurs with LBD. More than 30% of patients with DLB [15], 7.2% of PD patients and 19% of patients with PDD [16] presented TDP-43 pathology with co-pathology of α-syn and TDP-43 inducing a more severe neurodegeneration in PD [17]. Thus, the pathologies of PD, PDD and DLB and AD overlap [18].

*Caenorhabditis elegans* (*C. elegans*) is an invertebrate model organism utilized in the research of neurodegenerative diseases due to the high conservation of basic neuronal functions and many homologous pathologic PD genes, although not SNCA (α-syn). Previous studies by us and others have shown the ability of overexpressing transgenic animals to recapitulate some features of PD [19–22]. These transgenic models have been invaluable in understanding the molecular basis for many of the processes involved in neurodegeneration. One of the genetic effectors of neurodegenerative diseases are miRNAs, which are a type of non-coding RNA with a length of 19–24 nucleotides. MicroRNA is involved in the occurrence of Lewy body disease and have even been proposed as biomarkers or for therapy [23–29]. miRNA can affect PD and AD by binding to pathological proteins directly, interfering with the production, modification, aggregation, and degradation of pathological proteins as well [30, 31]. Nonetheless, the role of miRNAs in PDD and DLB remain largely unexplored.

In order to further understand the influence of miRNA in LBD, and to gain insight into protein interactions, we constructed 2 novel LBD models based on the pathological proteins (α-syn_A53T_;Tau_pro-agg_ (OE) and α-syn_A53T_;Aβ_1-42_; Tau_pro-agg_ (OE)) in *C. elegans*. We also utilized our 3 previously published LBD models (α-syn_A53T_ (OE), α-syn_A53T_;Aβ_1-42_ (OE), and α-syn_A53T_;TDP-43(OE)). We studied the molecular neuropathology and behavior of the different LBD models and observed interactions between α-syn_A53T_ with Aβ_1-42_ and Tau_pro-agg_. We also observed dysregulation of miRNAs in LBD models including α-syn_A53T_, α-syn_A53T_;Aβ_1-42_, and α-syn_A53T_;Tau_pro-agg_. In the present work, we constructed and analyzed LBD model animals to provide insight into the disease pathology. Our findings suggest an intimate connection between the multiple proteins involved in LBD and suggests a complex dysregulation at the miRNA level.

## Methods

### *C. elegans* strains and maintenance

All strains of *C. elegans* were maintained on nematode growth medium (NGM) plates inoculated with *Escherichia coli* (*E. coli*) OP50 at 16°C unless otherwise indicated. Upon reaching the L4 developmental stage which were marked as day 0 stage of *C. elegans* in this study, the worms were transferred to an elevated temperature of 23°C to induce the expression of the Aβ protein and to conduct subsequent experiments. All experiments were carried out under conditions ensuring adequate supply of *E. coli* OP50. Strains N2, CB1111, CB1112, CL2355, BR5270, CL6049 were obtained from the Caenorhabditis Genetics Center (CGC). UM0001, UM0010, UM0017, UM0022 were previously published as indicated in Table 1.

**Table 1:** Description of transgenic *C. elegans* strains used in this study

Strain	Genotype	Associated protein	Transgene expression	Source
N2	Wild type	–	–	CGC
CB1111	*cat-1(e1111) X*	Vesicular monoamine transporters	–	CGC
CB1112	*cat-2(e1112) II*	Putative tyrosine hydroxylase	–	CGC
UM0010	Is[*aex-3::α-syn*_*A53T*_ + *dat-1::GFP*]	Human α-synuclein_A53T_	Pan-Neuronal + dopaminergic neurons (marker)	[19]
CL2355	dvIs50[*pCL45 (snb-1::Abeta 1–42::3’ UTR(long)* + *mtl-2::GFP*]	Human Aβ peptide	Inducible pan-neuronal + intestine (marker)	CGC
BR5270	byIs161[*rab-3p::F3(delta)K280* + *myo-2p::mCherry*]	Human Pro-aggregating tau	Pan-neuronal + phyrengeal muscle (marker)	CGC[32]
CL6049	dvIs62[*snb-1p::hTDP-43/3’ long UTR* + *mtl-2p::GFP*]	Human TDP-43	Inducible pan-neuronal + intestine (marker)	CGC[36]
UM0001	Is[*snb-1p::Aβ*_*1-42*_ + *mtl-2p::GFP; rab-3p::F3(delta)K280* + *myo-2p::mCherry*]	Human Aβ peptide; human pro-aggregating tau	Inducible pan-neuronal + intestine (marker); pan-neuronal + phyrengeal muscle (marker)	[37]
UM0022	Is[*snb-1p::Aβ1-42* + *mtl-2p::GFP; aex-3::α-syn*_*A53T*_ + *dat-1::GFP*]	Human Aβ peptide; human α-synuclein_A53T_	Inducible pan-neuronal + intestine (marker); pan neuronal + dopaminergic neurons (marker)	[38]
UM0017	Is[*snb-1::TDP-43* + *mtl-2::GFP*; *aex-3::α-syn*_*A53T*_ + *dat-1::GFP*]	Human TDP-43; human α-synuclein_A53T_	Inducible pan-neuronal + intestine (marker); pan neuronal + dopaminergic neuron (marker)	[39]
UMR1	Is[*snb-1p::Aβ*_*1-42*_ + *mtl-2p::GFP; rab-3p::F3(delta)K280* + *myo-2p::mCherry;aex-3::α-syn*_*A53T*_ + *dat-1::GFP*]	Human Aβ peptide; human pro-aggregating tau; human α-synuclein_A53T_	Inducible pan-neuronal + intestine (marker); pan neuronal + phyrengeal muscle (marker); pan-neuronal + dopaminergic neurons (marker)	This study
UMR3	Is[*rab-3p::F3(delta)K280* + *myo-2p::mCherry; aex-3::α-syn*_*A53T*_ + *dat-1::GFP*]	Human pro-aggregating tau; human α-synuclein_A53T_	Pan-neuronal + intestine (marker); pan-neuronal + dopaminergic neurons (marker)	This study

### Genetic crosses

N2 males were mated with UM0010 Is[*aex-3::α-syn*_*A53T*_ + *dat-1::GFP*] hermaphrodites to produce males carrying *aex-3::α-syn(A53T)*. The male worms carrying the transgene were further mated with UM0001 Is[*snb-1p::Aβ1-42* + *mtl-2p::GFP; rab-3p::F3(delta)K280* + *myo- 2p::mCherry*] and BR5270 byIs[*rab-3p::F3(delta)K280* + *myo-2p::mCherry*] hermaphrodites. BR5270 is an integrated transgenic strain overexpressing a pro-aggregating Tau fragment. The Tau pro-aggregating fragment comprises amino acids 258–360 of the full-length tau with a K280 mutation [32]. K280 has been previously found in FTLD patients [33]. When expressed, this fragment with the mutation promotes the aggregation of wildtype and mutant Tau and thus is considered highly toxic [34, 35]. The offspring that contained all three markers including human Aβ peptide; human pro-aggregating tau and human α-syn_A53T_ were allowed to self-fertilize to homozygosity and selected under fluorescence microscopy. The genotypes of target worms were then verified by single-worm PCR (Figure S1).Markers of transgenic strains are shown in Figure S2. The primers are as follows: human *α-syn*: 5′-GGTGTTCTCTATGTAGGCTCCA, 3′-TAGGCTTCAGGTTCGTAGTCTT; human Aβ peptide (APP): 5′-CCGACATGACTCAGGATATG, 3′-GCTCACGCTATGACAACA; and human Tau (MAPT): 5′-CTCCACTGAGAACCTGAAG, 3′-GCCTAATGAGCCACACTT.

### Body bend assay

The number of body bends in 30 s were observed in adult day 1 and day 5 stage animals under a dissecting microscope. The number of body bends were only counted when head oscillation of worms was over half of the worms’ body. Thirty worms were counted per experiment and each experiment was performed in triplicate.

### Thrashing assay

Worms developed to adult day 1 and day 5 stage were randomly retrieved from the M9 buffer and subjected to recuperation for a duration of 30 s, following which the body bending frequency within that period was assayed. Thirty animals were counted per experiment, and each experiment was performed in triplicate.

### Developmental stages assay

*C. elegans* were subjected to agitation in M9 buffer for a continuous 20 h period after synchronization which ensured all worms were in the same L1 stage and subsequently transferred to fresh NGM at a temperature of 20°C. The developmental phases of *C. elegans* were identified based on the progression of the worms’ ecdysis. The time of transfer to the NGM dish was designated as 0 h, and the development phases of worms were counted for 26 h with over 200 animals evaluated per interval. The counting of the worms’ developmental progression was continued every 6 h after 40 h until all the individuals had reached the adult stages.

### Locomotion assay

Worms that developed to day 1 stage were transferred to 23°C until adult stage of day 1. Approximately 30 worms were randomly selected to the center of 35 mm plates seeded with 20 μL *E. coli* OP50. After 5 min of acclimation, the number of worms that had moved away from *E. coli* OP50 were documented every 5 min at room temperature, and the assay was conducted for 1 h. Each experiment was performed in 5 replicates.

### Tracking of *C. elegans*

A single animal developed to day 1 adult stage was picked in a 13 × 13 mm^2^ circle boundary shielded with Vaseline and inoculated with *E. coli* OP50. The locomotive progress of the organism for 1 h under room temperature was monitored using a camera (Tiannuoxiang, China). The tracks and movements of the organism were then measured using Tracker software (https://physlets.org/tracker). Each experiment was conducted in triplicate with 10 worms per trial.

### Chemotaxis assay

Animals developed to day 1 adult stage were washed gently with M9 three times. Approximately 2000 animals were chosen for the training experiment, transferred to fresh NGM with 3 circles having a diameter of 14 mm, and left to train for 1 h at room temperature. For the 3 circles, the first served as the starting point, the second was administered 1 μL 1 M sodium azide and 2 μL 100% EtOH, and the last received 1 μL 1 M sodium azide and 2 μL 10% butanone. Approximately 3000 animals were starved in M9 for 1 h at room temperature. Thereafter, these animals were transferred to fresh NGM with *E. coli OP50*, as well as 2 μL 10% butanone, for 1 h at room temperature. Following this, the animals were washed 3 times gently in M9 and transferred to the fresh NGM with the aforementioned three circles and trained for 1 h at room temperature. The number of nematodes in different areas including 3 circles and other area of plates were counted to determine the learning index of animals using the formula $\frac{{\# {\text{Attractant}} - \# {\text{Control}}}}{{\# {\text{Total}}}}$.

### Basal slowing response

Animals developed to day 1 adult stage were washed twice gently in M9 and then transferred to *E. coil OP50* seeded NGM plates. Residual M9 was removed by wicking action using a paper napkin and the locomotion of worms was recorded by camera (Tiannuoxiang, China). The locomotion of animals both inside and outside the *E. coil* OP50 lawn for 30 s was counted. Each experiment was conducted in triplicate and 30 worms were counted in each experiment.

### Dopaminergic neuron impairment assay

*C. elegans* UM0010 Is[*aex-3::α-syn*_*A53T*_ + *dat-1::GFP*], UMH6 Is[*snb-1p::Aβ1-42* + *mtl-2p::GFP; aex-3::α-syn*_*A53T*_ + *dat-1::GFP*], UM0017 Is[*snb-1::TDP-43* + *mtl-2::GFP*; *aex-3::α-syn*_*A53T*_ + *dat-1::GFP*], UMR1 Is[*snb-1p::Aβ1-42* + *mtl-2p::GFP; rab-3p::F3(delta)K280* + *myo-2p::mCherry; aex-3::α- syn(A53T)*], and UMR3 Is[*rab-3p::F3(delta)K280* + *myo-2p::mCherry; aex-3::α-syn*_*A53T*_ + *dat-1::GFP*] developed to day 5 stage were picked onto an agarose pad and anesthetized with 15 mM NaN_3_. Micrographs were taken at 400× magnification with a Zeiss confocal microscope LSM710. The images were analyzed using ZEN imaging software (Zeiss, 8.1.0.484). Each experiment consisted of at least 30 animals and was performed in 3 independent replicates.

### Viability assay

Hermaphrodite animals at adult stage day 2 were transferred from plates into 96-well plates with two animals added to each well. One-hundred μL of 5 mg/mL serotonin, or 10 mg/mL levamisole, or M9 buffer was pipetted to each well. The number of eggs in each well was counted using a stereomicroscope (Nikon, TLBD4.1) after a treatment period of 1 h. Forty-eight worms were assessed per experiment, and each experiment was conducted in triplicate.

### Worm bagging assay

Worms bagging refers to the phenomenon wherein nematodes are unable to deposit their eggs externally, which results in the hatching of larvae within the hermaphrodite body. Animals developed to day 5 adult stage under 23°C and 20°C were observed under a fluorescence microscope (Nikon, SMZ18), and the hermaphrodite worms whose eggs hatched internally due to dystocia were recorded. Each trial was undertaken in triplicate with 100 animals examined per trial.

### Lifespan assay

The worms developed to the L4 stage at 16°C were marked as day 0. Upon transfer to a temperature of 23°C for activation, the mortality of the worms was monitored daily until all animals had perished. During this period, the worms were provided with fresh NGM plates at regular intervals of two days. The entire life cycle was carried out at 23°C and with sufficient food. Any worm that failed to respond to mechanical stimulation via platinum wire was recorded as deceased. Worms that perished due to stress-induced factors, such as climbing to the edge of the NGM plates or internal deformation were censored. At least 60 worms were counted for every experiment, and at least 3 experimental replicates were performed for each experiment.

### Western Blotting

For SDS-PAGE western blotting, the worms developed to day 1 adult stage were collected and dissolved in Tris-Urea-SDS buffer (1 × TBS, 5% SDS, 8 M Urea, 50 mM dithiothreitol), which was sonicated using the Bioruptor® Plus sonication device. Then the suspension was collected and stored at  –81°C. About 20 μg of proteins were loaded onto 12% SDS–polyacrylamide gel, which was blotted onto PVDF membranes (Bio-Rad). These membranes were then blocked for 1 h at room temperature. Subsequently, primary antibodies including anti-α-syn (1:1000, Thermo/PA5-85,343) and anti-β-actin (1:1000, Santa Cruz/sc-47778) were incubated overnight at 4°C. Peroxidase AffiniPure Goat Anti-Rabbit IgG (1:5000, Jackson ImmunoResearch/111035144) and Peroxidase AffiniPure Goat Anti-Mouse IgG (1:5000, Jackson ImmunoResearch/115035146) secondary antibodies were used for detection and incubated for 1 h at room temperature. Finally, the proteins were visualized using the Clarity Western ECL Substrate kit (Bio-Rad) and imaged using ChemiDoc™ MP imaging system (Bio-Rad).

For Native-PAGE western blotting, the collected worms at day 1 adult stage were then dissolved using RAB buffer (750 mM NaCl, 100 mM MES, 1 mM EGTA, 0.5 mM MgSO4, 20 mM NaF, 1 mM PMSF, 10 mM Protease Inhibitor cocktail), and the resulting suspension was subjected to grinding through pellet pestles and stored under  –81°C. Twenty μg of proteins were loaded onto 12% Native-PAGE gel (12% Acrylamide/Bis, 0.375 M Tris with pH 8.8, 0.05% APS, and 5% TEMED for resolving; 4% Acrylamide/Bis, 126 mM Tris with pH 6.8, 0.05% APS, and 10% TEMED for stacking), which was later blotted onto PVDF membranes (Bio-Rad). Membranes with proteins were blocked 1 h at room temperature. Subsequently, primary antibodies including anti-α-syn (1:1000, Thermo/PA5-85,343) and anti-β-actin (1:1000, Santa Cruz/sc-47778) were incubated overnight at 4°C. Then, Peroxidase AffiniPure Goat Anti-Rabbit IgG (1:5000, Jackson ImmunoResearch/111035144) and Peroxidase AffiniPure Goat Anti-Mouse IgG (1:5000, Jackson ImmunoResearch/115035146) secondary antibodies were used to incubate for 1 h at room temperature. Finally, the proteins were visualized using the Clarity Western ECL Substrate kit (Bio-Rad) and imaged using ChemiDocTM MP imaging system (Bio-Rad).

### RNA-sequencing

For Small RNA-sequencing, worms at day 1 adult stage were rinsed 3 times with M9 buffer and stored in Trizol at  –81°C. RNA was precipitated using isopropanol following the sample extraction via chloroform. The RNA was washed with ethanol and dissolved in water. Ethanol-washed RNA was dried before being dissolved in nuclease-free water with ribonuclease inhibitor. NanoDrop was utilized for assessing the concentration and quality of isolated RNA. RNA samples were considered qualified with threshold OD260/OD280 ≥ 1.95 and concentration ≥ 17 ng/μL. RNA integrity was visualized with 1.2% gel electrophoresis, and RNA samples without degradation were considered acceptable. Three replicates were performed for each strain of *C. elegans*. NEBNext® Small RNA Library Prep Set for Illumina® (Biolab) was used to construct Small RNA libraries. microRNAs (~140 bp) were screened by running and cutting fragments from a 6% polyacrylamide gel and Agilent 2100 Bioanalyzer was used to check the cDNA of each sample for size, purity, and concentration. Single-end sequencing of qualified samples was performed at the HisqSE50 platform by Novogene.

For RNA-sequencing, worms at day 1 adult stage were rinsed 3 times with M9 buffer and stored in Trizol at  –81°C. NanoDrop was applied to measure the concentration and quality of isolated, and RNA samples were considered qualified with threshold OD260/OD280 ≥ 2.0 and concentration ≥ 20 ng/μL. At the same time, the integrity of RNA was examined by 1.2% gel electrophoresis, and RNA samples without degradation were considered accepted. Three replicates were performed for each strain of *C. elegans*. NEBNext Ultra RNA Library Prep Kit for Illumina (Biolab) was employed to construct RNA library. mRNA was isolated utilizing Agencourt AMPure XP Beads, and purity and concentration of the cDNA of each sample were measured through the Agilent 2100 Bioanalyzer. Finally, paired-end sequencing was performed on qualified samples utilizing the Novaseq 6000 PE150 platform by Novogene.

### Bioinformatics analysis

The raw data obtained from Novogene and internal core laboratory (Table S1) was preprocessed by trimmomatic and trim_galore and the clean data was checked by fastqc. Subsequently, all the clean data was aligned with the genome of *C. elegans* (PRJNA13758) and *Homo sapiens* (GRCh38.p14) by bowtie and hisat2. Samtools was employed to generate alignment files. Counts of every gene and miRNAs were calculated by feature count based on the annotation files of PRJNA13758 and GRCh38.p14. The differential expressed genes (DEGs) and miRNAs (DE-miRNAs) were obtained by DESeq2 and visualized by R package ggplot2 and pheatmap. Package VennDiagram was employed in R to finely visualize the Venn diagrams of DE-miRNAs. The batch effect of samples were checked by R package factoextra and FactoMineR and then were corrected by R package sva. The counts of genes and miRNAs were normalized based on the definitions of Fragments Per Kilobase of transcript per Million mapped reads (FPKM) and Count Per Million (CPM) by R. The miRTarBase database and TargetScanWorm 6.2 were utilized to identify the target genes of DE-miRNAs. The targets which were DEGs of DE-miRNAs were selected and the targets-miRNA networks were visualized by Cytoscape 3.10.0. The GO functions were conducted by DAVID database and the enrichment analysis of Kyoto Encyclopedia of Genes and Genomes (KEGG) pathway were conducted by GSEA 4.3.2. Sequencing data for this study are available from NCBI SRA database under the project accession number PRJNA1070381.

### Real-Time Quantitative Reverse Transcription PCR

Worms at day 1 adult stage were rinsed 3 times with M9 buffer and stored in Trizol at -81℃. RNA was isolated and NanoDrop was utilized to detect the concentration and assess the quality of RNA. RNA samples were considered qualified with threshold OD260/OD280 ≥ 2.0. The reverse transcription was performed by High-Capacity cDNA Reverse Transcription Kit (Thermo, 4,368,813). Power SYBR Green PCR Master Mix (Applied Biosystems, 4,309,155) and LifeTech ABI7500 Fast Realtime PCR system were employed to perform the qRT-PCR and relative expression levels of mRNA was calculated by 2^−ΔΔCt^ and normalized to the expression of gene *cdc-42*. The list of primers are in Table S2.

### Mitochondrial mass measurement

Worms developed to adult day 1 were transferred to plates with 5 μM MitoTracker™ Green (Thermo/ M7514) which was diluted by 1 mM MitoTracker™ stock solution (dissolved in dimethyl sulfoxide) and kept for 24 h at 23°C in the dark. Worms were then transferred to clean plates with no MitoTracker™ Green and allowed to crawl for 1 h. Animals were picked onto an agarose pad and anesthetized with 15 mM NaN_3_. Micrographs were taken at 630× magnification with a Zeiss confocal microscope LSM710. The images were analyzed using ZEN imaging software (Zeiss, 8.1.0.484) and Image J. Each experiment consisted of 20 animals and was performed in 3 independent replicates.

### Statistical analysis

Data were analyzed and visualized by GraphPad Prism, SPSS Statistics 22 and R and presented as mean ± SEM in this study. Differences between two groups were evaluated by Student’s t-test, while more than two groups were tested by one-way ANOVA with Tukey post-hoc. Differences were considered significant when the adjusted *P* value < 0.05. The mean lifespan of different strains was analyzed via Kaplan–Meier and finally visualized by OriginPro 8.

## Results

### LBD models show phenotypic defects in *C. elegans*

To obtain LBD models, we crossed α-syn_A53T_ with Tau_pro-agg_, and with Aβ_1-42_; Tau_pro-agg_, then screened homozygotes according to the different markers corresponding to different proteins. The marker of Aβ_1-42_ is GFP in intestines (Figure S2B). The marker of Tau_pro-agg_ is mCherry in pharyngeal muscles (Figure S2D). The marker of α-syn_A53T_ is GFP in dopaminergic neurons (Figure S2E). Finally, the genotypes of homozygotes were confirmed by single-worm PCR, and the results are shown in Figure S1. In addition, we also referenced the LBD models constructed within our laboratory, which included α-syn_A53T_[19], α-syn_A53T_;Aβ_1-42_[38]_,_ and α-syn_A53T_;TDP-43[39]. Both α-syn_A53T_;Aβ_1-42_ and α-syn_A53T_;TDP-43 contains GFP in dopaminergic neurons and intestines (Figure S2F-G). For convenience, we divided the *C. elegans* strains used in this study into three main categories: wild type (WT), control group (which included Aβ_1-42_, TDP-43, Tau_pro-agg_ and Aβ_1-42_;Tau_pro-agg_), and LBD group (which included α-syn_A53T_, α-syn_A53T_;Aβ_1-42_, α-syn_A53T_;TDP-43, α-syn_A53T_;Tau_pro-agg_, α-syn_A53T_;Aβ_1-42_;Tau_pro-agg_).

**Fig. 1:**
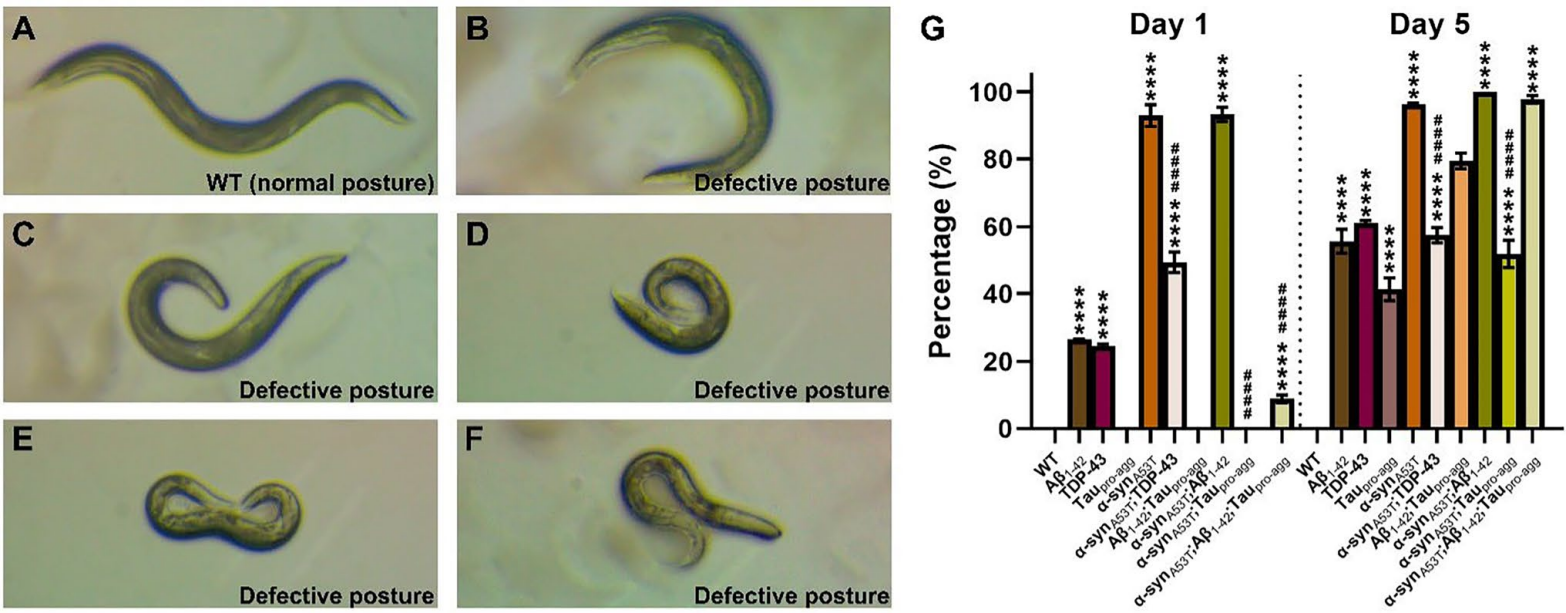
The postural observation of wild type (WT) and transgenic strains. **A** Typical normal posture of *C. elegans*. **B**–**F** Examples of defective postures of *C. elegans*. **G** The percentage of worms with defective postures. One-hundred (100) worms were counted per experiment, and each experiment was performed in triplicate (N ≥ 300 animals). Values in the panel are the average ± S.E.M. Differences between groups were evaluated by One-way ANOVA with Tukey Post-Hoc (*, comparison with WT; #, comparison with α-syn_A53T_; ****, Adjust *P* < 0.0001; ####, Adjust *P* < 0.0001). Static posture images of *C. elegans* were shown in Figure S3. Details of group comparisons are shown in Table S3

To determine whether over-expression of exogenous proteins could affect the phenotypes of worms, the different ages postures of WT and all transgenic strains were observed. For WT and control group at day 1 stage, the postures of WT, strains overexpressing Tau_pro-agg_, Aβ_1-42_;Tau_pro-agg_ and α-syn_A53T_;Tau_pro-agg_ were normal (Fig. 1A). Worms that overexpressed Aβ_1-42_ and TDP-43 exhibited nontypical postures (Fig. 1B–C). Regarding the LBD group, α-syn_A53T_ and α-syn_A53T_;Aβ_1-42_ demonstrated severe uncoordinated phenotypes, including non-sinusoidal and slow movement (Fig. 1D–F). Moreover, α-syn_A53T_;TDP-43 and α-syn_A53T_;Aβ_1-42_;Tau_pro-agg_ also exhibited uncoordinated phenotypes, albeit with a lesser degree compared to α-syn_A53T_ and α-syn_A53T_;Aβ_1-42_ (Fig. 1G). Notably, all transgenic strains showed strongly defective postures compared to WT when animals were older (Fig. 1G). These results suggest that our overexpression transgenic models recapitulate the postural deficits often observed in PD and in some forms of LBD patients.

### LBD models demonstrate developmental delay in *C. elegans*

**Fig. 2:**
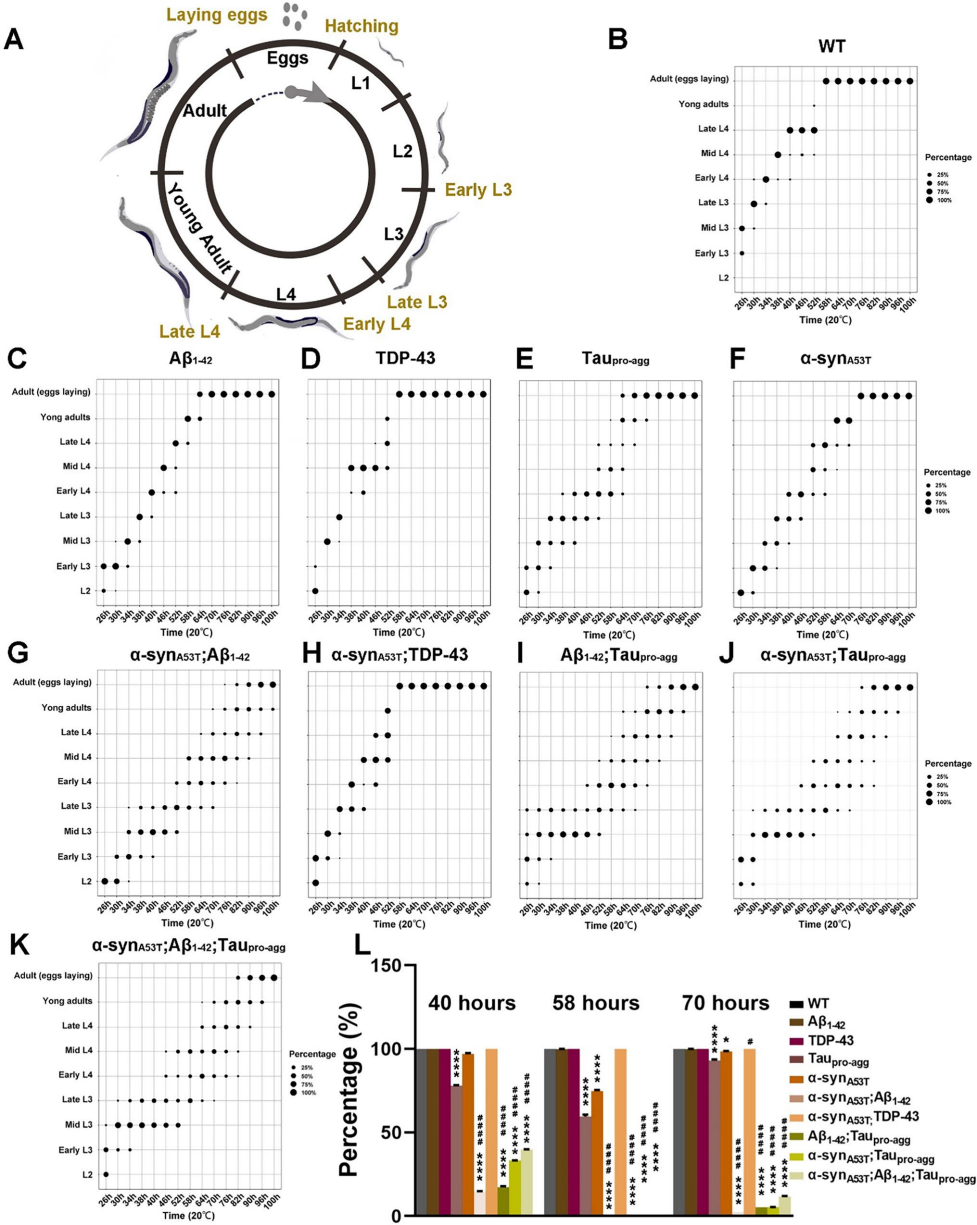
The development of wild type (WT) and transgenic strains. **A** The life cycle of *C. elegans* was drawn by Procreate and Adobe Photoshop CS6 13.0.1.1. **B**–**K** The percentage of different developmental stages of different strains

In order to determine whether the exogenous proteins including α-syn_A53T_, Aβ_1-42_, Tau_pro-agg_ and TDP-43 could affect *C. elegans* at early development, we monitored the development cycle of all transgenic strains. Five developmental stages of *C. elegans* were scored. Egg stage, L1 larva stage, L2 larva stage, L3 larva stage, L4 larva stage. The larva stage can further be divided into three sub-stages namely early, middle, and late stages (Fig. 2A). Wild type worm eggs usually enter the L3 stage between 26 to 30 h at 20°C. An additional 34 to 46 h is required for the worms to enter the L4 larva stage. At 58 h, the worms undergo metaplasia and transition to adulthood (Fig. 2A).

Control group and LBD group displayed developmental delays. The extent of developmental delays in the control group were as follows: Aβ_1-42_;Tau_pro-agg_ > Tau_pro-agg_ > Aβ_1-42_ > TDP-43 (Fig. 2C–E, I, L). Moreover, Aβ_1-42_;Tau_pro-agg_ and Tau_pro-agg_ showed strong individual difference in ontogeny. In the LBD group, all LBD models, except for α-syn_A53T_;TDP-43, experienced severe developmental delays (Fig. 2F–H,J–L). Furthermore, α-syn_A53T_;Aβ_1-42_, α-syn_A53T_;Tau_pro-agg_, and α-syn_A53T_;Aβ_1-42_; Tau_pro-agg_ displayed severe individual difference in ontogeny as well. These results indicate that the co-expression of Aβ_1-42_ and α-syn_A53T_, and the expression of Tau_pro-agg_ may adversely affect the animal’s developmental progress.

**L** The percentage of animals developed to late L3 or older at 40 h, developed to late L4 or older at 58 h, and developed to young adult or older at 70 h; different colors indicate different strains. All strains were incubated in M9 buffer to L1 stage. Time in hours was counted after bleaching and synchronization. The developmental stages of each of the 10 strains were counted after 26 h, followed by every 4 h up until 40. Thereafter, the animals’ developmental stages were counted every 6 h until all animals developed to adult stage. **A**–**L** More than 200 animals were counted at each time point, and each experiment was performed in triplicate (N ≥ 600 animals). **L** Values in the panel are the average ± S.E.M. Differences between groups were evaluated by One-way ANOVA with Tukey Post-Hoc (*comparison with WT; #, comparison with α-syn_A53T_; *Adjust *P* < 0.05; ****Adjust *P* < 0.0001; #Adjust *P* < 0.05; ####Adjust *P* < 0.0001). Details of group comparisons are shown in Table S4.

### Movement, behavioral, and lifespan deficits were observed in LBD models

**Fig. 3:**
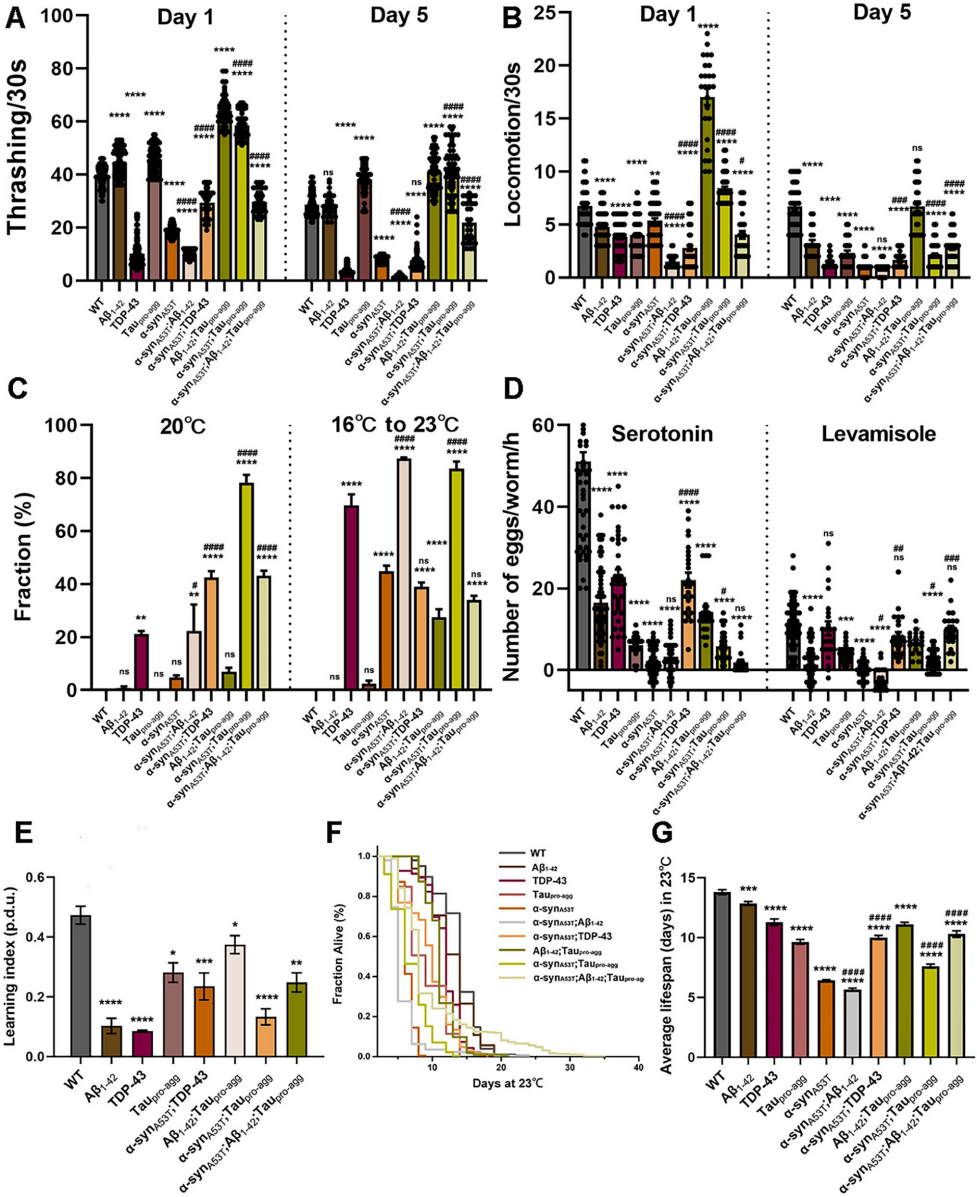
The phenotypic characteristics of wild type (WT) strain and transgenic strains. **A** Thrashing abilities of WT and transgenic strains were tested by bending in the M9 buffer. The number of body bends of animals developed to day 1 adult stage and day 5 adult stage were counted for 30 s. **B** Locomotion of WT and transgenic strains developed to day 1 adult stage and day 5 adult stage were tested by counting the body bends of animals in NGM with a lawn of live *E. coli* OP50 in 30 s. **C** Animals whose larvae developed inside their bodies were labeled as Worms’ bagging. The fractions of such animals kept under 20°C and 16°C to 23°C were counted (N ≥ 100 animals analyzed in 3 replicates). **D** The serotoninergic (5-HT) and cholinergic signaling pathways of WT strain and transgenic strains developed to day 2 adult stage were measured by counting the number of eggs of animals when exposed to M9 buffer, 5 mg/mL serotonin dissolved in M9 buffer and 10 mg/mL levamisole dissolved in M9 buffer for 1 h at room temperature (N ≥ 48 animals analyzed for each of 3 replicates). Details are shown in the Figure S4. **E** Learning index of different strains were measured by Chemotaxis assays. A higher learning index of animals indicates a stronger memory ability (N ≥ 1500 animals analyzed for each of 3 replicates). **F**–G Longevities of WT and transgenic strains. Different colors represent different strains. At least 60 worms were counted for every experiment, and at least 3 experimental replicates were performed for each experiment (N ≥ 180 animals, Mantel-Cox log-rank test; * comparison with WT, # comparison with α-syn_A53T_, ***, *P* < 0.001; ****, *P* < 0.0001; ####, *P* < 0.0001; details of groups comparison are shown in Table S10). **A**–**B** 30 worms were counted per experiment, and each experiment was performed in triplicate (N ≥ 90 animals). Movement videos of *C. elegans* developed to day 1 and day 5 stage are provided in the supplementary material. **A–E** Values in the panel are the average ± S.E.M. Differences between groups were evaluated by One-ANOVA with Tukey Post-Hoc (*, comparison with WT; #, comparison with α-syn_A53T_; ns, not significant; *, Adjust *P* < 0.05; **, Adjust *P* < 0.01; ***, Adjust *P* < 0.001; ****, Adjust *P* < 0.0001; #, Adjust *P* < 0.05; ###, Adjust *P* < 0.001; ####, Adjust *P* < 0.0001). Details of group comparisons are shown in Tables S5–S10

Thrashing and locomotion assays measure the movement ability of *C. elegans* that may recapitulate some features of the motor symptoms of LBD. The thrashing assay, where animals are placed in a liquid droplet of buffer and body bends across the body axis are counted determines ability to move freely in liquid and does not require any fine motor skills. The locomotion assay, where animals are placed on a solid agar plate with food and the media provides resistance, also measures body bends but requires more strength and coordination. While both assays measure movement, they are complementary where thrashing measures simple gross movement and locomotion measures stronger coordinated movement. To determine whether the exogenous proteins could lead to motor impairment, thrashing and locomotion of *C. elegans* assays were conducted. In the control group, the movement capacity of TDP-43 demonstrated a marked reduction, while the movement capacities of Aβ_1-42_, Tau_pro-agg_, and Aβ_1-42_;Tau_pro-agg_ showed significant elevation in comparison to the WT. In LBD group, movement capacities of α-syn_A53T_, α-syn_A53T_; Aβ_1-42_, α-syn_A53T_; TDP-43 and α-syn_A53T_; Aβ_1-42_;Tau_pro-agg_ were significantly decreased, and the movement capacity of TDP-43 showed noteworthy increase compared with WT. Additionally, the movement capacity of α-syn_A53T_;Aβ_1-42_ was significantly weaker than α-syn_A53T_; but the capacities of α-syn_A53T_;TDP-43,α-syn_A53T_;Tau_pro-agg_, and α-syn_A53T_;Aβ_1-42_;Tau_pro-agg_ considerably increased compared with α-syn_A53T_. The motility of *C. elegans* showed significant reduction with age. The capacities of TDP-43, α-syn_A53T_, α-syn_A53T_;Aβ_1-42_, α-syn_A53T_;TDP-43, an α-syn_A53T_;Tau_pro-agg_ demonstrated significant decrease with age. Moreover, α-syn_A53T_; Aβ_1-42_ essentially lost motility at day 5 adult stage (Fig. 3A) (Table S5).

In the day1 adult stage, the activity levels of Aβ_1-42_;Tau_pro-agg_, α-syn_A53T_;Tau_pro-agg_ were higher than WT, while the activity levels of other transgenic animals were significantly lower than WT. The activity levels of α-syn_A53T_; Aβ_1-42_, α-syn_A53T_;TDP-43 and α-syn_A53T_;Aβ_1-42_; Tau_pro-agg_ were lower than the activity level of α-syn_A53T_. However, α-syn_A53T_;Tau_pro-agg_ had significantly higher activity levels than α-syn_A53T_. Although the activity of WT did not change significantly with age, the activity levels of all transgenic animals decreased significantly with age, with the activities of TDP-43, α-syn_A53T_, Aβ_1-42_;Tau_pro-agg_ and α-syn_A53T_;Tau_pro-agg_ showing significant reductions. In the adult stage day 5, the activity of Aβ_1-42_;Tau_pro-agg_ was comparable to WT, but other transgenic animals exhibited lower activity levels than WT. Specifically, TDP-43, α-syn_A53T_, and α-syn_A53T_;Aβ_1-42_ were almost incapacitated. However, α-syn_A53T_;TDP-43, α-syn_A53T_;Tau_pro-agg_, and α-syn_A53T_;Aβ_1-42_;Tau_pro-agg_ showed considerably higher activity levels compared to α-syn_A53T_ in day5 adult stage (Fig. 3B) (Table S6).

Transgenic animals exhibited deficiencies in their egg-laying. Egg-laying is a complex process that involves the regulation of the serotoninergic (5-HT) and cholinergic signaling pathways [40]. The egg laying assay is a simple and convenient, yet powerful assay to measure the status of these neuronal systems in a living animal. At normal resting stages, wildtype animals will lay few eggs. However, when stimulated by the neurotransmitter serotonin or the acetylcholine agonist levamisole, animals will respond by laying a large number of eggs if the respective neurotransmitter system is functioning well. The “worm bagging” phenotype is a worst case scenario where animals are unable to lay eggs. The fertilized eggs from the hermaphrodite remain inside the worm body, hatch, grow, and can be seen moving inside the mother’s body resembling a “bag of worms”.

To measure this phenotype, we counted worm bagging proportions in animals that developed to day 5 adult stage at 20°C and 23°C. WT, Aβ_1-42_, Tau_pro-agg_, and α-syn_A53T_ presented few worms with a bagging phenotype, with Aβ_1-42_;Tau_pro-agg_ displaying a low percentage of dystocia syndrome. Conversely, TDP-43 and LBD group (excluding α-syn_A53T_) demonstrated deficiencies in their egg-laying, among which the most severe was α-syn_A53T_;Tau_pro-agg_ (78.25%), followed by α-syn_A53T_;Aβ_1-42_;Tau_pro-agg_ (43.13%) and α-syn_A53T_;TDP-43 (42.42%), then TDP-43 (21.12%) at 20°C. TDP-43 and Aβ_1-42_;Tau_pro-agg_ in the control group and α-syn_A53T_ and α-syn_A53T_;Aβ_1-42_ in the LBD group significantly increased the worms bagging ratio with temperature elevation. At 23℃, the worms bagging ratios of α-syn_A53T_;TDP-43 and α-syn_A53T_;Aβ_1-42_;Tau_pro-agg_ were comparable with α-syn_A53T_, but α-syn_A53T_;Aβ_1-42_, and α-syn_A53T_;Tau_pro-agg_ showed stronger egg-laying deficits than α-syn_A53T_ (Fig. 3C) (Table S7).

The serotoninergic pathway response of all transgenic animals were impaired (Fig. 3D) (Tables S8–S9). Serotoninergic signaling in egg-laying of α-syn_A53T_;Aβ_1-42_;Tau_pro-agg_ was basically absent. However, in the LBD group, worms α-syn_A53T_;TDP-43 and α-syn_A53T_;Tau_pro-agg_ significantly improved serotoninergic stimulated egg-laying compared to α-syn_A53T_. In contrast, α-syn_A53T_;Aβ_1-42_ and α-syn_A53T_;Aβ_1-42_;Tau_pro-agg_ did not show any significant effect on serotonergic signaling compared to α-syn_A53T_. Notably, among all transgenic animals, strains TDP-43, α-syn_A53T_;TDP-43 and α-syn_A53T_;Aβ_1-42_;Tau_pro-agg_ were found to have healthy and responsive cholinergic signaling compared to WT, while other transgenic animals had impaired cholinergic signaling. Specifically, α-syn_A53T_;Aβ_1-42_ showed the most serious deficit in the cholinergic signaling. In the LBD group, α-syn_A53T_;TDP-43, α-syn_A53T_;Tau_pro-agg_ and α-syn_A53T_;Aβ_1-42_;Tau_pro-agg_ significantly improved the cholinergic signaling, whereas α-syn_A53T_;Aβ_1-42_ exhibited more severe impairment of the cholinergic signaling compared to α-syn_A53T_ (Fig. 3D) (Tables S8–S9).

Dementia is one of the clinical symptoms of LBD. To investigate the memory capacity of different transgenic animals, we performed chemotaxis assays on the LBD group, with the exception of α-syn_A53T_ and α-syn_A53T_;Aβ_1-42_ due to their poor motor abilities. In comparison with WT, all transgenic animals exhibited considerably impaired memory capacities (Fig. 3E) which suggested α-syn_A53T_;TDP-43, α-syn_A53T_;Tau_pro-agg_ and α-syn_A53T_;Aβ_1-42_;Tau_pro-agg_ manifested a dementia phenotype.

Compared with WT, the average lifespan of all transgenic animals decreased. In the LBD group, compared with α-syn_A53T_, the average lifespan of α-syn_A53T_;Aβ_1-42_ was reduced by 12.08%, while α-syn_A53T_;TDP-43 was increased by 34.66%, and the average lifespan of α-syn_A53T_;Tau_pro-agg_ increased by 20.78%, and α-syn_A53T_;Aβ_1-42_;Tau_pro-agg_ increased lifespan by 50.72% (Fig. 3G). Furthermore, the survival curves showed α-syn_A53T_, α-syn_A53T_;TDP-43 and α-syn_A53T_;Tau_pro-agg_ exhibited small individual differences in lifespan statistics; while α-syn_A53T_;Aβ_1-42_ and α-syn_A53T_;Aβ_1-42_;Tau_pro-agg_ showed strong individual differences. The longest lifespan of α-syn_A53T_;Aβ_1-42_ was 24 days, and the longest lifespan of α-syn_A53T_;Aβ_1-42_;Tau_pro-agg_ was 35 days (Fig. 3F).

### Dopaminergic neuron functional deficits and degeneration observed in LBD models

**Fig. 4:**
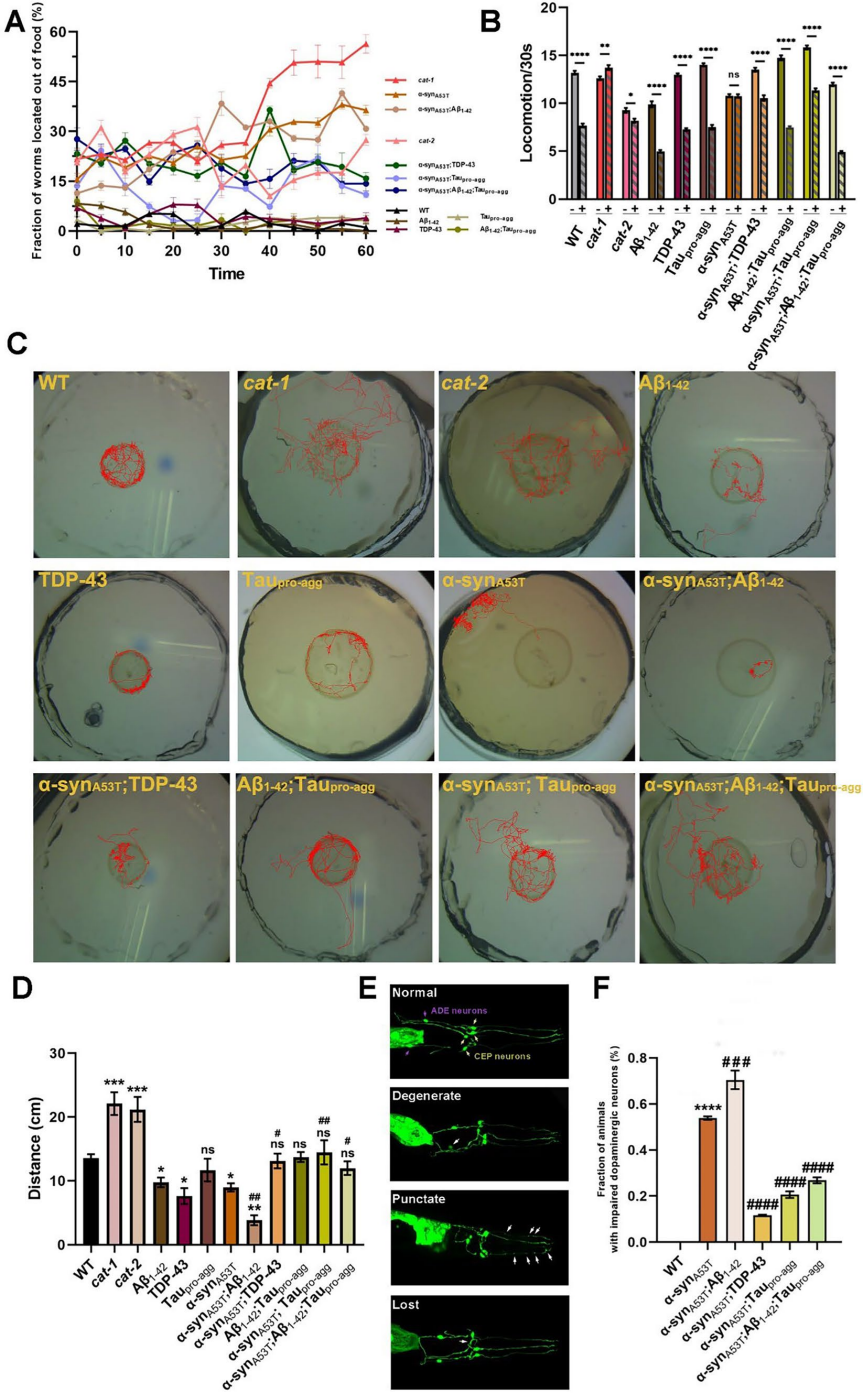
The dopaminergic neuron deficits of wild type (WT) and transgenic strains. **A** The basic locomotion based upon food sensing was measured by counting the number of worms located out of food in small NGM plates (35 mm^2^) with a lawn of live *E. coli* OP50 per 5 min for 1 h. Animals moving from the center of the food was recorded as  – 5 min. *cat-1* and *cat-2* strains were used as the positive assay controls (Tukey Post-Hoc; details of groups comparison in time points 15th, 30th, 45th, and 60th minute were shown in Figure S3 and Table S8). **B** The locomotor rates of animals which were in different conditions (*E. coli* OP50 ( +) and no *E. coli* OP50 ( – )) were counted. The decreasing locomotor rate of animals when animals were on a lawn of *E. coli* OP50 suggests functional dopaminergic neurons. To ensure the validity of this assay, *cat-1* and *cat-2* strains were set as positive controls (two sample t-test; ns, not significant; *, *P* < 0.05; **, *P* < 0.01; ****, *P* < 0.0001). **C**–**D** The movement videos of animals for 1 h in 13 mm^2^ circles were recorded by a camera installed on stereomicroscopes and then the tracks and movement distances of animals were analyzed and visualized by Tracker software. *cat-1* and *cat-2* strains were positive controls in this assay. Ten worms were counted per experiment, and each experiment was performed in triplicate (N = 30 animals). **E**–**F** The morphology observation of dopaminergic neurons in strains expressing α-syn_A53T_. **E** Models of normal and 3 types of impaired dopaminergic neurons (degenerate, punctate, lost) in *C. elegans* 2 anterior deirid neurons (ADE) and 4 cephalic neurons (CEP). The fraction of animals with impaired dopaminergic neurons (**F**) were counted by confocal microscope. At least 30 worms were counted per experiment, and each experiment was performed in triplicate (N ≥ 90 animals). **C**–**D**, **F** Values in the panel are the average ± S.E.M. Differences between groups were evaluated by One-ANOVA with Tukey Post-Hoc (*, comparison with WT; #, comparison with α-syn_A53T_; ns, not significant; *, Adjust *P* < 0.05; **, Adjust *P* < 0.01; ***, Adjust *P* < 0.001; #, Adjust *P* < 0.05; ##, Adjust *P* < 0.01; ###, Adjust *P* < 0.001; ####, Adjust *P* < 0.0001). Details of group comparisons are shown in Table S11-S14

One of the clinical symptoms in PD patients is bradykinesia, which refers to the inability to flexibly control one’s body. To assess whether animals could recapitulate these symptoms, we conducted an analysis of the capabilities of WT and transgenic animal responses to food. CAT-1 and CAT-2 are involved in dopamine metabolic process [37]. Using *cat-1* and *cat-2* null mutants as positive controls, we observed that animals in the WT and control group had a greater tendency to remain in *E. coli* OP50, while animals in LBD group had a greater tendency of staying outside the *E. coli* OP50 food lawn. The LBD group also displayed a variable ability for locomotion, with α-syn_A53T_;Aβ_1-42_ < α-syn_A53T_ < α-syn_A53T_;TDP-43 < α-syn_A53T_;Aβ_1-42_;Tau_pro-agg_ < α-syn_A53T_;Tau_pro-agg_ (Fig. 4A) (Table S11). Interestingly, however, WT, control group as well as α-syn_A53T_;Aβ_1-42_;Tau_pro-agg_ in LBD group could control their locomotion flexibly. This was evident in their capacity to adjust their speed and consequently slow down once food had been detected. Moreover, compared with WT, α-syn_A53T_, α-syn_A53T_;TDP-43, α-syn_A53T_;Tau_pro-agg_ presented weaker sensitivity of move for food. Conversely, in LBD group, α-syn_A53T_;TDP-43, α-syn_A53T_;Tau_pro-agg_ and α-syn_A53T_;Aβ_1-42_;Tau_pro-agg_ showed stronger sensitivity to move for food, compared to α-syn_A53T_, (Fig. 4B) (Table S12).

In addition, we quantified the tracks and distances of locomotion of WT and transgenic animals during a period of one hour. Our observations indicated that there was a significant decrease in locomotion distance of TDP-43, α-syn_A53T_, and α-syn_A53T_;Aβ_1-42_ in comparison to WT, especially α-syn_A53T_; Aβ_1-42_. This decrease was particularly prominent in the case of α-syn_A53T_;Aβ_1-42_. There was no significant difference observed in the locomotion distance of the other nematodes in the control and LBD group as compared to WT (Fig. 4C,D) (Table S13). Interestingly, good motility does not necessarily indicate robust body control. WT and animals in control group mainly moved in the edge of the food based on the food density, while the positive assay controls *cat-1* and *cat-2* strains are aimlessly random (Fig. 4C). In LBD groups, α-syn_A53T_, α-syn_A53T_;TDP-43, α-syn_A53T_;Tau_pro-agg_, and α-syn_A53T_;Aβ_1-42_;Tau_pro-agg_ demonstrated similar locomotion tracks to positive controls; however, α-syn_A53T_;Aβ_1-42_ was very inactive and almost never left the starting point (Fig. 4C,D).

We also observed the deficits of dopaminergic neurons in different LBD models at day 5 adult stage. Compared with α-syn_A53T_, α-syn_A53T_;Aβ_1-42_ showed more severe damage of dopaminergic neurons, while α-syn_A53T_;TDP-43, α-syn_A53T_;Tau_pro-agg_, α-syn_A53T_;Aβ_1-42_;Tau_pro-agg_ showed comparatively lesser deficiency of dopaminergic neurons (Fig. 4E–F) (Table S14). The overall impairment in food sensing based upon the locomotion assays on and off food were observed in the LBD models. These were consistent with the morphological damage observed in their dopaminergic neurons.

### α-Syn aggregation and expression are impacted by co-expression of other transgenes

**Fig. 5:**
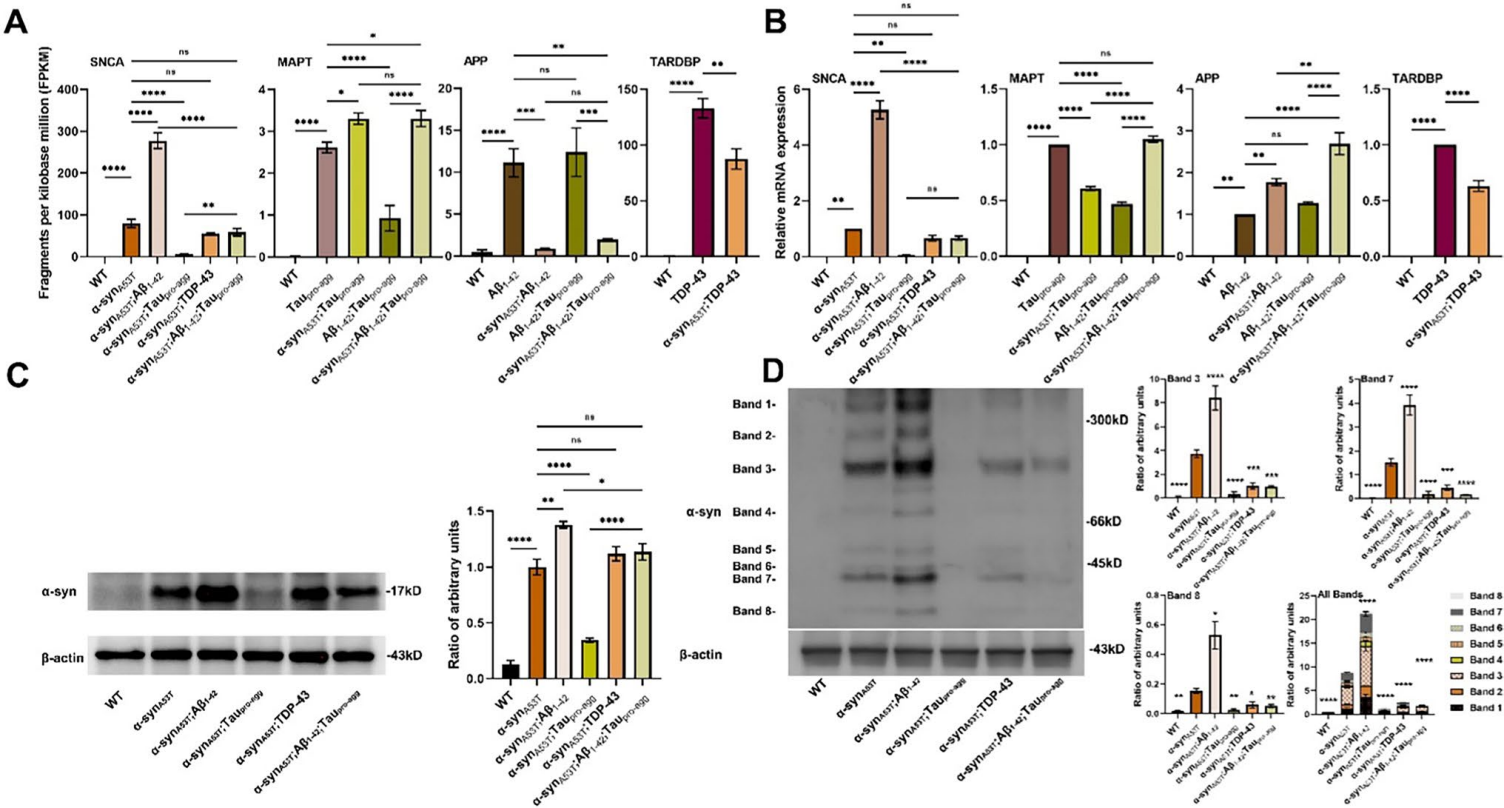
Expression level of genes SNCA, MAPT, APP, TARDBP, and protein α-syn. **A** The normalization expression levels (FPKM) of SNCA, MAPT, APP and TARDBP in WT and transgenic strains based on the RNA-seq data obtained from Novaseq 6000 PE150 platform and aligned with GRCh38.p14. **B** The relative expression levels of SNCA, MAPT, APP and TARBP based on Real-Time Quantitative Reverse Transcription PCR. **C**–**D** Twenty μg protein samples were loaded and the relative proteins amount (α-syn_A53T_/β-actin) of α-syn monomer and different sizes of α-syn oligomers were calculated. Values in the panel are the average ± S.E.M. Data were analyzed using Tukey Post-Hoc (*ns* not significant; *, Adjust *P* < 0.05; **, Adjust *P* < 0.01; ***, Adjust *P* < 0.001, ****, Adjust *P* < 0.0001). Values of group comparisons are shown in Tables S15-S18

To evaluate the impacts among α-syn_A53T_, Tau_pro-agg_, Aβ_1-42_and TDP-43 transgenes, we counted FPKM and relative expression levels of genes SNCA encoding protein α-syn, MAPT encoding protein Tau, APP encoding protein Aβ and TARDBP encoding protein TDP-43 by analyzing RNA-seq and qRT-PCR data (Fig. 5A–B) (Tables S15–S16). The expression of Aβ_1-42_ could increase the expression of SNCA, while the expression of Tau_pro-agg_ could reduce the expression of α-syn_A53T_ while TDP-43 and Aβ_1-42_;Tau_pro-agg_ had no significant effects on the expression of SNCA. The expression of α-syn_A53T_ reduced the expression of TARDBP. The agreement between RNA-seq and qRT-PCR results were good for SNCA and TARDBP but less for MAPT and APP. This could be due to the low expression levels of the later 2 proteins or differences in the technology platforms used.

To evaluate the impact of protein Aβ_1-42_, Tau_pro-agg_, TDP-43, Aβ_1-42_;Tau_pro-agg_ on the expression of protein α-syn_A53T_, we performed protein quantification on animals in LBD group. Our results demonstrated that the expression of Aβ_1-42_ could increase the expression of α-syn_A53T_, while the expression of Tau_pro-agg_ can reduce the expression of α-syn_A53T_ considerably and TDP-43 and Aβ_1-42_;Tau_pro-agg_ had no significant effects on the expression of α-syn_A53T_ (Fig. 5C) (Table S17. Furthermore, the expressions of α-syn_A53T_ gene in different LBD models were found to be consistent with the expression of α-syn_A53T_ protein (Fig. 5A–B).

There are various sizes of α-syn aggregation in the brains of PD patients. We could observe distinct sizes of α-syn_A53T_ prone to aggregation in animals with α-syn_A53T_ or α-syn_A53T_;Aβ_1-42_ expression. Compared to α-syn_A53T_, α-syn_A53T_;Aβ_1-42_ exhibited increased α-syn_A53T_, while α-syn_A53T_;TDP-43, α-syn_A53T_;Tau_pro-agg_ and α-syn_A53T_;Aβ_1-42_;Tau_pro-agg_ demonstrated less α-syn_A53T_ prone to aggregation, particularly α-syn_A53T_;Tau_pro-agg_ (Fig. 5D) (Table S18). The highest levels of α-syn prone to aggregation was observed in α-syn_A53T_;Aβ_1-42_ animals (Fig. 5D) and they had poor scores in thrashing, locomotion, and egg laying while in contrast, α-syn_A53T_;TDP-43, α-syn_A53T_;Tau_pro-agg_ and α-syn_A53T_;Aβ_1-42_;Taupro-agg had better scores (Fig. 2). This suggested a correlation in the levels of prone to aggregation α-syn for these phenotypes.

The phenotypic severity shown by our LBD models were not absolutely correlated with the total amount of α-syn_A53T_ in nematodes (Fig. 5C) but was significantly correlated with different sizes of α-syn_A53T_ prone to aggregation (Fig. 5D).

### miRNA expression levels are dysregulated in LBD models

**Fig. 6:**
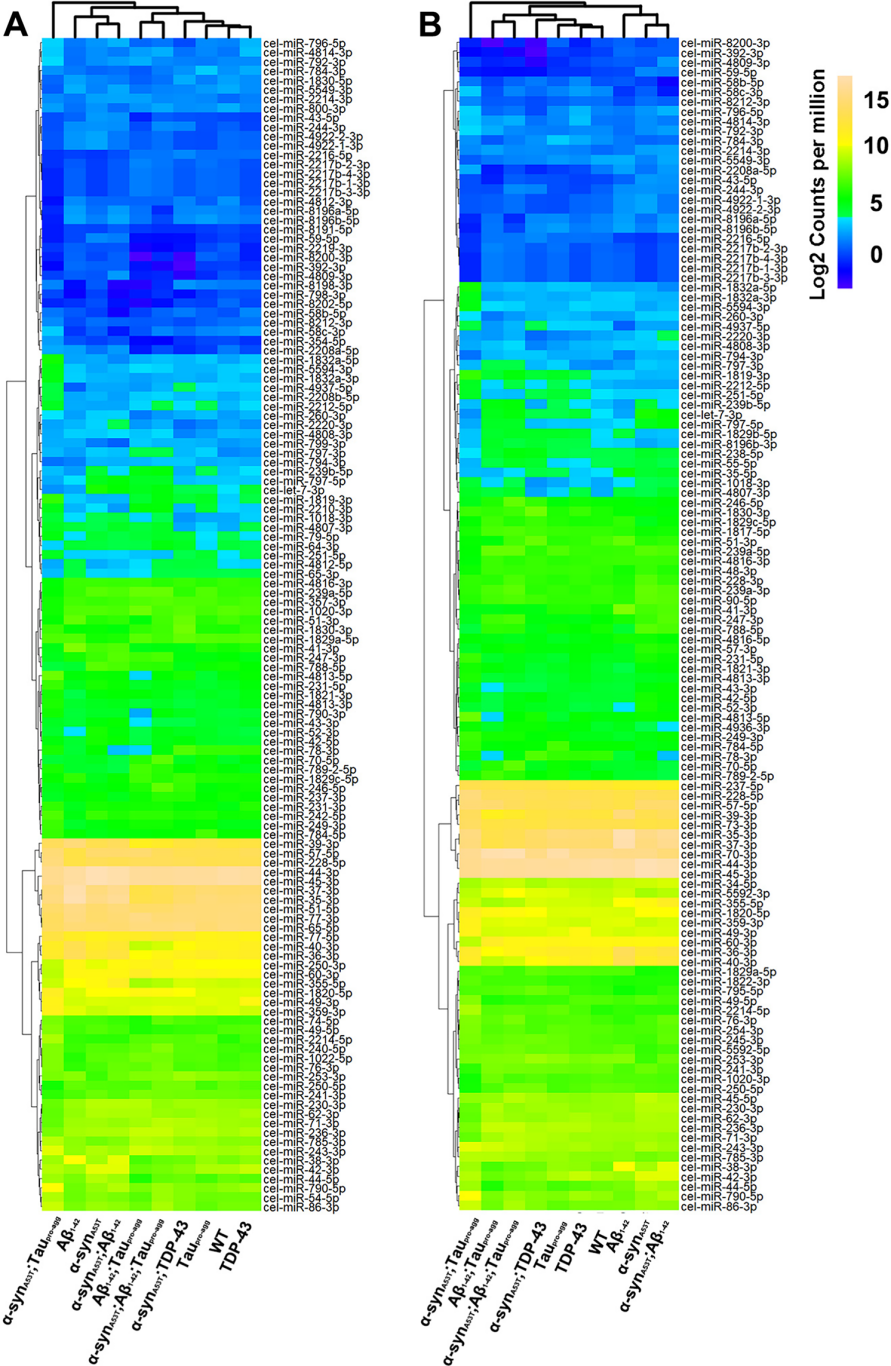
Hierarchical clustering analysis of differentially expressed miRNAs (DE-miRNAs). **A** miRNAs from transgenic strains containing α-syn_A53T_ compared with WT are listed (**B**) WT, α-syn_A53T_;TDP-43, α-syn_A53T_;Tau_pro-agg_ and α-syn_A53T_;Aβ_1-42_; Tau_pro-agg_ compared with α-syn_A53T_. Different colors indicate different expression levels (counts per million, CPM) of miRNAs. The Log2(Fold change) and adjusted *P* value of DE-miRNAs in different comparison groups are listed in Table S19 and Table S20

To visualize differential miRNA expression levels in WT and transgenic animals, the hierarchical clustering analysis of differentially expressed miRNAs (DE-miRNAs) by the sva package [41] were conducted. The |Log2 (Fold change)|> 1 and adjust *P* value ≤ 0.05 were set as thresholds to identify DE-miRNAs. Compared to WT, expression patterns of α-syn_A53T_;Aβ_1-42_;Tau_pro-agg_, and Aβ_1-42_;Tau_pro-agg_ were similar; and expression patterns of WT and TDP-43 were similar (Fig. 6A). Additionally, α-syn_A53T_ and α-syn_A53T_;Aβ_1-42_ displayed comparable expression patterns. The miRNA expression patterns of α-syn_A53T_ were different from α-syn_A53T_;TDP-43, α-syn_A53T_;Tau_pro-agg_ and α-syn_A53T_;Aβ_1-42_;Tau_pro-agg_. Compared with α-syn_A53T_, the expression patterns of DE-miRNAs in different strains were similar with the expression patterns of DE-miRNAs compared to WT (Fig. 6B). The specific DE-miRNAs and their comparisons are shown in Tables S19-S20.

**Fig. 7:**
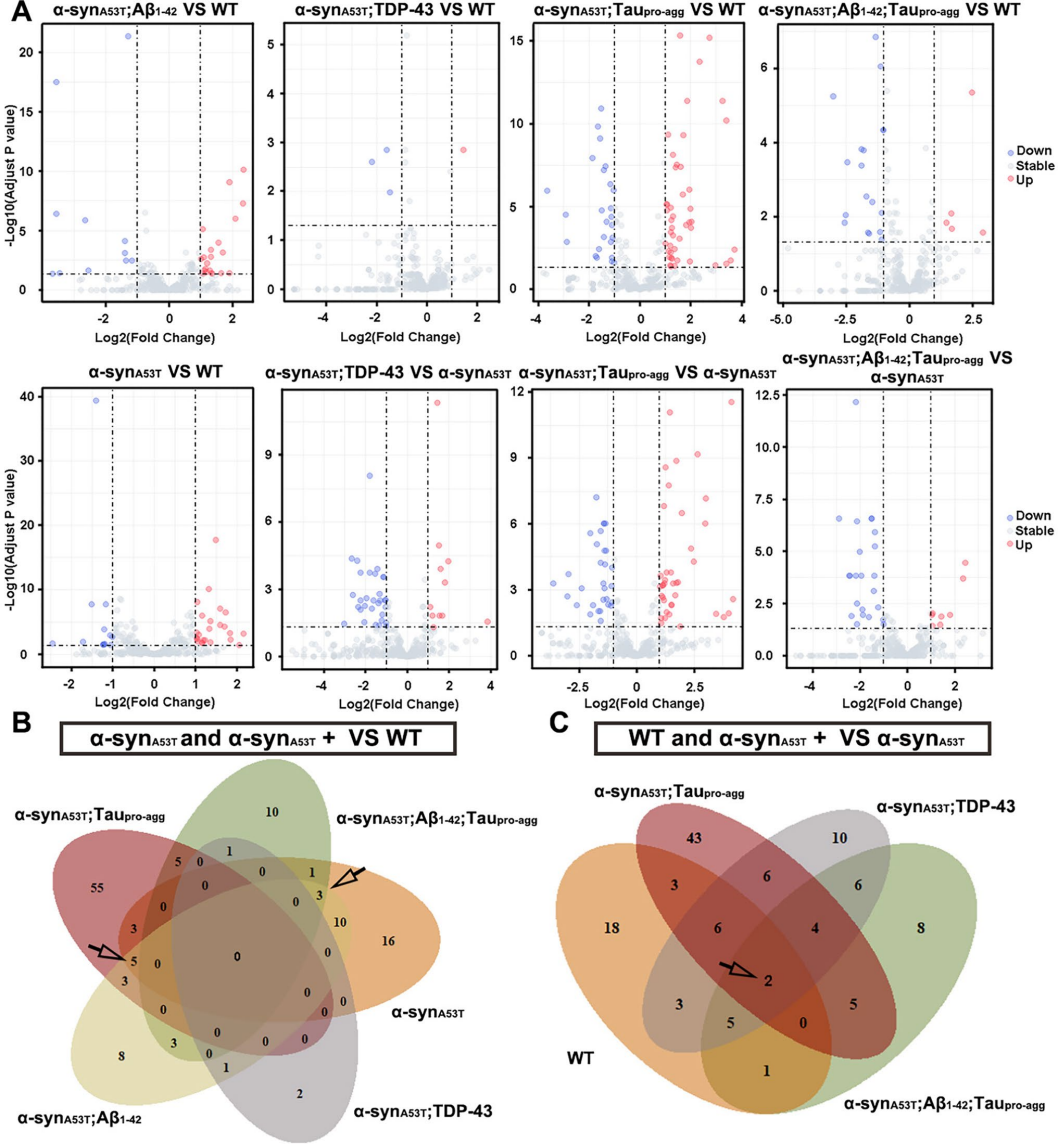
The differential expression miRNA in wild type (WT) and transgenic strains. **A** Volcano plots of differential expression miRNAs (DE-miRNAs) in transgenic strains expressed α-syn_A53T_ including α-syn_A53T_, α-syn_A53T_;Aβ_1-42_, α-syn_A53T_;TDP-43, α-syn_A53T_;Tau_pro-agg_, and α-syn_A53T_;Aβ_1-42_;Tau_pro-agg_ compared with WT and α-syn_A53T_;TDP-43, α-syn_A53T_;Tau_pro-agg_, and α-syn_A53T_;Aβ_1-42_;Tau_pro-agg_ compared with α-syn_A53T_. Log2(Fold change) > 1 and adjust *P* value < 0.05 were marked as upregulated DE-miRNAs and visualized as red dots; Log2(Fold change) < 1 and adjust *P* value < 0.05 were marked as downregulated DE-miRNAs and visualized as blue dots; miRNAs that were not DE-miRNAs were marked as stable miRNAs and visualized as grey dots. **B**–**C** Venn diagrams of different compared groups including transgenic strains that expressed α-syn_A53T_ compared with WT (**B**) and α-syn_A53T_ (**C**)

To further understand which miRNAs can affect the phenotypes of *C. elegans* in different models. We identified DE-miRNAs of α-syn_A53T_;Aβ_1-42_, α-syn_A53T_;TDP-43, α-syn_A53T_;Tau_pro-agg_, α-syn_A53T_;Aβ_1-42_; Tau_pro-agg_, and α-syn_A53T_ compared to WT and DE-miRNAs of α-syn_A53T_;Aβ_1-42_, α-syn_A53T_;TDP-43, α-syn_A53T_;Tau_pro-agg_, α-syn_A53T_;Aβ_1-42_; Tau_pro-agg_ compared to α-syn_A53T_ (Fig. 7A). However, we did not find common DE-miRNAs among α-syn_A53T_, α-syn_A53T_;Aβ_1-42_, α-syn_A53T_;TDP-43, α-syn_A53T_;Tau_pro-agg_ and α-syn_A53T_;Aβ_1-42_;Tau_pro-agg_ compared with WT. There were 5 common DE-miRNAs among α-syn_A53T_, α-syn_A53T_;Aβ_1-42_, and α-syn_A53T_;Tau_pro-agg_ compared with WT and 3 common DE-miRNAs among α-syn_A53T_, α-syn_A53T_;Aβ_1-42_, and α-syn_A53T_;Aβ_1-42_;Tau_pro-agg_ compared with WT (Fig. 7B; Table 2). Since α-syn_A53T_;TDP-43, α-syn_A53T_;Tau_pro-agg_ and α-syn_A53T_;Aβ_1-42_;Tau_pro-agg_ displayed different phenotypes and miRNA expression patterns as compared to α-syn_A53T_, we compared these 3 strains and WT with α-syn_A53T_, and obtained a total of 2 DE-miRNAs (Fig. 7C; Table 3). Taken together, these results suggested a dynamic dysregulation of miRNAs due to overexpression of the transgenes.

**Table 2:** The common differentially expressed miRNAs of the LBD group compared to WT

miRNA	α-syn_A53T_	*P*-adjust	α-syn_A53T_;Aβ_1-42_	*P*-adjust	α-syn_A53T_;TDP-43	*P*-adjust	α-syn_A53T_;Tau_pro-agg_	*P*-adjust	α-syn_A53T_;Aβ_1-42_;Tau_pro-agg_	*P*-adjust
*cel-miR-260-3p*	1.04	3.23E-03	1.20	6.06E-03	–0.17	9.50E-01	1.26	1.45E-02	–0.86	1.89E-01
*cel-miR-44-5p*	1.49	1.76E-18	1.06	7.40E-06	–0.31	5.41E-01	1.07	1.72E-05	–0.68	2.16E-02
*cel-miR-58c-3p*	–1.21	1.30E-04	–2.65	1.50E-06	0.53	9.24E-01	2.99	3.51E-02	0.94	6.85E-01
*cel-miR-4807-3p*	1.59	2.92E-05	2.33	5.39E-08	–1.08	1.28E-01	1.56	8.50E-04	1.00	8.13E-02
*cel-miR-1018-3p*	1.33	1.20E-04	1.56	1.03E-04	–0.63	5.08E-01	1.99	1.38E-05	0.97	9.12E-02
*cel-miR-42-3p*	1.09	8.87E-04	1.10	2.22E-02	–1.15	8.17E-02	0.01	9.92E-01	–1.81	1.61E-04
*cel-miR-78-3p*	–1.01	2.25E-03	–3.57	3.16E-18	0.29	8.18E-01	-0.66	2.30E-01	–1.90	1.52E-04
*cel-miR-797-3p*	1.60	8.18E-08	1.31	4.09E-04	1.87	3.86E-01	2.13	9.88E-02	2.91	2.73E-02

**Table 3:** The common DE-miRNAs of WT, α-syn_A53T_;TDP-43, α-syn_A53T_;Tau_pro-agg_, and α-syn_A53T_;Aβ_1-42_;Tau_pro-agg_ compared with α-syn_A53T_

miRNA	WT	*P*-adjust	α-syn_A53T_;TDP-43	*P*-adjust	α-syn_A53T_;Tau_pro-agg_	*P*-adjust	α-syn_A53T_;Aβ_1-42_;Tau_pro-agg_	*P*-adjust
cel-miR-41-3p	–1.05	9.99E-03	– 2.03	5.48E-03	–1.90	5.03E-03	–2.13	3.21E-03
cel-miR-355-5p	–1.16	1.12E-06	– 1.19	1.34E-02	–1.24	4.83E-03	–2.14	3.50E-07

### Hub dysregulated miRNAs were observed in LBD models

**Fig. 8:**
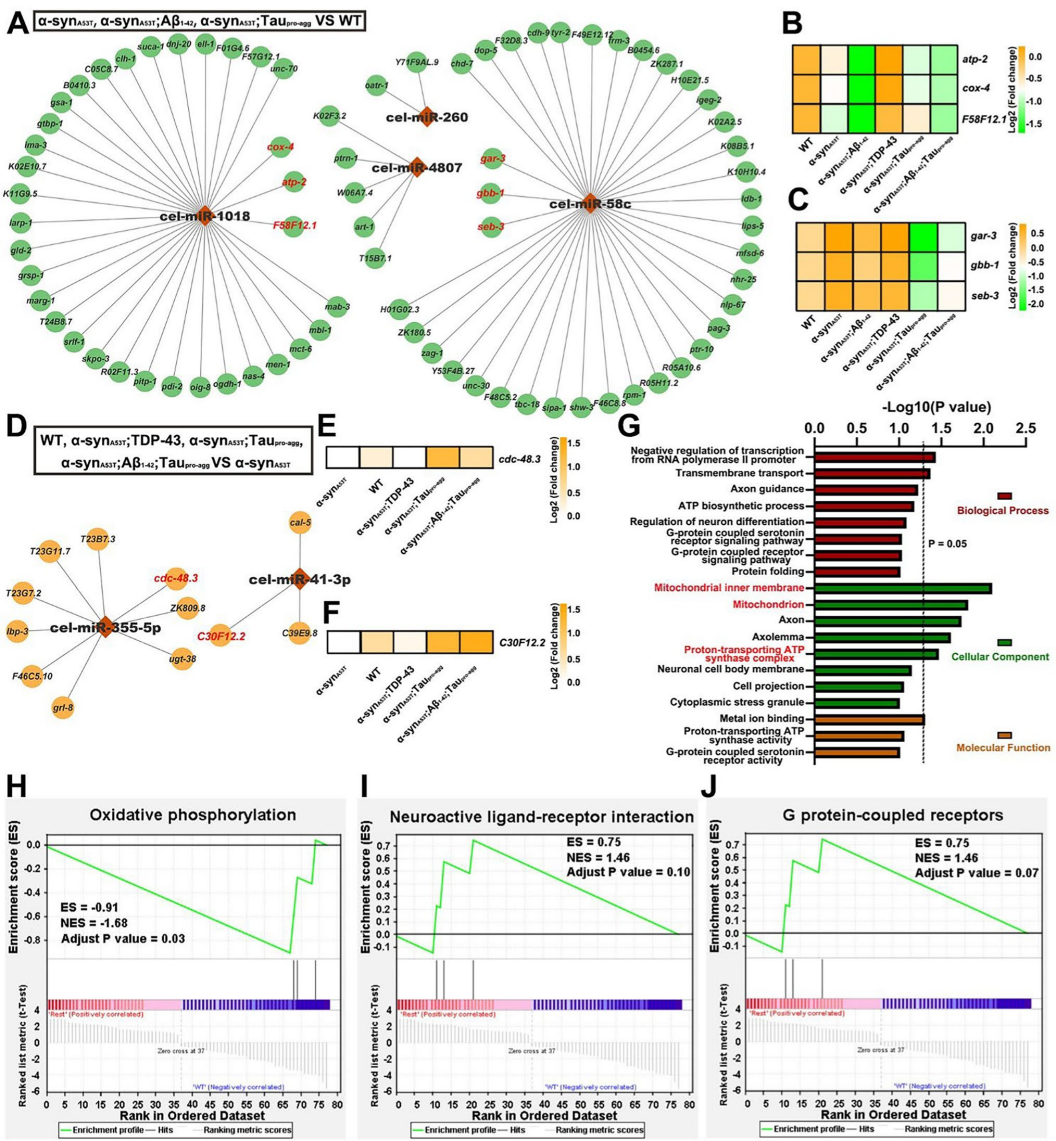
The hub-miRNAs and hub-targets and their functional analysis. **A** & **D** The network of differential expression miRNAs (DE-miRNAs) and the corresponding differential expression genes (DEGs) in transgenic strains expressed α-syn_A53T_ including α-syn_A53T_, α-syn_A53T_;Aβ_1-42_, α-syn_A53T_;Tau_pro-agg_ were compared with wild type (**A**) and wild type, α-syn_A53T_;TDP-43, α-syn_A53T_;Tau_pro-agg_, and α-syn_A53T_;Aβ_1-42_;Tau_pro-agg_ compared with α-syn_A53T_ (**D**). **B**–**C** & **E–F** The hub-targets expression patterns of hub-miRNAs including *cel-miR-1018* (B), *cel-miR-58c* (C), *cel-miR-355-5p* (E) and *cel-miR-41-3p* (F). Different colors indicated different Log2(Fold change). The GO functional analysis (GO) and Gene Set Enrichment Analysis (GSEA) based on KEGG database of *C. elegans* (H-J) of targets of hub-miRNAs which were DE-miRNAs in transgenic strains expressed α-syn_A53T_ including α-syn_A53T_, α-syn_A53T_;Aβ_1-42_, α-syn_A53T_;Tau_pro-agg_ compared with WT. The Log2(Fold change) and adjusted *P* value of DEGs showed in A and D listed in Table S21 and S22

To discern the impact of miRNA on LBD, we performed small RNA and mRNA sequencing on different LBD models and obtained DEGs and DE-miRNAs among different groups through RNA-seq and bioinformatics analysis. The targets of DE-miRNAs which showed concordant expression levels with corresponding miRNAs and were also DEGs (|Log2(Fold change)|≥ 1.5 and adjust *P* value ≤ 0.01) were identified as effective targets, and the corresponding DE-miRNAs were identified as effective DE-miRNAs. The effective DE-miRNAs and effective targets were visualized by Cytoscape. Rhombuses indicated effective DE-miRNAs and circles indicated effective DEGs. Finally, we obtained 78 effective DEGs and 4 effective DE-miRNAs in α-syn_A53T_, α-syn_A53T_;Aβ_1-42_, α-syn_A53T_;Tau_pro-agg_ compared to WT and 12 effective DEGs and 2 effective DE-miRNAs in WT, α-syn_A53T_;TDP-43, α-syn_A53T_;Tau_pro-agg_ and α-syn_A53T_;Aβ_1-42_;Tau_pro-agg_ compared to α-syn_A53T_ (Fig. 8A, D) (Table S21–S22). The functions of the 78 effective DEGs were analyzed in DAVID GO database and they impacted the mitochondria inner membrane, mitochondria, and proton-transporting ATP synthase complex (Fig. 8G) of *C. elegans*. Subsequently, the gene enrichment analysis on KEGG pathway of the 78 effective DEGs showed that oxidative phosphorylation (adjust *P* value = 0.03), neuroactive ligand-receptor interaction (adjust *P* value = 0.1), and G protein-couple receptors are significantly enriched (adjust *P* value = 0.03) (Fig. 8H–J). The FPKM of genes corresponding to oxidative phosphorylation pathway (*atp-2*, *cox-4*, F58F12.1), neuroactive ligand-receptor interaction pathway (*gar-3*, *gbb-1*, *seb-3*) and G protein-couple receptors pathway (*gar-3*, *gbb-1*, *seb-3*) in different strains were visualized by heatmap (Fig. 8B–C). The expression levels of *atp-2*, *cox-4* decreased in α-syn_A53T_, α-syn_A53T_;Aβ_1-42_, α-syn_A53T_;Tau_pro-agg_ and α-syn_A53T_;Aβ_1-42_;Tau_pro-agg_ compared to WT, but increased in α-syn_A53T_;TDP-43 compared to WT. The expression of F58F12.1 decreased in LBD group compared to WT (Fig. 8B). The expression level of *gar-3, gbb-1, seb-3* increased in LBD group except α-syn_A53T_;Tau_pro-agg_ and α-syn_A53T_;Aβ_1-42_;Tau_pro-agg_ compared to WT (Fig. 8C).

*cel-miR-1018* and *cel-miR-58c* and their targets including *atp-2, cox-4*, F58F12.1, *gar-3, gbb-1*, and *seb-3* were regarded as hub DE-miRNAs and hub-genes. In the α-syn_A53T_;TDP-43, α-syn_A53T_;Tau_pro-agg_ and α-syn_A53T_;Aβ_1-42_;Tau_pro-agg_ compared to α-syn_A53T_, *cel-miR-355-5p* and *cel-miR-41-3p* and their targets including *cdc-48.3, ZK809.8*, *utg-38*, and *C30F12.2* which are involved in the regulation of mitochondria were regarded as hub DE-miRNAs and hub DEGs. The expression levels of *cdc-48.3*, *ZK809.8*, *utg-38*, and *C30F12.2* increased in α-syn_A53T_;TDP-43, α-syn_A53T_;Tau_pro-agg_ and α-syn_A53T_;Aβ_1-42_;Tau_pro-agg_ compared to α-syn_A53T_ (Fig. 8E–F). Taken together, these results suggested that while multiple miRNAs may be dysregulated, a few select miRNAs may act as hubs to coordinate gene expression in LBD models.

### Mitochondria mass is increased in the LBD models

**Fig. 9:**
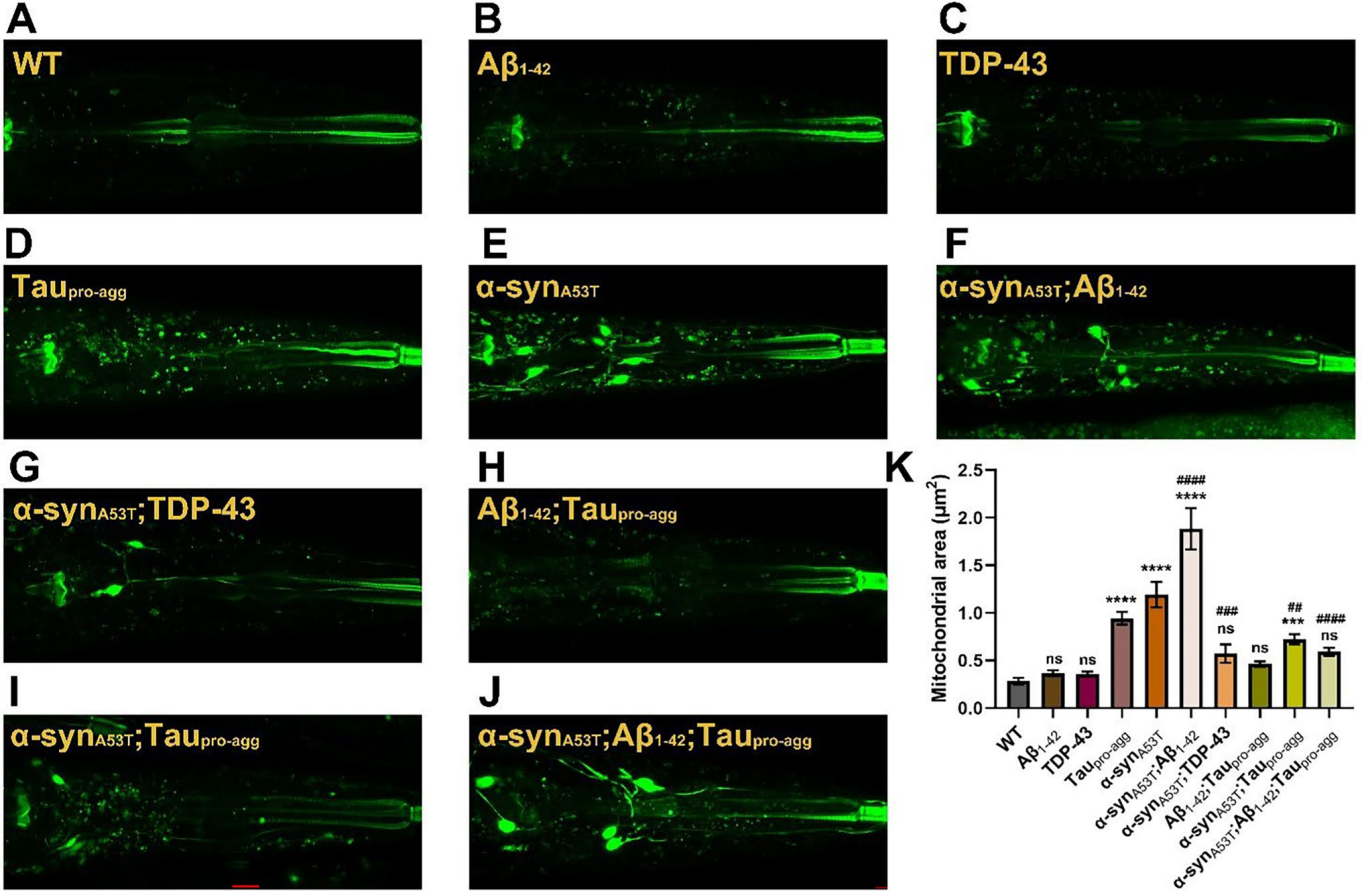
The mitochondrial mass observation of wild type (WT) and transgenic strains. (K) The mitochondrial areas of worms were counted by Image J. Twenty animals were counted per experiment, and each experiment was performed in triplicate. Values in the panel are the average ± S.E.M. Differences between groups were evaluated by One-way ANOVA with Tukey Post-Hoc (*, comparison with WT; #, comparison with α-syn_A53T_; ns, not significant; ***, Adjust *P* < 0.001; ****, Adjust *P* < 0.0001; ##, Adjust *P* < 0.01; ###, Adjust *P* < 0.001; ####, Adjust *P* < 0.0001). Details of group comparisons are shown in Table S23

Mitochondria can often be an indicator of the status of cells in stress. In order to determine the status of mitochondria we stained LBD model and single transgenic animals using mitotracker green which measures mitochondria mass (Fig. 9A–H). Using WT as baseline, we observed significant increases in the area occupied by mitochondria in single transgenic Tau_pro-agg_, α-syn_A53T_, and double transgenic α-syn_A53T_;Aβ_1-42_, with the largest increase in the latter (Fig. 9K) (Table S23). A more modest increase was observed in α-syn_A53T_;Tau_pro-agg_ animals (Fig. 9I). The sizes of mitochondria appeared larger in α-syn_A53T_ and, α-syn_A53T_;Aβ_1-42_ but not in α-syn_A53T_;TDP-43 animals (Fig. 9E–G). The effects on mitochondria mass do not appear to be additive as the double transgenic animals did not all have more mass. For example, addition of a transgene that individually has increased mass did not automatically cause an increase. Another example is the triple transgenic animal α-syn_A53T_;Aβ_1-42_;Tau_pro-agg_ which was not significantly more than WT. Thus, the mitochondria mass may depend upon the specific combination of transgenes.

## Discussion

LBD are complex neurodegenerative disorders, and their pathogenic mechanism involve the formation of Lewy bodies mainly composed of misfolded protein α-synuclein. These Lewy bodies are often found together with other proteins such as TDP-43, Tau, and Aβ [18]. To uncover the effects of co-expression of these misfolded pathogenic proteins, we constructed 2 novel LBD models: α-syn_A53T_; Tau_pro-agg_ and α-syn_A53T_;Aβ_1-42_;Tau_pro-agg_, based on α-syn_A53T_ [19], Tau_pro-agg_ [32] and Aβ_1-42_;Tau_pro-agg_ [37]. Furthermore, we have also adopted 2 other LBD models previously published, namely α-syn_A53T_;Aβ_1-42_ [38] and α-syn_A53T_;TDP-43 [39] to further investigate the pathogenesis of LBD.

In this study, LBD models including α-syn_A53T_, α-syn_A53T_;Aβ_1-42_, α-syn_A53T_;TDP-43, α-syn_A53T_;Tau_pro-agg_, and α-syn_A53T_;Aβ_1-42_;Tau_pro-agg_ exhibited phenotypes that corresponded to the clinical symptoms of LBD, such as significant stiffness and inflexibility in the body (Fig. 1), decreases in movement capacity (Fig. 3A), reductions in animal activity (Figs. 3B and 4D), lower body control ability (Fig. 4A–C), dopaminergic neuron deficits (Fig. 4F), α-syn prone to protein aggregation (Fig. 5D), and dementia (Fig. 3E). We also observed developmental delays (Fig. 2). While this is not a LBD phenotype, it indicates that effects of the transgenes may occur early in life and interpretation of our results should take this into account. Worms in our study may also be stressed by expression of multiple transgenes and we cannot rule out “transgenic sickness”. However, two lines of evidence suggest this is not the case. First, the phenotypes of the double transgenes differed depending upon the second transgene. Second, the triple transgenic animal appears to move better than the single transgenic. The models we studied here (α-syn_A53T_, α-syn_A53T_; Aβ_1-42_, α-syn_A53T_;TDP-43, α-syn_A53T_;Tau_pro-agg_, and α-syn_A53T_;Aβ_1-42_;Tau_pro-agg_) thus recapitulate some features of LBD.

In addition, since worm bagging appeared in all LBD models (Fig. 3C), we measured the health of the nematode serotonergic and cholinergic signaling pathway. Serotonergic hermaphrodite specific neurons could deliver serotonin to G protein coupled receptors, inducing nematodes to enter an egg-laying activated state [42]. The serotonergic pathways of all LBD models were damaged and α-syn_A53T_, α-syn_A53T_;Aβ_1-42_, and α-syn_A53T_;Aβ_1-42_;Tau_pro-agg_ almost had no response to serotonin (Fig. 3D), revealing impairment to the serotonergic neurons of LBD models. Cholinergic ventral C neurons are very important to help worms to lay eggs [42]. It can inhibit the egg laying of worms by releasing neurotransmitter acetylcholine. α-syn_A53T_, α-syn_A53T_;Aβ_1-42_ and α-syn_A53T_; Tau_pro-agg_ showed little response to the agonist of acetylcholine receptors named levamisole which suggests the cholinergic neurons of these 3 LBD models were impaired. We speculated that the damage of these two neurons may be one of the reasons for the formation of worms bagging in LBD models. Interestingly, the formation of worm bagging also led to shortened lifespans of nematodes. Larvae hatch in hermaphrodites, and then eating of the internal structures of mother, resulting in the death of animals. In LBD models, the α-syn_A53T_;Aβ_1-42_ individuals without worms bagging lived up to 24 days, and the α-syn_A53T_;Aβ_1-42_;Tau_pro-agg_ individuals without worms bagging lived up to 35 days (Fig. 3F).

Meanwhile, the serotoninergic system and cholinergic system are associated with impaired long-term memory function and cognitive decline. The degeneration of serotoninergic neurons and cholinergic neurons were observed in PD patients [43], which were consistent with our results (Fig. 3D) and may be one of the reasons for memory ability deficits in our LBD models (Fig. 3E).

We found that the dopaminergic neurons of LBD models suffered varying degrees of damage. The most severe LBD model was α-syn_A53T_;Aβ_1-42_, followed by α-syn_A53T_ with the second strongest dopaminergic neurons deficits, then α-syn_A53T_;TDP-43, α-syn_A53T_;Aβ_1-42_;Tau_pro-agg_ and α-syn_A53T_;Tau_pro-agg_. The dopaminergic neuron damage results were consistent with the findings of nematodes in posture, locomotor ability, activity, ability to find food, basal slowing response, and locomotion tracks. At present, it is commonly believed that the Lewy bodies formed by the misfolding of a-syn can lead to the death of dopaminergic neurons, and subsequent the clinical manifestations of LBD. The phenotypic severity shown by our LBD models were not absolutely correlated with the total amount of α-syn_A53T_ in nematodes (Fig. 5C), but was significantly correlated with different sizes of α-syn_A53T_ prone to aggregate (Fig. 5D), which was consistent with the current LBD research [44, 45]. Interestingly, the expression of human Aβ_1-42_, Tau_pro-agg_ and TDP-43 could affect the expression and α-syn_A53T_ prone to aggregate in *C. elegans*. The expression of human Aβ_1-42_ could induce the prone to aggregate α-syn_A53T_ protein and made worms sicker which was consistent with the hypothesis of interactions between Aβ protein and α-syn [46, 47]. We also found that the expression of human Tau_pro-agg_ could lower the expression and α-syn_A53T_ prone to aggregate and rescue the phenotypes of *C. elegans*. The mechanism of how Tau_pro-agg_ could lower the expression of α-syn_A53T_ was investigated. We performed outcrosses of the double transgenic animals. To our surprise, the α-syn_A53T_ animals continued to have lower expression suggesting that indeed transgene silencing occured. The nature of the silencing such as whether it is mediated by RNAi or an epigenetic phenomenon and duration in terms of number of generations remains unknown. Furthermore, the expression of Aβ_1-42_, Tau_pro-agg_ and α-syn_A53T_ together also could lower the aggregation of α-syn_A53T_ and made *C. elegans* healthier compared to α-syn_A53T_ which conflicts with current research [14]. This may be due to some unique properties of *C. elegans* not seen in humans as it does not express an α-syn ortholog or possibly that the interactions between the pathogenic proteins are more complex than previously known.

MicroRNAs are implicated in the regulation of neurodegenerative diseases. LBD group compared with WT, *cel-miR-1018, cel-miR-58c, cel-miR-260, cel-miR-4807* were involved in the regulation of LBD diseases (Fig. 8A). Computational prediction suggests that mitochondrial function is affected by the expression of target genes and thereby induces the occurrence of LBD phenotype (Fig. 8G). *cel-miR-1018* was upregulated in α-syn_A53T_, α-syn_A53T_;Aβ_1-42_, α-syn_A53T_;Tau_pro-agg_ compared with WT. *cel-miR-1018* was upregulated in dauer stage of *C. elegans*[48] which is a developmentally arrested and stress-resistant state which suggests α-syn_A53T_ may be involved in the developmental delay and induce some stress responses in *C. elegans* which were consistent with our results (Fig. 2B,F–G,J) and current research[49, 50]. The hub targets including *cox-4*, *atp-2* and *F58F12.1* of *cel-miR-1018* enriched in the oxidative phosphorylation pathway was downregulated in the α-syn_A53T_, α-syn_A53T_;Aβ_1-42_, α-syn_A53T_;Tau_pro-agg_ compared with WT (Fig. 8B–C,H) also provides further evidence that *cel-miR-1018* may affect the normal function of mitochondria by silencing *cox-4*, *atp-2* and *F58F12.1* to induce the occurrence of LBD phenotype. *cel-miR-58c* is a member of the *miR-58* family. The *miR-58* family is necessary for normal development and behavior of nematodes. They are involved in the regulation of nematode body size, movement and egg laying. The knockout of *miR-58* family causes the worms to stop development and be unable to respond to environmental pressure and enter the dauer stage [51]. Although *miR-58* is not expressed in dopaminergic neurons [52], it is involved in all developmental stages of *C. elegans *[53]. *cel-miR-58c* was downregulated in α-syn_A53T_, α-syn_A53T_;Aβ_1-42_ but upregulated in α-syn_A53T_;Tau_pro-agg_ compared with WT and the hub target *gar-3*, *gbb-1*, and *seb-3* of *cel-miR-58c* are enriched in neuroactive ligand-receptor interaction and G protein-coupled receptor, indicating that *cel-miR-58c* is involved in the regulation of nematode development and stress resistance. It may interfere with the G protein-coupled receptor pathway by regulating the expression of *gar-3*, *gbb-1*, and *seb-3*. In *C. elegans*, *cel-miR-1018* and *cel-miR-58c* may affect the phenotype of LBD by regulating the expression of corresponding targets, development, mitochondria-related pathways, neuroactive ligand-receptor interaction and G protein-coupled receptor. The *cel-miR-1018* and *cel-miR-58c* may be essential in the regulation of neurodegenerative disorder in LBD *C. elegans* models.

Although α-syn_A53T_;TDP-43,α-syn_A53T_, Tau_pro-agg_ and α-syn_A53T_;Aβ_1-42_;Tau_pro-agg_ are all LBD models, their PD phenotypes were much milder than α-syn_A53T_ and α-syn_A53T_;Aβ_1-42_. To explore the miRNAs that may be involved in regulation, 2 hub-miRNAs were identified including *cel-miR-355-5p*, *cel-miR-41-3p*. The functions of *cel-miR-355* are not fully understood. It may be involved in the regulation of innate immunity in nematodes. *miR-355* in *C. elegans* was up-regulated, and the infection rate increased because of the knockdown of *miR-355* after exposure to *Pseudomonas aeruginosa* [54]. *miR-4*, as a member of the *miR-35* family, is involved in development of *C. elegans*. The inhibition of this miRNA family can lead to poor embryonic development, stagnation or even death [51]. Therefore, we speculated *miR-355* and *miR-41* may be related to the ability of *C. elegans* to resist stress. Notably, *cdc-48.3* and *C30F12.2* were the target genes of *cel-miR-355-5p* and *cel-miR-41-3p* respectively. Both genes are members of the AAA ATPase family. They are all involved in the maintenance of mitochondrial protein proteostasis. The knockout of AAA-ATPase *C30F12.2* has been confirmed to impair oxidative stress tolerance and shorten the actual lifespan of nematodes. In addition, it may be involved in regulating the release and transport of neurotransmitters and thus affecting the health of nematodes [55]. When mitochondria are defective, they will try to repair themselves by fusing with functional mitochondria, while mitochondria that cannot be repaired will be degraded through autophagy. AAA-ATPase Cdc48 cooperates with the E3 ligase Parkin to regulate the degradation of mitofusin at the outer membrane of mitochondria, inhibiting the self-repair of defective mitochondria through fusion, thereby promoting the autophagy of defective mitochondria [56]. Therefore, we speculate that *cel-miR-355-5p* and *cel-miR-41-3p* may interfere with the normal function of mitochondria in LBD models by regulating the expression of *cdc-48.3* and C30F12.2.

One limitation of these miRNA-seq studies is that they are done in whole animals using a bulk sequencing approach. While this is convenient, single cell sequencing is becoming more common although still not routine in *C. elegans*. Future studies may use this approach and find specific miRNAs in particular neurons that act as effectors in neurodegenerative processes.

## Conclusion

Our results suggests that models including α-syn_A53T_, α-syn_A53T_;Aβ_1-42_, α-syn_A53T_;TDP-43, α-syn_A53T_; Tau_pro-agg_, and α-syn_A53T_;Aβ_1-42_; Tau_pro-agg_ demonstrate similarities to the behavior and pathogenesis of LBD such as deficits in locomotion, egg-laying, impaired serotonin and cholinergic signaling pathways, deficits in memory and body control, as well as impaired dopaminergic neurons and the formation of proteins prone to aggregate. Additionally, our study identified 4 significant miRNAs (*cel-miR-1018, cel-miR-58c, cel-miR-355-5p, and cel-miR-41-3p*) that are involved in LBD pathogenesis by regulating the expression of multiple genes and influencing the normal function of mitochondria, neuroactive ligand-receptor interaction and G protein-coupled receptor pathway. This study provides new tools to study Lewy body diseases as well as insights into the interactions of pathogenic proteins in age related neurodegenerative diseases.

### Supplementary Information

Below is the link to the electronic supplementary material.

Supplementary file1 (RAR 53279 KB)

Supplementary file2 (DOCX 4826 KB)

## Data Availability

The datasets supporting the conclusions of this article are available in the SRA database of the NCBI repository, [PRJNA1070381; https://www.ncbi.nlm.nih.gov/sra/?term=PRJNA1070381].

## Abbreviations

α-Syn: Alpha-synuclein
Aβ: Amyloid β
APP: Amyloid-beta precursor protein
BACE-1: β-Site APP cleaving enzyme
*C. elegans*: Caenorhabditis* elegans*
CGC: Caenorhaditis Genetics Center
CPM: Count Per Million
DLB: Dementia with Lewy bodies
DEGs: Differential expressed genes
DE-miRNAs: Differentially expressed miRNAs
*E. coli*: *Escherichia coli*
FPKM: Fragments per kilobase of transcript per million mapped reads
GSEA: Gene set enrichment analysis
GO: Gene ontology
GFP: Green fluorescent protein
LBD: Lewy body diseases
NGM: Nematode growth media
6-OHDA: Oxidopamine
PD: Parkinson’s disease
PDD: Parkinson’s disease dementia
RFP: PIWI-interacting RNA (piRNA); Red fluorescent proteins
TDP-43: TAR-DNA-binding protein 43 (TDP-43)
MPTP: 1-Methyl-4-phenyl-1,2,3,6-tetrahydropyridine
WT: Wild type
UTR: Untranslated region
