# Supplementary material for: Novel C. elegans models of Lewy body disease reveal pathological protein interactions and widespread miRNA dysregulation
Source: Cell Mol Life Sci. 2024 Aug 30;81(1):377. doi: 10.1007/s00018-024-05383-0 (PMC11364739; doi:10.1007/s00018-024-05383-0)
Supplement: Supplementary file 2 — Supplementary file2 (DOCX 4826 KB) [file 18_2024_5383_MOESM2_ESM.docx]

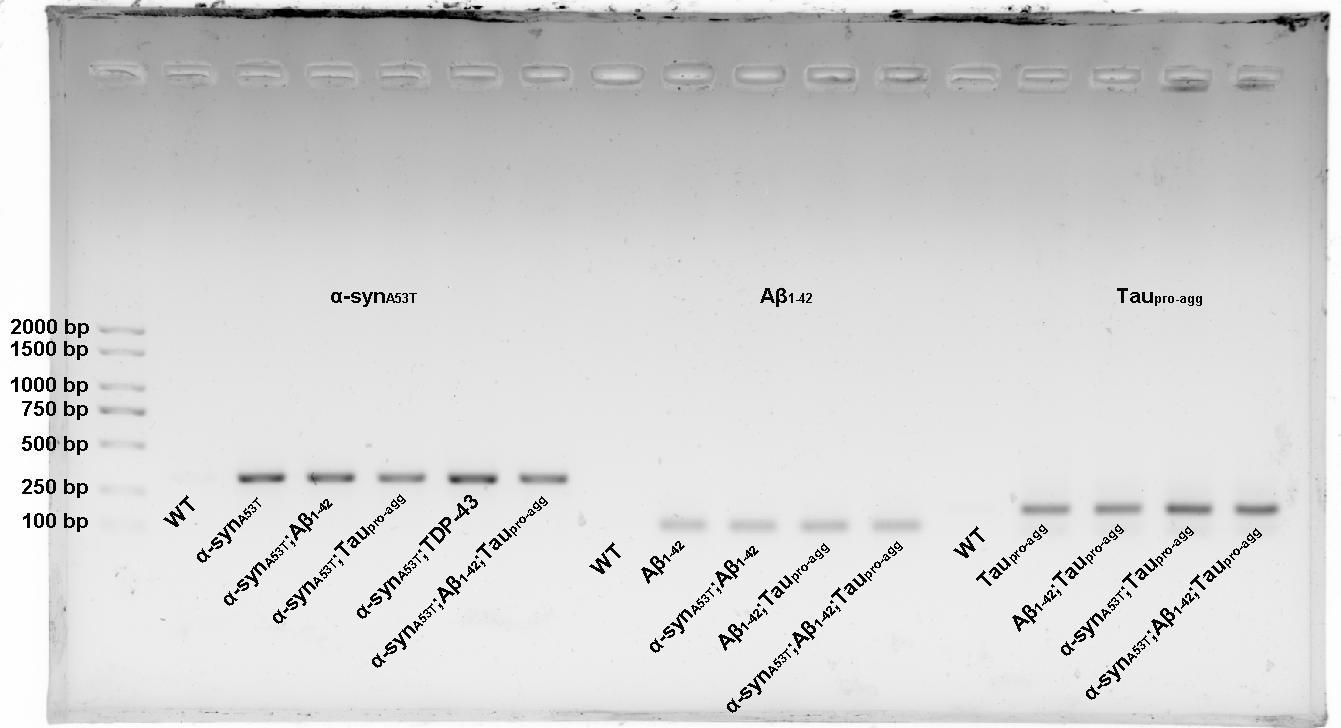


# Figure S1. The genotypes verification of wild type (WT) and transgenic *Caenorhabditis elegans* using PCR.


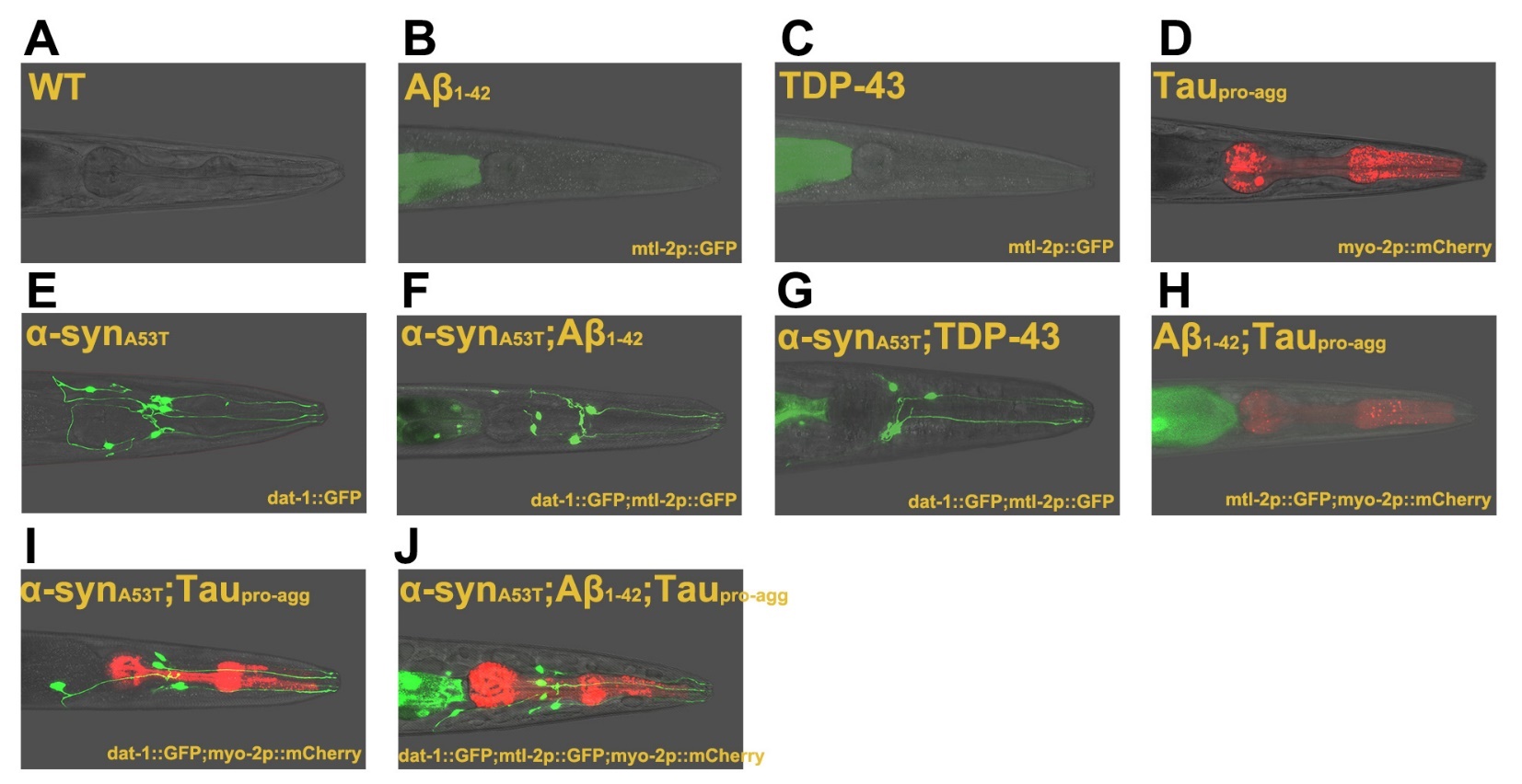


# Figure S2. Fluorescent markers of *C. elegans* models at day 1 adult stage with captured at 400× magnification by Zeiss confocal microscope LSM710. Red colors are mCherry fluorescence from red fluorescent proteins (RFP), which was expressed in pharyngeal muscles of worms. Green colors are green fluorescent proteins (GFP) expressed in dopaminergic neurons and intestines of worms.


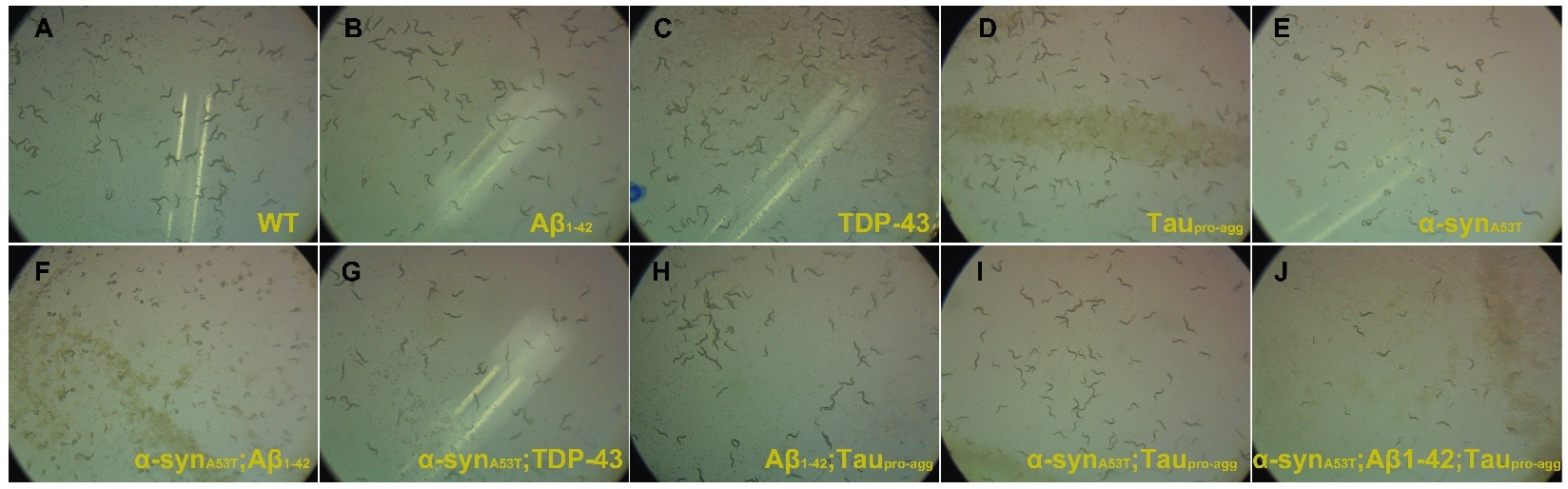


# Figure S3. The postural observation of wild type (WT) and transgenic strains which were developed at day 1 adult stage. The *C. elegans* postures of various models of Lewy body diseases were captured by transmitted light stereomicroscopy.


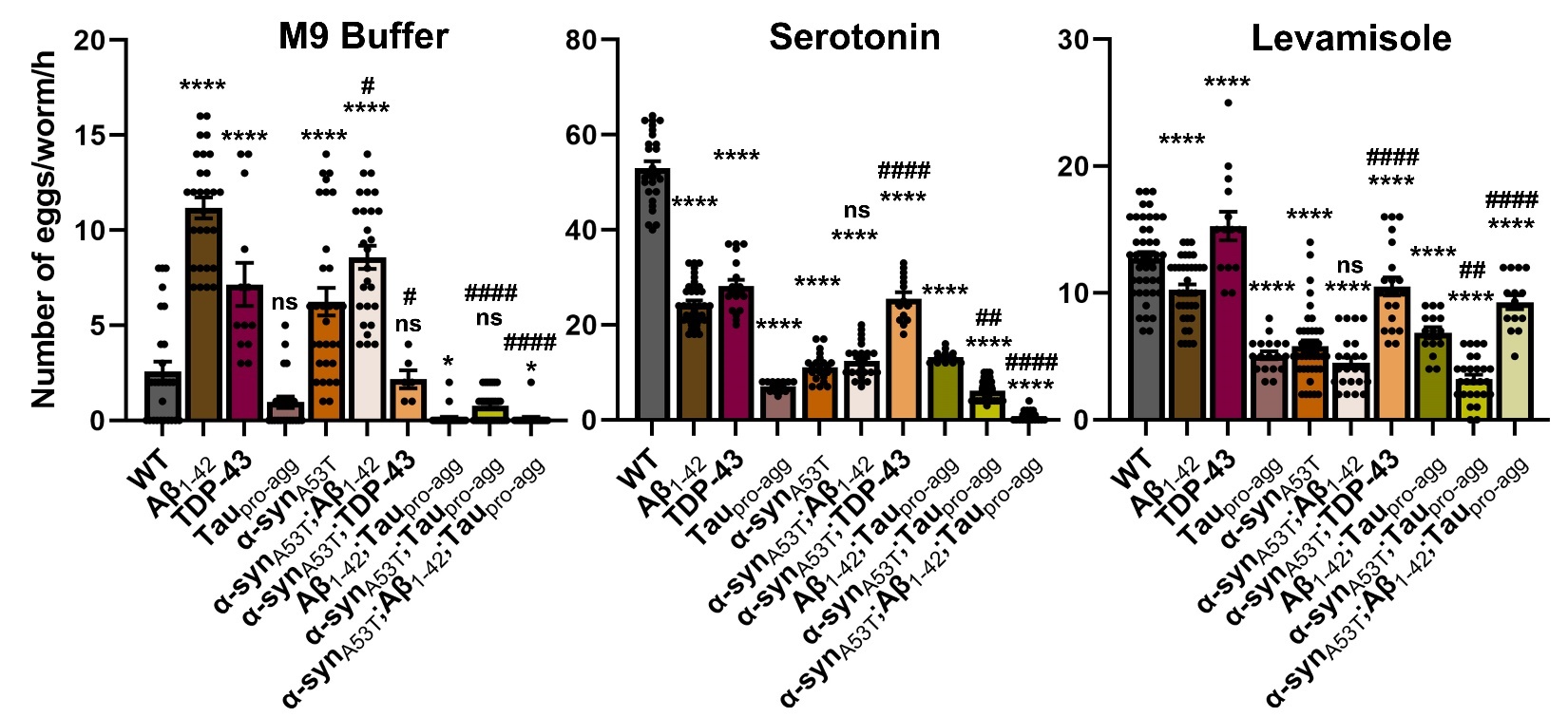


# Figure S4. The eggs number of wild type and transgenic *Caenorhabditis elegans* in 1 hour under different situations including M9 buffer, 5 mg/mL serotonin solution and 10 mg/mL levamisole solution. Values in the panel are the average (+/− S.E.M). Differences between groups were evaluated by One-ANOVA with Tukey Post-Hoc (*, comparison with WT; #, comparison with α-syn_A53T_; ns, not significant; *, Adjust *P* <0.05; ****, Adjust *P* <0.0001; #, Adjust *P* <0.05; ####, Adjust *P* <0.0001). Details of group comparisons are shown in Table S8.


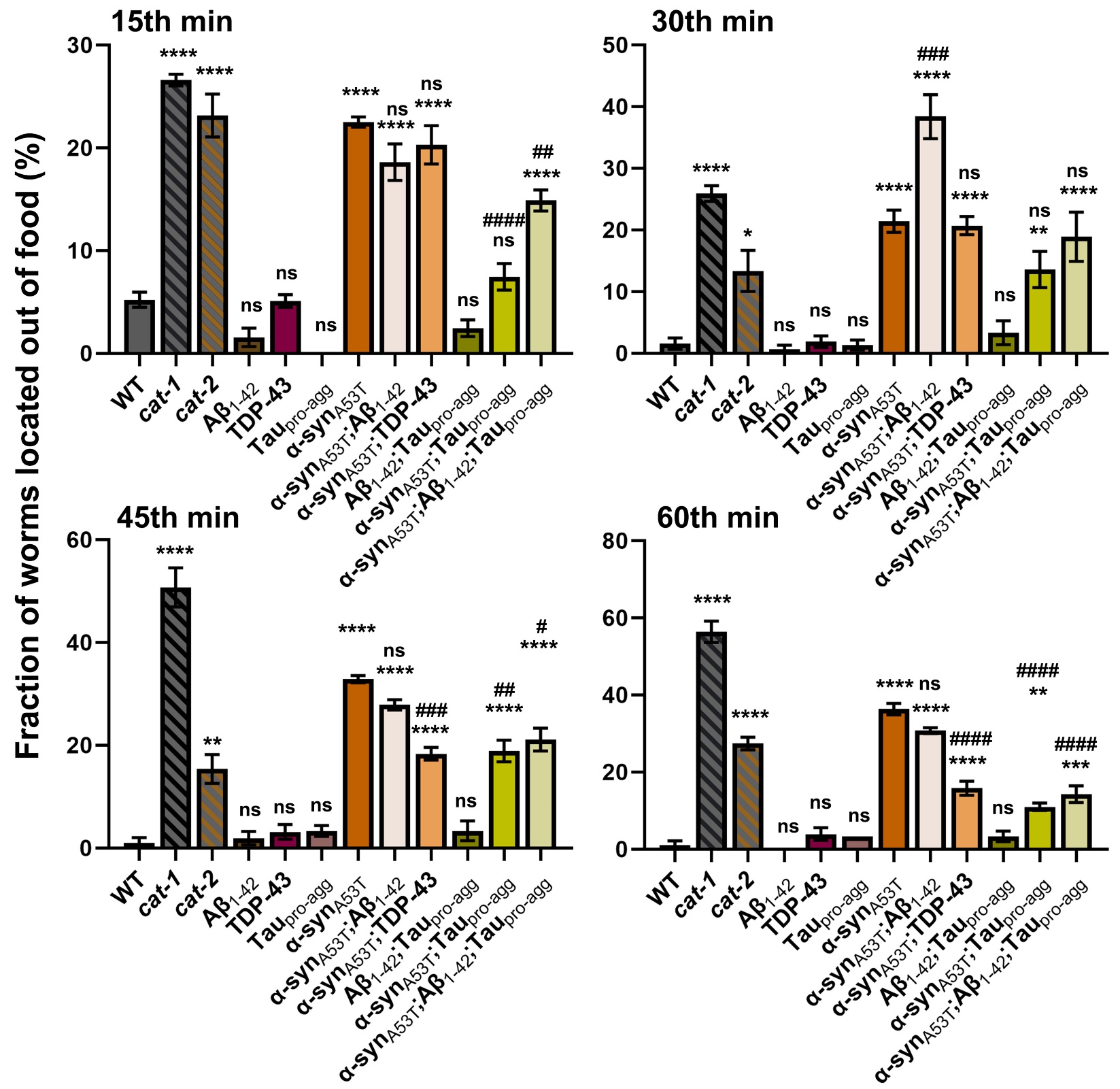


# Figure S5. The fraction of wild type and transgenic *Caenorhabditis elegans* located out of food in time points 15^th^, 30^th^, 45^th^, and 60^th^ minutes. Values in the panel are the average (+/− S.E.M). Differences between groups were evaluated by One-ANOVA with Tukey Post-Hoc (*, comparison with WT; #, comparison with α-synA53T; ns, not significant; *, Adjust *P* < 0.05; **, Adjust *P* <0.01; ***, Adjust *P* < 0.001; #, Adjust *P* <0.05; ##, Adjust *P* < 0.01; ###, Adjust *P* < 0.001; ####, Adjust *P* < 0.0001) and more details of groups comparison are shown in Table S11.


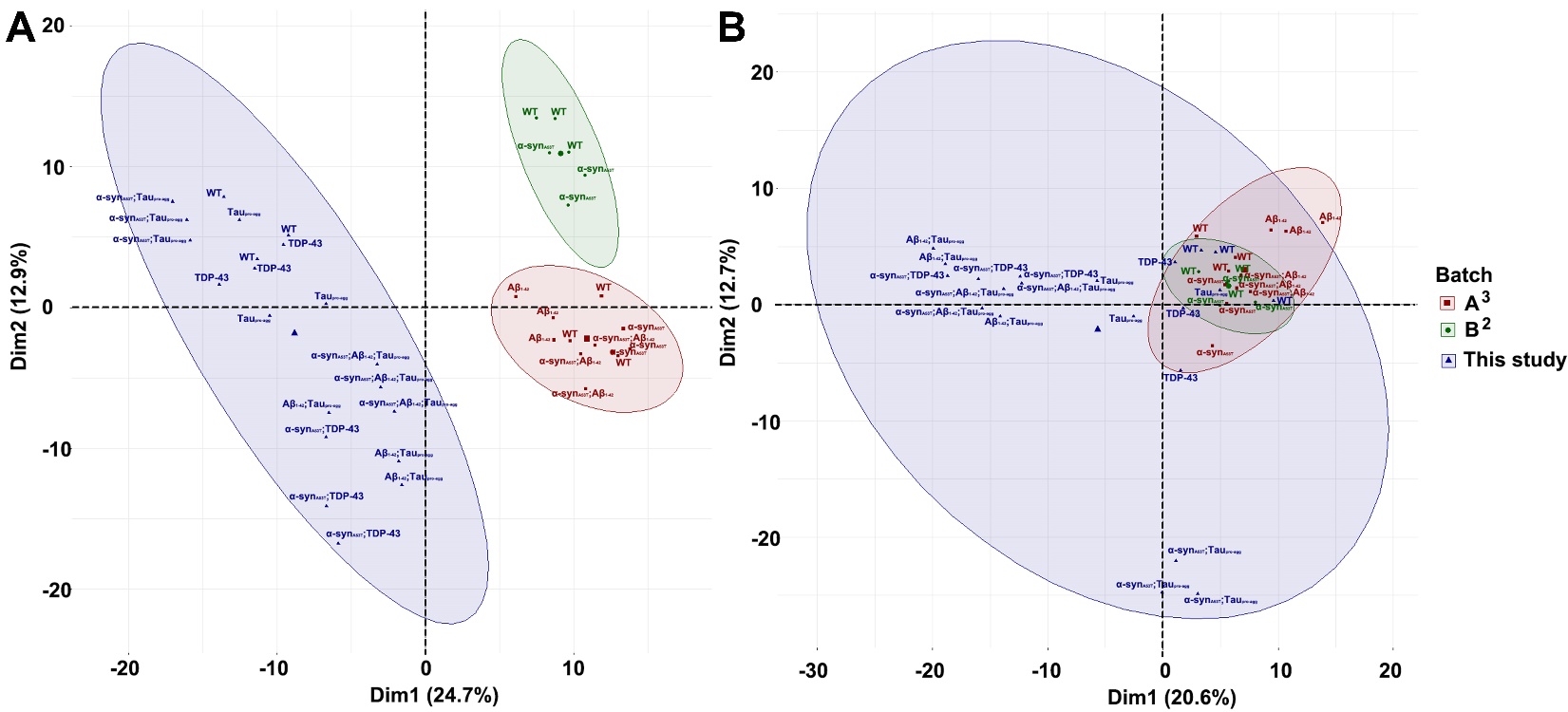


# Figure S6. The principal component analysis (PCA) of all miRNAs sequencing data before (A) and after batch effect correlation (B).


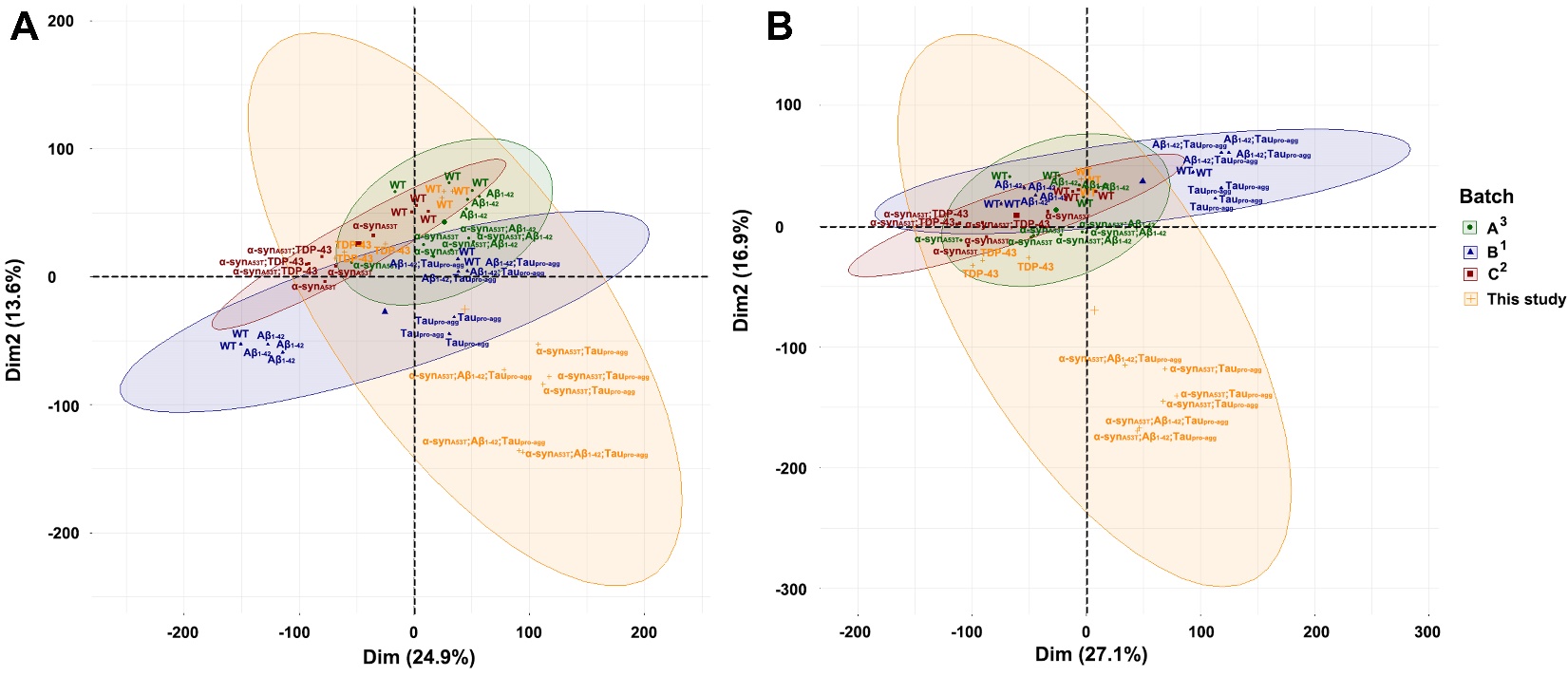


# Figure S7. The principal component analysis (PCA) of all mRNAs sequencing data before (A) and after batch effect correlation (B).

# Table S1. The sequencing data

| **RNA-seq** | | | **Small RNA-seq** | | |
| --- | --- | --- | --- | --- | --- |
| **Samples** | **replicates** | **Reference** | **Samples** | **replicates** | **Reference** |
| WT | 4× | ^1^ | WT | 3× | ^2^ |
| Tau_pro-agg_ | 4× |  | α-syn_A53T_ | 3× |  |
| Aβ_1-42_ | 4× |  | WT | 3× | ^3^ |
| Aβ_1-42_;Tau_pro-agg_ | 4× |  | α-syn_A53T_ | 3× |  |
| WT | 3× | ^2^ | Aβ_1-42_ | 3× |  |
| α-syn_A53T_ | 3× |  | α-syn_A53T_;Aβ_1-42_ | 3× |  |
| α-syn_A53T_;TDP-43 | 3× |  | WT | 3× | This study |
| WT | 3× | ^3^ | Tau_pro-agg_ | 3× |  |
| α-syn_A53T_ | 3× |  | Aβ_1-42_;Tau_pro-agg_ | 3× |  |
| Aβ_1-42_ | 3× |  | TDP-43 | 3× |  |
| α-syn_A53T_;Aβ_1-42_ | 3× |  | α-syn_A53T_;TDP-43 | 3× |  |
| WT | 3× | This study | α-syn_A53T_;Tau_pro-agg_ | 3× |  |
| α-syn_A53T_;Tau_pro-agg_ | 3× |  | α-syn_A53T_;Aβ_1-42_;Tau_pro-agg_ | 3× |  |
| α-syn_A53T_;Aβ_1-42_;Tau_pro-agg_ | 3× |  |  |  |  |

# Table S2. Primers for qRT-PCR

| **Gene** | **Type** | **Sequence** |
| --- | --- | --- |
| *cdc-42* | F | 5'-CTGCTGGACAGGAAGATTACG-3' |
|  | R | 5'-CTCGGACATTCTCGAATGAAG-3' |
| SNCA | F | 5'-GTGGCAACAGTGGCTGAGAAGA-3' |
|  | R | 5'-AATGCTCCCTGCTCCCTCCA-3' |
| TARDBP | F | 5'-CCGAAGATGAGAACGATGA-3' |
|  | R | 5'-CATACACTGAGACACTGGAT-3' |
| MAPT | F | 5'-CTCCACTGAGAACCTGAAG-3' |
|  | R | 5'-GCCTAATGAGCCACACTT-3' |
| APP | F | 5'-CCGACATGACTCAGGATATG-3' |
|  | R | 5'-GCTCACGCTATGACAACA-3' |

# Table S3. The percentage of *Caenorhabditis elegans* with postural defects (Figure 1G).

|  | **Day 1 at 23℃** | | | | **Day 5 at 23℃** | | | |
| --- | --- | --- | --- | --- | --- | --- | --- | --- |
| **Tukey's multiple comparisons test** | **Mean Diff.** | **95% CI of diff.** | **Summary** | **Adjusted *P* Value** | **Mean Diff.** | **95% CI of diff.** | **Summary** | **Adjusted *P* Value** |
| WT vs. Aβ_1-42_ | -26.57 | -35.18 to -17.95 | **** | <0.0001 | -55.61 | -66.23 to -44.99 | **** | <0.0001 |
| WT vs. TDP-43 | 0 | -7.975 to 7.975 | ns | >0.9999 | -41.36 | -51.98 to -30.73 | **** | <0.0001 |
| WT vs. Tau_pro-agg_ | -24.56 | -33.17 to -15.94 | **** | <0.0001 | -61.14 | -71.77 to -50.52 | **** | <0.0001 |
| WT vs. α-syn_A53T_ | -92.99 | -101.0 to -85.01 | **** | <0.0001 | -96.41 | -107.0 to -85.78 | **** | <0.0001 |
| WT vs. α-syn_A53T_;Aβ_1-42_ | -93.36 | -102.0 to -84.75 | **** | <0.0001 | -100 | -110.6 to -89.38 | **** | <0.0001 |
| WT vs. α-syn_A53T_;TDP-43 | -49.4 | -57.37 to -41.42 | **** | <0.0001 | -57.45 | -67.28 to -47.61 | **** | <0.0001 |
| WT vs. Aβ_1-42_;Tau_pro-agg_ | 0 | -7.975 to 7.975 | ns | >0.9999 | -79.46 | -90.08 to -68.84 | **** | <0.0001 |
| WT vs. α-syn_A53T_;Tau_pro-agg_ | 0 | -7.975 to 7.975 | ns | >0.9999 | -51.87 | -62.50 to -41.25 | **** | <0.0001 |
| WT vs. α-syn_A53T_;Aβ_1-42_;Tau_pro-agg_ | -8.889 | -17.50 to -0.2750 | * | 0.0391 | -97.78 | -108.4 to -87.15 | **** | <0.0001 |
| Aβ_1-42_ vs. Aβ_1-42_;Tau_pro-agg_ | 26.57 | 17.95 to 35.18 | **** | <0.0001 | -23.85 | -35.21 to -12.49 | **** | <0.0001 |
| Aβ_1-42_ vs. α-syn_A53T_;Aβ_1-42_ | -66.79 | -76.00 to -57.59 | **** | <0.0001 | -44.39 | -55.75 to -33.03 | **** | <0.0001 |
| Aβ_1-42_ vs. α-syn_A53T_;Aβ_1-42_;Tau_pro-agg_ | 17.68 | 8.468 to 26.89 | **** | <0.0001 | -42.17 | -53.53 to -30.81 | **** | <0.0001 |
| TDP-43 vs. α-syn_A53T_;TDP-43 | -49.4 | -57.37 to -41.42 | **** | <0.0001 | -16.09 | -26.72 to -5.469 | *** | 0.0008 |
| Tau_pro-agg_ vs. Aβ_1-42_;Tau_pro-agg_ | 24.56 | 15.94 to 33.17 | **** | <0.0001 | -18.32 | -29.67 to -6.959 | *** | 0.0004 |
| Tau_pro-agg_ vs. α-syn_A53T_;Tau_pro-agg_ | 24.56 | 15.94 to 33.17 | **** | <0.0001 | 9.27 | -2.087 to 20.63 | ns | 0.1762 |
| Tau_pro-agg_ vs. α-syn_A53T_;Aβ_1-42_;Tau_pro-agg_ | 15.67 | 6.458 to 24.87 | *** | 0.0001 | -36.63 | -47.99 to -25.28 | **** | <0.0001 |
| α-syn_A53T_ vs. α-syn_A53T_;Aβ_1-42_ | -0.3725 | -8.986 to 8.241 | ns | >0.9999 | -3.594 | -14.95 to 7.763 | ns | 0.9784 |
| α-syn_A53T_ vs. α-syn_A53T_;TDP-43 | 43.59 | 35.61 to 51.56 | **** | <0.0001 | 38.96 | 28.33 to 49.58 | **** | <0.0001 |
| α-syn_A53T_ vs. α-syn_A53T_;Tau_pro-agg_ | 92.99 | 85.01 to 101.0 | **** | <0.0001 | 44.53 | 33.18 to 55.89 | **** | <0.0001 |
| α-syn_A53T_ vs. α-syn_A53T_;Aβ_1-42_;Tau_pro-agg_ | 84.1 | 75.48 to 92.71 | **** | <0.0001 | -1.372 | -12.73 to 9.985 | ns | >0.9999 |
| α-syn_A53T_;Aβ_1-42_ vs. α-syn_A53T_;Aβ_1-42_;Tau_pro-agg_ | 84.47 | 75.26 to 93.68 | **** | <0.0001 | 2.222 | -9.135 to 13.58 | ns | 0.9993 |
| Aβ_1-42_;Tau_pro-agg_ vs. α-syn_A53T_;Aβ_1-42_;Tau_pro-agg_ | -8.889 | -17.50 to -0.2750 | * | 0.0391 | -18.32 | -29.68 to -6.962 | *** | 0.0004 |
| α-syn_A53T_;Tau_pro-agg_ vs. α-syn_A53T_;Aβ_1-42_;Tau_pro-agg_ | -8.889 | -17.50 to -0.2750 | * | 0.0391 | -45.9 | -57.26 to -34.55 | **** | <0.0001 |

# Table S4. The development of *Caenorhabditis elegans* (Figure 2L).

|  | **40 hours at 20℃** | | | | **58 hours at 20℃** | | | | **70 hours at 20℃** | | | |
| --- | --- | --- | --- | --- | --- | --- | --- | --- | --- | --- | --- | --- |
| **Tukey's multiple comparisons test** | **Mean Diff.** | **95% CI of diff.** | **Summary** | **Adjusted *P* Value** | **Mean Diff.** | **95% CI of diff.** | **Summary** | **Adjusted *P* Value** | **Mean Diff.** | **95% CI of diff.** | **Summary** | **Adjusted *P* Value** |
| WT vs. Aβ_1-42_ | 0 | -1.40 to 1.40 | ns | >0.9999 | 0.0067 | -2.100 to 2.113 | ns | >0.9999 | 0.0067 | -1.259 to 1.272 | ns | >0.9999 |
| WT vs. TDP-43 | 0 | -1.40 to 1.40 | ns | >0.9999 | 0 | -2.107 to 2.107 | ns | >0.9999 | 0 | -1.266 to 1.266 | ns | >0.9999 |
| WT vs. Tau_pro-agg_ | 21.99 | 20.59 to 23.39 | **** | <0.0001 | 40.49 | 38.38 to 42.60 | **** | <0.0001 | 6.971 | 5.705 to 8.237 | **** | <0.0001 |
| WT vs. α-syn_A53T_ | 2.891 | 1.49 to 4.291 | **** | <0.0001 | 25.2 | 23.09 to 27.31 | **** | <0.0001 | 1.46 | 0.1943 to 2.726 | * | 0.016 |
| WT vs. α-syn_A53T_;Aβ_1-42_ | 85.25 | 83.85 to 86.65 | **** | <0.0001 | 100 | 97.89 to 102.1 | **** | <0.0001 | 97.54 | 96.27 to 98.81 | **** | <0.0001 |
| WT vs. α-syn_A53T_;TDP-43 | 0 | -1.40 to 1.40 | ns | >0.9999 | 0 | -2.107 to 2.107 | ns | >0.9999 | 0 | -1.266 to 1.266 | ns | >0.9999 |
| WT vs. Aβ_1-42_;Tau_pro-agg_ | 82.7 | 81.30 to 84.10 | **** | <0.0001 | 99.1 | 97.00 to 101.2 | **** | <0.0001 | 94.65 | 93.39 to 95.92 | **** | <0.0001 |
| WT vs. α-syn_A53T_;Tau_pro-agg_ | 66.73 | 65.33 to 68.13 | **** | <0.0001 | 100 | 97.89 to 102.1 | **** | <0.0001 | 94.6 | 93.34 to 95.87 | **** | <0.0001 |
| WT vs. α-syn_A53T_;Aβ_1-42_;Tau_pro-agg_ | 60.16 | 58.76 to 61.56 | **** | <0.0001 | 100 | 97.89 to 102.1 | **** | <0.0001 | 88.37 | 87.10 to 89.63 | **** | <0.0001 |
| Aβ_1-42_ vs. Aβ_1-42_;Tau_pro-agg_ | 82.7 | 81.30 to 84.10 | **** | <0.0001 | 99.1 | 96.99 to 101.2 | **** | <0.0001 | 94.65 | 93.38 to 95.91 | **** | <0.0001 |
| Aβ_1-42_ vs. α-syn_A53T_;Aβ_1-42_ | 85.25 | 83.85 to 86.65 | **** | <0.0001 | 99.99 | 97.89 to 102.1 | **** | <0.0001 | 97.53 | 96.27 to 98.80 | **** | <0.0001 |
| Aβ_1-42_ vs. α-syn_A53T_;Aβ_1-42_;Tau_pro-agg_ | 60.16 | 58.76 to 61.56 | **** | <0.0001 | 99.99 | 97.89 to 102.1 | **** | <0.0001 | 88.36 | 87.09 to 89.63 | **** | <0.0001 |
| TDP-43 vs. α-syn_A53T_;TDP-43 | 0 | -1.400 to 1.40 | ns | >0.9999 | 0 | -2.107 to 2.107 | ns | >0.9999 | 0 | -1.266 to 1.266 | ns | >0.9999 |
| Tau_pro-agg_ vs. Aβ_1-42_;Tau_pro-agg_ | 60.71 | 59.31 to 62.11 | **** | <0.0001 | 58.61 | 56.51 to 60.72 | **** | <0.0001 | 87.68 | 86.41 to 88.95 | **** | <0.0001 |
| Tau_pro-agg_ vs. α-syn_A53T_;Tau_pro-agg_ | 44.74 | 43.34 to 46.14 | **** | <0.0001 | 59.51 | 57.40 to 61.62 | **** | <0.0001 | 87.63 | 86.36 to 88.90 | **** | <0.0001 |
| Tau_pro-agg_ vs. α-syn_A53T_;Aβ_1-42_;Tau_pro-agg_ | 38.17 | 36.77 to 39.57 | **** | <0.0001 | 59.51 | 57.40 to 61.62 | **** | <0.0001 | 81.4 | 80.13 to 82.66 | **** | <0.0001 |
| α-syn_A53T_ vs. α-syn_A53T_;Aβ_1-42_ | 82.36 | 80.96 to 83.75 | **** | <0.0001 | 74.8 | 72.69 to 76.91 | **** | <0.0001 | 96.08 | 94.81 to 97.35 | **** | <0.0001 |
| α-syn_A53T_ vs. α-syn_A53T_;TDP-43 | -2.891 | -4.291 to -1.49 | **** | <0.0001 | -25.2 | -27.31 to -23.09 | **** | <0.0001 | -1.46 | -2.726 to -0.1943 | * | 0.016 |
| α-syn_A53T_ vs. α-syn_A53T_;Tau_pro-agg_ | 63.84 | 62.44 to 65.24 | **** | <0.0001 | 74.8 | 72.69 to 76.91 | **** | <0.0001 | 93.14 | 91.88 to 94.41 | **** | <0.0001 |
| α-syn_A53T_ vs. α-syn_A53T_;Aβ_1-42_;Tau_pro-agg_ | 57.27 | 55.87 to 58.67 | **** | <0.0001 | 74.8 | 72.69 to 76.91 | **** | <0.0001 | 86.91 | 85.64 to 88.17 | **** | <0.0001 |
| α-syn_A53T_;Aβ_1-42_ vs. α-syn_A53T_;Aβ_1-42_;Tau_pro-agg_ | -25.09 | -26.49 to -23.69 | **** | <0.0001 | 0 | -2.107 to 2.107 | ns | >0.9999 | -9.173 | -10.44 to -7.907 | **** | <0.0001 |
| Aβ_1-42_;Tau_pro-agg_ vs. α-syn_A53T_;Aβ_1-42_;Tau_pro-agg_ | -22.53 | -23.93 to -21.13 | **** | <0.0001 | 0.90 | -1.210 to 3.003 | ns | 0.8742 | -6.285 | -7.550 to -5.019 | **** | <0.0001 |
| α-syn_A53T_;Tau_pro-agg_ vs. α-syn_A53T_;Aβ_1-42_;Tau_pro-agg_ | -6.566 | -7.97 to -5.17 | **** | <0.0001 | 0 | -2.107 to 2.107 | ns | >0.9999 | -6.235 | -7.500 to -4.969 | **** | <0.0001 |

# Table S5. The thrashing of *Caenorhabditis elegans* (Figure 3A).

| **Tukey's multiple comparisons test** | **Day 1 at 23℃** | | | | **Day 5 at 23℃** | | | |
| --- | --- | --- | --- | --- | --- | --- | --- | --- |
|  | **Mean Diff.** | **95% CI of diff.** | **Summary** | **Adjusted *P* Value** | **Mean Diff.** | **95% CI of diff.** | **Summary** | **Adjusted *P* Value** |
| WT vs Aβ_1-42_ | -5.295 | -7.803 to -2.788 | **** | <0.0001 | 0.1327 | -3.624 to 3.889 | ns | >0.9999 |
| WT vs TDP-43 | 29.06 | 26.73 to 31.39 | **** | <0.0001 | 24.98 | 22.28 to 27.69 | **** | <0.0001 |
| WT vs Tau_pro-agg_ | -6.505 | -8.873 to -4.137 | **** | <0.0001 | -9.686 | -12.65 to -6.719 | **** | <0.0001 |
| WT vs α-syn_A53T_ | 20.62 | 18.29 to 22.95 | **** | <0.0001 | 20.56 | 17.54 to 23.58 | **** | <0.0001 |
| WT vs α-syn_A53T_;Aβ_1-42_ | 29.22 | 26.75 to 31.69 | **** | <0.0001 | 27.15 | 23.52 to 30.77 | **** | <0.0001 |
| WT vs α-syn_A53T_;TDP-43 | 9.936 | 7.148 to 12.72 | **** | <0.0001 | 21.11 | 18.07 to 24.14 | **** | <0.0001 |
| WT vs Aβ_1-42_;Tau_pro-agg_ | -24.83 | -27.50 to -22.16 | **** | <0.0001 | -12.3 | -15.17 to -9.426 | **** | <0.0001 |
| WT vs α-syn_A53T_;Tau_pro-agg_ | -19.26 | -21.71 to -16.81 | **** | <0.0001 | -12.98 | -15.90 to -10.06 | **** | <0.0001 |
| WT vs α-syn_A53T_;Aβ_1-42_;Tau_pro-agg_ | 9.718 | 6.845 to 12.59 | **** | <0.0001 | 6.705 | 3.286 to 10.12 | **** | <0.0001 |
| Aβ_1-42_ vs α-syn_A53T_;Aβ_1-42_ | 34.51 | 32.02 to 37.00 | **** | <0.0001 | 27.01 | 22.66 to 31.37 | **** | <0.0001 |
| Aβ_1-42_ vs Aβ_1-42_;Tau_pro-agg_ | -19.54 | -22.22 to -16.85 | **** | <0.0001 | -12.43 | -16.18 to -8.684 | **** | <0.0001 |
| Aβ_1-42_ vs α-syn_A53T_;Aβ_1-42_;Tau_pro-agg_ | 15.01 | 12.12 to 17.90 | **** | <0.0001 | 6.572 | 2.391 to 10.75 | **** | <0.0001 |
| TDP-43 vs α-syn_A53T_;TDP-43 | -19.13 | -21.78 to -16.48 | **** | <0.0001 | -3.877 | -6.747 to -1.008 | *** | 0.0009 |
| Tau_pro-agg_ vs Aβ_1-42_;Tau_pro-agg_ | -18.33 | -20.88 to -15.77 | **** | <0.0001 | -2.614 | -5.571 to 0.3431 | ns | 0.1362 |
| Tau_pro-agg_ vs α-syn_A53T_;Tau_pro-agg_ | -12.75 | -15.08 to -10.42 | **** | <0.0001 | -3.295 | -6.295 to -0.2949 | * | 0.0186 |
| Tau_pro-agg_ vs α-syn_A53T_;Aβ_1-42_;Tau_pro-agg_ | 16.22 | 13.45 to 18.99 | **** | <0.0001 | 16.39 | 12.90 to 19.88 | **** | <0.0001 |
| α-syn_A53T_ vs α-syn_A53T_;Aβ_1-42_ | 8.596 | 6.286 to 10.91 | **** | <0.0001 | 6.584 | 2.847 to 10.32 | **** | <0.0001 |
| α-syn_A53T_ vs α-syn_A53T_;TDP-43 | -10.69 | -13.33 to -8.037 | **** | <0.0001 | 0.5448 | -2.624 to 3.714 | ns | >0.9999 |
| α-syn_A53T_ vs α-syn_A53T_;Tau_pro-agg_ | -39.88 | -42.17 to -37.59 | **** | <0.0001 | -33.54 | -36.60 to -30.49 | **** | <0.0001 |
| α-syn_A53T_ vs α-syn_A53T_;Aβ_1-42_;Tau_pro-agg_ | -10.9 | -13.64 to -8.166 | **** | <0.0001 | -13.86 | -17.39 to -10.32 | **** | <0.0001 |
| α-syn_A53T_;Aβ_1-42_ vs α-syn_A53T_;Aβ_1-42_;Tau_pro-agg_ | -19.5 | -22.36 to -16.64 | **** | <0.0001 | -20.44 | -24.50 to -16.38 | **** | <0.0001 |
| Aβ_1-42_;Tau_pro-agg_ vs α-syn_A53T_;Aβ_1-42_;Tau_pro-agg_ | 34.55 | 31.52 to 37.58 | **** | <0.0001 | 19.01 | 15.59 to 22.42 | **** | <0.0001 |
| α-syn_A53T_;Tau_pro-agg_ vs α-syn_A53T_;Aβ_1-42_;Tau_pro-agg_ | 28.98 | 26.13 to 31.82 | **** | <0.0001 | 19.69 | 16.24 to 23.13 | **** | <0.0001 |

# Table S6. The locomotion of *Caenorhabditis elegans* (Figure 3B).

| **Tukey's multiple comparisons test** | **Day 1 at 23℃** | | | | **Day 5 at 23℃** | | | |
| --- | --- | --- | --- | --- | --- | --- | --- | --- |
|  | **Mean Diff.** | **95% CI of diff.** | **Summary** | **Adjusted *P* Value** | **Mean Diff.** | **95% CI of diff.** | **Summary** | **Adjusted *P* Value** |
| WT vs Aβ_1-42_ | 1.947 | 0.8673 to 3.026 | **** | <0.0001 | 3.444 | 2.442 to 4.447 | **** | <0.0001 |
| WT vs TDP-43 | 2.88 | 1.605 to 4.154 | **** | <0.0001 | 5.399 | 4.715 to 6.082 | **** | <0.0001 |
| WT vs Tau_pro-agg_ | 2.733 | 1.620 to 3.846 | **** | <0.0001 | 4.344 | 3.518 to 5.171 | **** | <0.0001 |
| WT vs α-syn_A53T_ | 1.363 | 0.2449 to 2.482 | ** | 0.0047 | 5.772 | 5.063 to 6.481 | **** | <0.0001 |
| WT vs α-syn_A53T_;Aβ_1-42_ | 5.219 | 3.889 to 6.549 | **** | <0.0001 | 5.885 | 5.068 to 6.702 | **** | <0.0001 |
| WT vs α-syn_A53T_;TDP-43 | 3.961 | 2.859 to 5.063 | **** | <0.0001 | 5.028 | 4.311 to 5.744 | **** | <0.0001 |
| WT vs Aβ_1-42_;Tau_pro-agg_ | -10.31 | -11.78 to -8.834 | **** | <0.0001 | -0.01984 | -0.9259 to 0.8862 | ns | >0.9999 |
| WT vs α-syn_A53T_;Tau_pro-agg_ | -1.687 | -2.77 to -0.6055 | **** | <0.0001 | 4.525 | 3.836 to 5.214 | **** | <0.0001 |
| WT vs α-syn_A53T_;Aβ_1-42_;Tau_pro-agg_ | 2.702 | 1.337 to 4.067 | **** | <0.0001 | 3.865 | 3.044 to 4.686 | **** | <0.0001 |
| Aβ_1-42_ vs α-syn_A53T_;Aβ_1-42_ | 3.272 | 2.095 to 4.450 | **** | <0.0001 | 2.44 | 1.464 to 3.417 | **** | <0.0001 |
| Aβ_1-42_ vs Aβ_1-42_;Tau_pro-agg_ | -12.25 | -13.59 to -10.92 | **** | <0.0001 | -3.464 | -4.517 to -2.412 | **** | <0.0001 |
| Aβ_1-42_ vs α-syn_A53T_;Aβ_1-42_;Tau_pro-agg_ | 0.7553 | -0.4612 to 1.972 | ns | 0.6192 | 0.4207 | -0.5600 to 1.401 | ns | 0.938 |
| TDP-43 vs α-syn_A53T_;TDP-43 | 1.082 | -0.0545 to 2.218 | ns | 0.0774 | -0.3708 | -0.8824 to 0.1407 | ns | 0.3891 |
| Tau_pro-agg_ vs Aβ_1-42_;Tau_pro-agg_ | -13.04 | -14.40 to -11.68 | **** | <0.0001 | -4.364 | -5.250 to -3.478 | **** | <0.0001 |
| Tau_pro-agg_ vs α-syn_A53T_;Tau_pro-agg_ | -4.42 | -5.348 to -3.493 | **** | <0.0001 | 0.1804 | -0.4820 to 0.8427 | ns | 0.9974 |
| Tau_pro-agg_ vs α-syn_A53T_;Aβ_1-42_;Tau_pro-agg_ | -0.03125 | -1.277 to 1.215 | ns | >0.9999 | -0.4793 | -1.278 to 0.3199 | ns | 0.666 |
| α-syn_A53T_ vs α-syn_A53T_;Aβ_1-42_ | 3.856 | 2.642 to 5.069 | **** | <0.0001 | 0.1127 | -0.5593 to 0.7847 | ns | >0.9999 |
| α-syn_A53T_ vs α-syn_A53T_;TDP-43 | 2.598 | 1.640 to 3.556 | **** | <0.0001 | -0.7444 | -1.290 to -0.1989 | *** | 0.0007 |
| α-syn_A53T_ vs α-syn_A53T_;Tau_pro-agg_ | -3.051 | -3.985 to -2.116 | **** | <0.0001 | -1.247 | -1.756 to -0.7384 | **** | <0.0001 |
| α-syn_A53T_ vs α-syn_A53T_;Aβ_1-42_;Tau_pro-agg_ | 1.339 | 0.08735 to 2.590 | * | 0.0251 | -1.907 | -2.585 to -1.230 | **** | <0.0001 |
| α-syn_A53T_;Aβ_1-42_ vs α-syn_A53T_;Aβ_1-42_;Tau_pro-agg_ | -2.517 | -3.960 to -1.073 | **** | <0.0001 | -2.02 | -2.809 to -1.230 | **** | <0.0001 |
| Aβ_1-42_;Tau_pro-agg_ vs α-syn_A53T_;Aβ_1-42_;Tau_pro-agg_ | 13.01 | 11.43 to 14.58 | **** | <0.0001 | 3.885 | 3.003 to 4.767 | **** | <0.0001 |
| α-syn_A53T_;Tau_pro-agg_ vs α-syn_A53T_;Aβ_1-42_;Tau_pro-agg_ | -4.389 | -5.608 to -3.171 | **** | <0.0001 | -0.6596 | -1.316 to -0.003265 | * | 0.0477 |

# Table S7. The worm bags fractions of *Caenorhabditis elegans* (Figure 3C).

| **Tukey's multiple comparisons test** | **Day 5 at 20℃** | | | | **Day 5 at 23℃** | | | |
| --- | --- | --- | --- | --- | --- | --- | --- | --- |
|  | **Mean Diff.** | **95% CI of diff.** | **Summary** | **Adjusted *P* Value** | **Mean Diff.** | **95% CI of diff.** | **Summary** | **Adjusted *P* Value** |
| WT vs Aβ_1-42_ | -0.6667 | -17.87 to 16.54 | ns | >0.9999 | 0 | -13.91 to 13.91 | ns | >0.9999 |
| WT vs TDP-43 | -21.12 | -37.21 to -5.031 | ** | 0.0039 | -69.72 | -83.63 to -55.80 | **** | <0.0001 |
| WT vs Tau_pro-agg_ | 0 | -17.20 to 17.20 | ns | >0.9999 | -2.346 | -16.26 to 11.57 | ns | 0.9999 |
| WT vs α-syn_A53T_ | -4.777 | -21.98 to 12.43 | ns | 0.992 | -44.89 | -58.80 to -30.97 | **** | <0.0001 |
| WT vs α-syn_A53T_;Aβ_1-42_ | -22.27 | -39.47 to -5.067 | ** | 0.0046 | -87.4 | -100.4 to -74.38 | **** | <0.0001 |
| WT vs α-syn_A53T_;TDP-43 | -42.42 | -59.62 to -25.22 | **** | <0.0001 | -39 | -51.44 to -26.55 | **** | <0.0001 |
| WT vs Aβ_1-42_;Tau_pro-agg_ | -6.94 | -24.14 to 10.26 | ns | 0.917 | -27.43 | -40.45 to -14.42 | **** | <0.0001 |
| WT vs α-syn_A53T_;Tau_pro-agg_ | -78.25 | -93.64 to -62.87 | **** | <0.0001 | -83.55 | -95.09 to -72.01 | **** | <0.0001 |
| WT vs α-syn_A53T_;Aβ_1-42_;Tau_pro-agg_ | -43.13 | -58.52 to -27.75 | **** | <0.0001 | -34.08 | -47.99 to -20.16 | **** | <0.0001 |
| Aβ_1-42_ vs α-syn_A53T_;Aβ_1-42_ | -21.6 | -38.80 to -4.400 | ** | 0.0064 | -87.4 | -100.4 to -74.38 | **** | <0.0001 |
| Aβ_1-42_ vs Aβ_1-42_;Tau_pro-agg_ | -6.273 | -23.48 to 10.93 | ns | 0.9532 | -27.43 | -40.45 to -14.42 | **** | <0.0001 |
| Aβ_1-42_ vs α-syn_A53T_;Aβ_1-42_;Tau_pro-agg_ | -42.47 | -57.85 to -27.08 | **** | <0.0001 | -34.08 | -47.99 to -20.16 | **** | <0.0001 |
| TDP-43 vs α-syn_A53T_;TDP-43 | -21.3 | -37.39 to -5.205 | ** | 0.0036 | 30.72 | 18.28 to 43.17 | **** | <0.0001 |
| Tau_pro-agg_ vs Aβ_1-42_;Tau_pro-agg_ | -6.94 | -24.14 to 10.26 | ns | 0.917 | -25.09 | -38.10 to -12.07 | **** | <0.0001 |
| Tau_pro-agg_ vs α-syn_A53T_;Tau_pro-agg_ | -78.25 | -93.64 to -62.87 | **** | <0.0001 | -81.2 | -92.74 to -69.67 | **** | <0.0001 |
| Tau_pro-agg_ vs α-syn_A53T_;Aβ_1-42_;Tau_pro-agg_ | -43.13 | -58.52 to -27.75 | **** | <0.0001 | -31.73 | -45.64 to -17.82 | **** | <0.0001 |
| α-syn_A53T_ vs α-syn_A53T_;Aβ_1-42_ | -17.49 | -34.69 to -0.2898 | * | 0.044 | -42.51 | -55.53 to -29.50 | **** | <0.0001 |
| α-syn_A53T_ vs α-syn_A53T_;TDP-43 | -37.64 | -54.84 to -20.44 | **** | <0.0001 | 5.891 | -6.555 to 18.34 | ns | 0.8285 |
| α-syn_A53T_ vs α-syn_A53T_;Tau_pro-agg_ | -73.48 | -88.86 to -58.09 | **** | <0.0001 | -38.66 | -50.20 to -27.12 | **** | <0.0001 |
| α-syn_A53T_ vs α-syn_A53T_;Aβ_1-42_;Tau_pro-agg_ | -38.36 | -53.74 to -22.97 | **** | <0.0001 | 10.81 | -3.101 to 24.73 | ns | 0.2372 |
| α-syn_A53T_;Aβ_1-42_ vs α-syn_A53T_;Aβ_1-42_;Tau_pro-agg_ | -20.86 | -36.25 to -5.479 | ** | 0.0027 | 53.32 | 40.31 to 66.34 | **** | <0.0001 |
| Aβ_1-42_;Tau_pro-agg_ vs α-syn_A53T_;Aβ_1-42_;Tau_pro-agg_ | -36.19 | -51.58 to -20.81 | **** | <0.0001 | -6.643 | -19.66 to 6.373 | ns | 0.7615 |
| α-syn_A53T_;Tau_pro-agg_ vs α-syn_A53T_;Aβ_1-42_;Tau_pro-agg_ | 35.12 | 21.79 to 48.44 | **** | <0.0001 | 49.48 | 37.94 to 61.01 | **** | <0.0001 |

# Table S8. The egg laying of *Caenorhabditis elegans* (Figure 2S, Day 2 at 23℃).

| **Tukey's multiple comparisons test** | **M9 Buffer** | | | | **5 mg/mL Serotonin** | | | | **10 mg/mL Levamisole** | | | |
| --- | --- | --- | --- | --- | --- | --- | --- | --- | --- | --- | --- | --- |
|  | **Mean Diff.** | **95% CI of diff.** | **Summary** | **Adjusted *P* Value** | **Mean Diff.** | **95% CI of diff.** | **Summary** | **Adjusted *P* Value** | **Mean Diff.** | **95% CI of diff.** | **Summary** | **Adjusted *P* Value** |
| WT vs. Aβ_1-42_ | -8.623 | -10.82 to -6.427 | **** | <0.0001 | 28.5 | 25.09 to 31.91 | **** | <0.0001 | 2.484 | 0.5732 to 4.395 | ** | 0.0018 |
| WT vs. TDP-43 | -4.598 | -7.346 to -1.850 | **** | <0.0001 | 24.78 | 20.67 to 28.89 | **** | <0.0001 | -2.524 | -5.120 to 0.07257 | ns | 0.0645 |
| WT vs. Tau_pro-agg_ | 1.599 | -0.7108 to 3.909 | ns | 0.4515 | 45.88 | 41.44 to 50.32 | **** | <0.0001 | 7.651 | 5.281 to 10.02 | **** | <0.0001 |
| WT vs. α-syn_A53T_ | -3.698 | -5.841 to -1.555 | **** | <0.0001 | 41.82 | 37.89 to 45.75 | **** | <0.0001 | 6.941 | 5.070 to 8.812 | **** | <0.0001 |
| WT vs. α-syn_A53T_;Aβ_1-42_ | -6.025 | -8.240 to -3.809 | **** | <0.0001 | 40.6 | 36.89 to 44.32 | **** | <0.0001 | 8.284 | 6.101 to 10.47 | **** | <0.0001 |
| WT vs. α-syn_A53T_;TDP-43 | 0.3889 | -3.285 to 4.063 | ns | >0.9999 | 27.38 | 22.94 to 31.82 | **** | <0.0001 | 2.238 | -0.01044 to 4.487 | ns | 0.0522 |
| WT vs. Aβ_1-42_;Tau_pro-agg_ | 2.448 | 0.2528 to 4.644 | * | 0.0159 | 39.72 | 35.17 to 44.27 | **** | <0.0001 | 5.895 | 3.365 to 8.426 | **** | <0.0001 |
| WT vs. α-syn_A53T_;Tau_pro-agg_ | 1.806 | -0.3216 to 3.933 | ns | 0.175 | 46.82 | 43.29 to 50.36 | **** | <0.0001 | 9.54 | 7.464 to 11.61 | **** | <0.0001 |
| WT vs. α-syn_A53T_;Aβ_1-42_;Tau_pro-agg_ | 2.46 | 0.09185 to 4.829 | * | 0.0346 | 52.14 | 48.21 to 56.07 | **** | <0.0001 | 3.473 | 0.9424 to 6.004 | *** | 0.0007 |
| Aβ_1-42_ vs. α-syn_A53T_;Aβ_1-42_ | 2.598 | 0.4027 to 4.794 | ** | 0.0075 | 12.1 | 8.692 to 15.51 | **** | <0.0001 | 5.8 | 3.554 to 8.045 | **** | <0.0001 |
| Aβ_1-42_ vs. Aβ_1-42_;Tau_pro-agg_ | 11.07 | 8.896 to 13.25 | **** | <0.0001 | 11.22 | 6.913 to 15.52 | **** | <0.0001 | 3.411 | 0.8256 to 5.997 | ** | 0.0014 |
| Aβ_1-42_ vs. α-syn_A53T_;Aβ_1-42_;Tau_pro-agg_ | 11.08 | 8.733 to 13.43 | **** | <0.0001 | 23.64 | 20.00 to 27.28 | **** | <0.0001 | 0.9889 | -1.597 to 3.574 | ns | 0.9683 |
| TDP-43 vs. α-syn_A53T_;TDP-43 | 4.987 | 0.9696 to 9.005 | ** | 0.0038 | 2.595 | -2.178 to 7.368 | ns | 0.7721 | 4.762 | 1.859 to 7.665 | **** | <0.0001 |
| Tau_pro-agg_ vs. Aβ_1-42_;Tau_pro-agg_ | 0.8494 | -1.441 to 3.140 | ns | 0.974 | -6.159 | -11.32 to -1.000 | ** | 0.0067 | -1.756 | -4.697 to 1.186 | ns | 0.6649 |
| Tau_pro-agg_ vs. α-syn_A53T_;Tau_pro-agg_ | 0.2065 | -2.019 to 2.432 | ns | >0.9999 | 0.9464 | -3.346 to 5.239 | ns | 0.9995 | 1.889 | -0.6712 to 4.449 | ns | 0.357 |
| Tau_pro-agg_ vs. α-syn_A53T_;Aβ_1-42_;Tau_pro-agg_ | 0.8613 | -1.596 to 3.318 | ns | 0.9822 | 6.262 | 1.640 to 10.88 | *** | 0.0009 | -4.178 | -7.119 to -1.236 | *** | 0.0004 |
| α-syn_A53T_ vs. α-syn_A53T_;Aβ_1-42_ | -2.326 | -4.469 to -0.1836 | * | 0.0216 | -1.219 | -5.149 to 2.711 | ns | 0.9925 | 1.342 | -0.8696 to 3.554 | ns | 0.643 |
| α-syn_A53T_ vs. α-syn_A53T_;TDP-43 | 4.087 | 0.4565 to 7.718 | * | 0.0142 | -14.44 | -19.07 to -9.823 | **** | <0.0001 | -4.703 | -6.980 to -2.426 | **** | <0.0001 |
| α-syn_A53T_ vs. α-syn_A53T_;Tau_pro-agg_ | 5.504 | 3.452 to 7.555 | **** | <0.0001 | 5.002 | 1.240 to 8.764 | ** | 0.0013 | 2.598 | 0.4920 to 4.705 | ** | 0.0042 |
| α-syn_A53T_ vs. α-syn_A53T_;Aβ_1-42_;Tau_pro-agg_ | 6.159 | 3.858 to 8.459 | **** | <0.0001 | 10.32 | 6.184 to 14.45 | **** | <0.0001 | -3.468 | -6.025 to -0.9122 | *** | 0.0009 |
| α-syn_A53T_;Aβ_1-42_ vs. α-syn_A53T_;Aβ_1-42_;Tau_pro-agg_ | 8.485 | 6.117 to 10.85 | **** | <0.0001 | 11.54 | 7.607 to 15.47 | **** | <0.0001 | -4.811 | -7.603 to -2.018 | **** | <0.0001 |
| Aβ_1-42_;Tau_pro-agg_ vs. α-syn_A53T_;Aβ_1-42_;Tau_pro-agg_ | 0.0119 | -2.338 to 2.362 | ns | >0.9999 | 12.42 | 7.694 to 17.15 | **** | <0.0001 | -2.422 | -5.494 to 0.6499 | ns | 0.2642 |
| α-syn_A53T_;Tau_pro-agg_ vs. α-syn_A53T_;Aβ_1-42_;Tau_pro-agg_ | 0.6548 | -1.631 to 2.941 | ns | 0.9959 | 5.315 | 1.554 to 9.077 | *** | 0.0004 | -6.067 | -8.776 to -3.357 | **** | <0.0001 |

# Table S9. The egg laying of *Caenorhabditis elegans* (Figure 3D, Day 2 at 23℃).

| **Tukey's multiple comparisons test** | **5 mg/mL Serotonin** | | | | **10 mg/mL Levamisole** | | | |
| --- | --- | --- | --- | --- | --- | --- | --- | --- |
|  | **Mean Diff.** | **95% CI of diff.** | **Summary** | **Adjusted *P* Value** | **Mean Diff.** | **95% CI of diff.** | **Summary** | **Adjusted *P* Value** |
| WT vs Aβ_1-42_ | 34.62 | 29.72 to 39.53 | **** | <0.0001 | 9.724 | 4.729 to 14.72 | **** | <0.0001 |
| WT vs TDP-43 | 28.38 | 22.63 to 34.13 | **** | <0.0001 | 0.8589 | -5.713 to 7.431 | ns | >0.9999 |
| WT vs Tau_pro-agg_ | 45.03 | 38.46 to 51.60 | **** | <0.0001 | 6.902 | 0.3303 to 13.47 | * | 0.0275 |
| WT vs α-syn_A53T_ | 48.92 | 43.28 to 54.57 | **** | <0.0001 | 10.98 | 5.429 to 16.53 | **** | <0.0001 |
| WT vs α-syn_A53T_;Aβ_1-42_ | 47.92 | 42.00 to 53.85 | **** | <0.0001 | 14.53 | 8.402 to 20.66 | **** | <0.0001 |
| WT vs α-syn_A53T_;TDP-43 | 29.07 | 22.60 to 35.54 | **** | <0.0001 | 2.741 | -3.731 to 9.213 | ns | 0.9946 |
| WT vs Aβ_1-42_;Tau_pro-agg_ | 36.92 | 30.13 to 43.72 | **** | <0.0001 | 4.243 | -2.813 to 11.30 | * | 0.0313 |
| WT vs α-syn_A53T_;Tau_pro-agg_ | 45.37 | 40.02 to 50.72 | **** | <0.0001 | 8.811 | 3.166 to 14.46 | **** | <0.0001 |
| WT vs α-syn_A53T_;Aβ_1-42_;Tau_pro-agg_ | 49.24 | 42.77 to 55.71 | **** | <0.0001 | 1.215 | -5.465 to 7.894 | ns | >0.9999 |
| Aβ_1-42_ vs α-syn_A53T_;Aβ_1-42_ | 13.3 | 7.340 to 19.26 | **** | <0.0001 | 4.809 | -1.426 to 11.04 | ns | 0.3974 |
| Aβ_1-42_ vs Aβ_1-42_;Tau_pro-agg_ | 2.301 | -4.524 to 9.126 | ns | 0.9997 | -5.48 | -12.63 to 1.667 | ns | 0.409 |
| Aβ_1-42_ vs α-syn_A53T_;Aβ_1-42_;Tau_pro-agg_ | 14.62 | 8.114 to 21.12 | **** | <0.0001 | -8.509 | -15.28 to -1.734 | ** | 0.0016 |
| TDP-43 vs α-syn_A53T_;TDP-43 | 0.6936 | -6.467 to 7.854 | ns | >0.9999 | 1.882 | -5.955 to 9.720 | ns | >0.9999 |
| Tau_pro-agg_ vs Aβ_1-42_;Tau_pro-agg_ | -8.104 | -16.21 to 0.00318 | ns | 0.0502 | -2.659 | -10.99 to 5.668 | ns | 0.9999 |
| Tau_pro-agg_ vs α-syn_A53T_;Tau_pro-agg_ | 0.3428 | -6.596 to 7.281 | ns | >0.9999 | 1.908 | -5.262 to 9.078 | ns | >0.9999 |
| Tau_pro-agg_ vs α-syn_A53T_;Aβ_1-42_;Tau_pro-agg_ | 4.212 | -3.625 to 12.05 | ns | 0.9332 | -5.688 | -13.70 to 2.322 | ns | 0.5634 |
| α-syn_A53T_ vs α-syn_A53T_;Aβ_1-42_ | -0.9991 | -7.580 to 5.582 | ns | >0.9999 | 3.553 | -3.137 to 10.24 | * | 0.0399 |
| α-syn_A53T_ vs α-syn_A53T_;TDP-43 | -19.85 | -26.93 to -12.77 | **** | <0.0001 | -8.239 | -15.24 to -1.236 | ** | 0.0051 |
| α-syn_A53T_ vs α-syn_A53T_;Tau_pro-agg_ | -3.55 | -9.617 to 2.518 | * | 0.042 | -2.17 | -8.416 to 4.077 | * | 0.0485 |
| α-syn_A53T_ vs α-syn_A53T_;Aβ_1-42_;Tau_pro-agg_ | 0.3194 | -6.759 to 7.397 | ns | >0.9999 | -9.766 | -16.96 to -2.570 | *** | 0.0003 |
| α-syn_A53T_;Aβ_1-42_ vs α-syn_A53T_;Aβ_1-42_;Tau_pro-agg_ | 1.319 | -5.984 to 8.621 | ns | >0.9999 | -13.32 | -20.97 to -5.666 | **** | <0.0001 |
| Aβ_1-42_;Tau_pro-agg_ vs α-syn_A53T_;Aβ_1-42_;Tau_pro-agg_ | 12.32 | 4.290 to 20.34 | **** | <0.0001 | -3.029 | -11.44 to 5.383 | * | 0.0293 |
| α-syn_A53T_;Tau_pro-agg_ vs α-syn_A53T_;Aβ_1-42_;Tau_pro-agg_ | 3.869 | -2.974 to 10.71 | ** | 0.0016 | -7.596 | -14.86 to -0.3275 | **** | <0.0001 |

# Table S10. The longevity (Figure 3G, 23℃) and learning index (Figure3E, day 1 at 23℃) of *Caenorhabditis elegans*.

| **Longevity** | | | | **Learning Index** | | | | |
| --- | --- | --- | --- | --- | --- | --- | --- | --- |
| **Log Rank (Mantel-Cox)'s multiple comparisons test** | **Chi-square value** | **Summary** | ***P* value** | **Tukey's multiple comparisons test** | **Mean Diff.** | **95% CI of diff.** | **Summary** | **Adjusted *P* Value** |
| WT vs Aβ_1-42_ | 8.109824109 | ** | 0.004403 | WT vs Aβ_1-42_ | 0.3705 | 0.2094 to 0.5315 | **** | <0.0001 |
| WT vs TDP-43 | 56.601957 | **** | 5.34E-14 | WT vs TDP-43 | 0.3881 | 0.2271 to 0.5491 | **** | <0.0001 |
| WT vs Tau_pro-agg_ | 165.1429438 | **** | 8.51E-38 | WT vs Tau_pro-agg_ | 0.192 | 0.03098 to 0.3530 | * | 0.0136 |
| WT vs α-syn_A53T_ | 523.5678442 | **** | 7.1E-116 |  |  |  |  |  |
| WT vs α-syn_A53T_;Aβ_1-42_ | 388.1425644 | **** | 2.1E-86 |  |  |  |  |  |
| WT vs α-syn_A53T_;TDP-43 | 157.9463863 | **** | 3.18E-36 | WT vs α-syn_A53T_;TDP-43 | 0.2384 | 0.08778 to 0.3890 | *** | 0.0009 |
| WT vs Aβ_1-42_;Tau_pro-agg_ | 121.1140589 | **** | 3.61E-28 | WT vs Aβ_1-42_;Tau_pro-agg_ | 0.0986 | -0.0624 to 0.2596 | * | 0.0452 |
| WT vs α-syn_A53T_;Tau_pro-agg_ | 315.5340654 | **** | 1.36E-70 | WT vs α-syn_A53T_;Tau_pro-agg_ | 0.3399 | 0.1789 to 0.5010 | **** | <0.0001 |
| WT vs α-syn_A53T_;Aβ_1-42_;Tau_pro-agg_ | 45.32103398 | **** | 1.67E-11 | WT vs α-syn_A53T_;Aβ_1-42_;Tau_pro-agg_ | 0.2248 | 0.06379 to 0.3858 | ** | 0.0033 |
| Aβ_1-42_ vs α-syn_A53T_;Aβ_1-42_ | 581.6092266 | **** | 1.7E-128 |  |  |  |  |  |
| Aβ_1-42_ vs Aβ_1-42_;Tau_pro-agg_ | 80.99643155 | **** | 2.26E-19 | Aβ_1-42_ vs Aβ_1-42_;Tau_pro-agg_ | -0.2719 | -0.4329 to -0.1108 | *** | 0.0005 |
| Aβ_1-42_ vs α-syn_A53T_;Aβ_1-42_;Tau_pro-agg_ | 38.70067749 | **** | 4.94E-10 | Aβ_1-42_ vs α-syn_A53T_;Aβ_1-42_;Tau_pro-agg_ | -0.1456 | -0.3067 to 0.0154 | ns | 0.0926 |
| TDP-43 vs α-syn_A53T_;TDP-43 | 4.559725851 | * | 0.032732 | TDP-43 vs α-syn_A53T_;TDP-43 | -0.1497 | -0.300 to 0.00092 | ns | 0.0521 |
| Tau_pro-agg_ vs Aβ_1-42_;Tau_pro-agg_ | 7.824619308 | ** | 0.005154 | Tau_pro-agg_ vs Aβ_1-42_;Tau_pro-agg_ | -0.09341 | -0.2544 to 0.0676 | ns | 0.5135 |
| Tau_pro-agg_ vs α-syn_A53T_;Tau_pro-agg_ | 49.22559519 | **** | 2.28E-12 | Tau_pro-agg_ vs α-syn_A53T_;Tau_pro-agg_ | 0.1479 | -0.01309 to 0.309 | ns | 0.0846 |
| Tau_pro-agg_ vs α-syn_A53T_;Aβ_1-42_;Tau_pro-agg_ | 3.331801923 | ns | 0.067952 | Tau_pro-agg_ vs α-syn_A53T_;Aβ_1-42_;Tau_pro-agg_ | 0.03281 | -0.1282 to 0.1938 | ns | 0.9958 |
| α-syn_A53T_ vs α-syn_A53T_;Aβ_1-42_ | 71.4273489 | **** | 2.88E-17 |  |  |  |  |  |
| α-syn_A53T_ vs α-syn_A53T_;TDP-43 | 362.9748895 | **** | 6.34E-81 |  |  |  |  |  |
| α-syn_A53T_ vs α-syn_A53T_;Tau_pro-agg_ | 96.47954702 | **** | 9.02E-23 |  |  |  |  |  |
| α-syn_A53T_ vs α-syn_A53T_;Aβ_1-42_;Tau_pro-agg_ | 252.2723921 | **** | 8.3E-57 |  |  |  |  |  |
| α-syn_A53T_;Aβ_1-42_ vs α-syn_A53T_;Aβ_1-42_;Tau_pro-agg_ | 339.1210365 | **** | 9.92E-76 |  |  |  |  |  |
| Aβ_1-42_;Tau_pro-agg_ vs α-syn_A53T_;Aβ_1-42_;Tau_pro-agg_ | 4.725614076 | * | 0.029717 | Aβ_1-42_;Tau_pro-agg_ vs α-syn_A53T_;Aβ_1-42_;Tau_pro-agg_ | 0.1262 | -0.03480 to 0.2873 | ns | 0.1905 |
| α-syn_A53T_;Tau_pro-agg_ vs α-syn_A53T_;Aβ_1-42_;_Taupro-agg_ | 56.36179757 | **** | 6.03E-14 | α-syn_A53T_;Tau_pro-agg_ vs α-syn_A53T_;Aβ_1-42_;Tau_pro-agg_ | -0.1151 | -0.2762 to 0.04590 | ns | 0.2769 |

# Table S11. Measurements of staying on or off food in *Caenorhabditis elegans* (Figure 4A, day 1 23℃) at time points 15^th^ minute, 30^th^ minute, 45^th^ minute and 60^th^ minute.

| **Tukey's multiple comparisons test** | **15^th^ min** | | | | **30^th^ min** | | | | **45^th^ min** | | | | **60^th^ min** | | | |
| --- | --- | --- | --- | --- | --- | --- | --- | --- | --- | --- | --- | --- | --- | --- | --- | --- |
|  | **Mean Diff.** | **95% CI of diff.** | **Summary** | **Adjusted *P* Value** | **Mean Diff.** | **95% CI of diff.** | **Summary** | **Adjusted *P* Value** | **Mean Diff.** | **95% CI of diff.** | **Summary** | **Adjusted *P* Value** | **Mean Diff.** | **95% CI of diff.** | **Summary** | **Adjusted *P* Value** |
| WT vs *cat-1* | -21.37 | -26.81 to -15.93 | **** | <0.0001 | -24.35 | -33.25 to -15.45 | **** | <0.0001 | -49.71 | -59.61 to -39.82 | **** | <0.0001 | -55.31 | -62.81 to -47.82 | **** | <0.0001 |
| WT vs c*at-2* | -17.92 | -23.80 to -12.04 | **** | <0.0001 | -11.77 | -22.67 to -0.8722 | * | 0.0256 | -14.36 | -24.94 to -3.779 | ** | 0.0018 | -26.33 | -34.34 to -18.32 | **** | <0.0001 |
| WT vs Aβ_1-42_ | 3.66 | -1.781 to 9.101 | ns | 0.4596 | 0.919 | -7.522 to 9.360 | ns | >0.9999 | -0.926 | -10.39 to 8.538 | ns | >0.9999 | 1.077 | -6.089 to 8.242 | ns | >0.9999 |
| WT vs TDP-43 | 0.1225 | -5.319 to 5.564 | ns | >0.9999 | -0.3117 | -9.923 to 9.299 | ns | >0.9999 | -2.123 | -12.02 to 7.775 | ns | 0.9998 | -2.836 | -10.33 to 4.658 | ns | 0.9674 |
| WT vs Tau_pro-agg_ | 5.225 | -0.2165 to 10.67 | ns | 0.0693 | 0.233 | -8.208 to 8.674 | ns | >0.9999 | -2.322 | -11.79 to 7.142 | ns | 0.9991 | -2.253 | -9.419 to 4.912 | ns | 0.9919 |
| WT vs α-syn_A53T_ | -17.29 | -22.73 to -11.84 | **** | <0.0001 | -19.85 | -29.46 to -10.24 | **** | <0.0001 | -31.89 | -42.47 to -21.31 | **** | <0.0001 | -35.28 | -43.29 to -27.27 | **** | <0.0001 |
| WT vs α-synA_53T_; Aβ_1-42_ | -13.39 | -18.83 to -7.944 | **** | <0.0001 | -36.81 | -46.42 to -27.20 | **** | <0.0001 | -26.87 | -36.76 to -16.97 | **** | <0.0001 | -29.68 | -37.69 to -21.67 | **** | <0.0001 |
| WT vs α-syn_A53T_; TDP-43 | -15.07 | -20.51 to -9.626 | **** | <0.0001 | -19.14 | -28.75 to -9.524 | **** | <0.0001 | -17.34 | -27.23 to -7.440 | **** | <0.0001 | -14.73 | -22.74 to -6.719 | **** | <0.0001 |
| WT vs Aβ_1-42_; Tau_pro-agg_ | 2.778 | -2.664 to 8.219 | ns | 0.8129 | -1.77 | -10.67 to 7.128 | ns | 0.9999 | -2.325 | -12.22 to 7.572 | ns | 0.9994 | -2.256 | -9.750 to 5.238 | ns | 0.9943 |
| WT vs α-syn_A53T_;Tau_pro-agg_ | -2.218 | -8.096 to 3.659 | ns | 0.9701 | -12.03 | -21.64 to -2.421 | ** | 0.0055 | -17.88 | -27.78 to -7.983 | **** | <0.0001 | -9.89 | -17.90 to -1.879 | ** | 0.0063 |
| WT vs α-syn_A53T_; Aβ_1-42_;Tau_pro-agg_ | -9.668 | -15.11 to -4.226 | **** | <0.0001 | -17.33 | -26.94 to -7.714 | **** | <0.0001 | -20.1 | -30.68 to -9.519 | **** | <0.0001 | -13.19 | -21.20 to -5.179 | *** | 0.0001 |
| *cat-1* vs α-syn_A53T_ | 4.085 | -1.356 to 9.526 | ns | 0.3029 | 4.498 | -5.113 to 14.11 | ns | 0.8741 | 17.83 | 7.929 to 27.72 | **** | <0.0001 | 20.04 | 12.54 to 27.53 | **** | <0.0001 |
| *cat-1* vs α-syn_A53T_; Aβ_1-42_ | 7.985 | 2.544 to 13.43 | *** | 0.0006 | -12.46 | -22.07 to -2.851 | ** | 0.0036 | 22.85 | 13.68 to 32.01 | **** | <0.0001 | 25.64 | 18.14 to 33.13 | **** | <0.0001 |
| *cat-1* vs α-syn_A53T_; TDP-43 | 6.303 | 0.8610 to 11.74 | * | 0.0124 | 5.215 | -4.396 to 14.83 | ns | 0.7393 | 32.38 | 23.21 to 41.54 | **** | <0.0001 | 40.58 | 33.09 to 48.08 | **** | <0.0001 |
| cat-1 vs α-syn_A53T_;Tau_pro-agg_ | 19.15 | 13.27 to 25.03 | **** | <0.0001 | 12.32 | 2.707 to 21.93 | ** | 0.0042 | 31.83 | 22.67 to 41.00 | **** | <0.0001 | 45.42 | 37.93 to 52.92 | **** | <0.0001 |
| *cat-1* vs α-syn_A53T_; Aβ_1-42_;Tau_pro-agg_ | 11.7 | 6.261 to 17.14 | **** | <0.0001 | 7.025 | -2.586 to 16.64 | ns | 0.331 | 29.61 | 19.72 to 39.51 | **** | <0.0001 | 42.12 | 34.63 to 49.62 | **** | <0.0001 |
| *cat-2* vs α-syn_A53T_ | 0.6367 | -5.241 to 6.514 | ns | >0.9999 | -8.082 | -19.57 to 3.406 | ns | 0.3842 | -17.53 | -28.11 to -6.946 | **** | <0.0001 | -8.943 | -16.95 to -0.9319 | * | 0.0185 |
| *cat-2* vs α-syn_A53T_; Aβ_1-42_ | 4.537 | -1.341 to 10.41 | ns | 0.2668 | -25.04 | -36.53 to -13.55 | **** | <0.0001 | -12.51 | -22.40 to -2.610 | ** | 0.0046 | -3.343 | -11.35 to 4.668 | ns | 0.9374 |
| *cat-2* vs α-syn_A53T_; TDP-43 | 2.854 | -3.023 to 8.732 | ns | 0.8553 | -7.365 | -18.85 to 4.122 | ns | 0.5186 | -2.978 | -12.87 to 6.920 | ns | 0.9948 | 11.6 | 3.592 to 19.61 | *** | 0.0008 |
| *cat-2* vs α-syn_A53T_;Tau_pro-agg_ | 15.7 | 9.420 to 21.99 | **** | <0.0001 | -0.2617 | -11.75 to 11.23 | ns | >0.9999 | -3.52 | -13.42 to 6.377 | ns | 0.9806 | 16.44 | 8.432 to 24.45 | **** | <0.0001 |
| *cat-2* vs α-syn_A53T_; Aβ_1-42_;Tau_pro-agg_ | 8.254 | 2.377 to 14.13 | ** | 0.0011 | -5.555 | -17.04 to 5.932 | ns | 0.8499 | -5.74 | -16.32 to 4.841 | ns | 0.7507 | 13.14 | 5.132 to 21.15 | *** | 0.0001 |
| Aβ_1-42_ vs α-syn_A53T_; Aβ_1-42_ | -17.05 | -22.49 to -11.60 | **** | <0.0001 | -37.73 | -46.92 to -28.54 | **** | <0.0001 | -25.94 | -34.63 to -17.25 | **** | <0.0001 | -30.75 | -37.92 to -23.59 | **** | <0.0001 |
| Aβ_1-42_ vs Aβ_1-42_; Tau_pro-agg_ | -0.8825 | -6.324 to 4.559 | ns | >0.9999 | -2.689 | -11.13 to 5.752 | ns | 0.9908 | -1.399 | -10.09 to 7.294 | ns | >0.9999 | -3.333 | -9.915 to 3.250 | ns | 0.8133 |
| Aβ_1-42_ vs α-syn_A53T_; Aβ_1-42_;Tau_pro-agg_ | -13.33 | -18.77 to -7.886 | **** | <0.0001 | -18.24 | -27.43 to -9.054 | **** | <0.0001 | -19.17 | -28.64 to -9.710 | **** | <0.0001 | -14.27 | -21.43 to -7.101 | **** | <0.0001 |
| TDP-43 vs α-syn_A53T_; TDP-43 | -15.19 | -20.63 to -9.749 | **** | <0.0001 | -18.82 | -29.10 to -8.549 | **** | <0.0001 | -15.22 | -24.38 to -6.052 | **** | <0.0001 | -11.89 | -19.39 to -4.400 | *** | 0.0002 |
| Tau_pro-agg_ vs Aβ_1-42_;Tau_pro-agg_ | -2.448 | -7.889 to 2.994 | ns | 0.906 | -2.003 | -10.44 to 6.438 | ns | 0.9993 | -0.003 | -8.696 to 8.690 | ns | >0.9999 | -0.0025 | -6.585 to 6.580 | ns | >0.9999 |
| Tau_pro-agg_ vs α-syn_A53T_;Tau_pro-agg_ | -7.443 | -13.32 to -1.566 | ** | 0.0044 | -12.26 | -21.45 to -3.075 | ** | 0.0025 | -15.56 | -24.25 to -6.865 | **** | <0.0001 | -7.637 | -14.80 to -0.4710 | * | 0.0288 |
| Tau_pro-agg_ vs α-syn_A53T_; Aβ_1-42_;Tau_pro-agg_ | -14.89 | -20.33 to -9.451 | **** | <0.0001 | -17.56 | -26.75 to -8.368 | **** | <0.0001 | -17.78 | -27.24 to -8.314 | **** | <0.0001 | -10.94 | -18.10 to -3.771 | *** | 0.0004 |
| α-syn_A53T_ vs α-syn_A53T_; Aβ_1-42_ | 3.9 | -1.541 to 9.341 | ns | 0.367 | -16.96 | -27.23 to -6.685 | *** | 0.0001 | 5.019 | -4.878 to 14.92 | ns | 0.8189 | 5.6 | -2.411 to 13.61 | ns | 0.396 |
| α-syn_A53T_ vs α-syn_A53T_; TDP-43 | 2.218 | -3.224 to 7.659 | ns | 0.9494 | 0.7167 | -9.558 to 10.99 | ns | >0.9999 | 14.55 | 4.652 to 24.45 | *** | 0.0006 | 20.55 | 12.54 to 28.56 | **** | <0.0001 |
| α-syn_A53T_ vs α-syn_A53T_;Tau_pro-agg_ | 15.07 | 9.189 to 20.94 | **** | <0.0001 | 7.82 | -2.455 to 18.09 | ns | 0.2779 | 14.01 | 4.109 to 23.90 | ** | 0.001 | 25.39 | 17.38 to 33.40 | **** | <0.0001 |
| α-syn_A53T_ vs α-syn_A53T_; Aβ_1-42_;Tau_pro-agg_ | 7.618 | 2.176 to 13.06 | ** | 0.0012 | 2.527 | -7.748 to 12.80 | ns | 0.999 | 11.79 | 1.206 to 22.37 | * | 0.0185 | 22.09 | 14.08 to 30.10 | **** | <0.0001 |
| α-syn_A53T_; Aβ _1-42_ vs α-syn_A53T_; Aβ 1-42;Tau_pro-agg_ | 3.718 | -1.724 to 9.159 | ns | 0.4366 | 19.49 | 9.212 to 29.76 | **** | <0.0001 | 6.768 | -3.130 to 16.66 | ns | 0.4353 | 16.49 | 8.475 to 24.50 | **** | <0.0001 |
| Aβ_1-42_; Tau_pro-agg_ vs α-syn_A53T_; Aβ_1-42_;Tau_pro-agg_ | -12.45 | -17.89 to -7.004 | **** | <0.0001 | -15.56 | -25.17 to -5.944 | *** | 0.0002 | -17.78 | -27.67 to -7.878 | **** | <0.0001 | -10.93 | -18.43 to -3.440 | *** | 0.0007 |
| α-syn_A53T_; Tau_pro-agg_ vs α-syn_A53T_; Aβ_1-42_;Tau_pro-agg_ | -7.449 | -13.33 to -1.572 | ** | 0.0044 | -5.293 | -15.57 to 4.981 | ns | 0.7941 | -2.22 | -12.12 to 7.677 | ns | 0.9996 | -3.3 | -11.31 to 4.711 | ns | 0.9424 |

# Table S12. The basal slowing response of *Caenorhabditis elegans* (Figure 4B, day 1 at 23℃).

| **Two sample t-test** | **Difference between means ± SEM** | **95% CI of diff.** | **Summary** | ***P* Value** |
| --- | --- | --- | --- | --- |
| WT (food vs no food) | -5.516 ± 0.2817 | -6.076 to -4.956 | **** | <0.0001 |
| Cat-1 (food vs no food) | 1.111 ± 0.3213 | 0.4751 to 1.747 | *** | 0.0007 |
| Cat-2 (food vs no food) | -1.087 ± 0.3343 | -1.751 to -0.4228 | ** | 0.0016 |
| Aβ_1-42_ (food vs no food) | -4.935 ± 0.3628 | -5.663 to -4.207 | **** | <0.0001 |
| TDP-43 (food vs no food) | -5.719 ± 0.2170 | -6.148 to -5.289 | **** | <0.0001 |
| Tau_pro-agg_ (food vs no food) | -6.522 ± 0.2953 | -7.108 to -5.935 | **** | <0.0001 |
| α-syn_A53T_ (food vs no food) | -0.03141 ± 0.2463 | -0.5193 to 0.4564 | ns | 0.8988 |
| α-syn_A53T_; TDP-43 (food vs no food) | -2.951 ± 0.3476 | -3.640 to -2.262 | **** | <0.0001 |
| Aβ_1-42_;Tau_pro-agg_ (food vs no food) | -7.289 ± 0.2659 | -7.817 to -6.762 | **** | <0.0001 |
| α-syn_A53T_;Tau_pro-agg_ (food vs no food) | -4.478 ± 0.3251 | -5.124 to -3.833 | **** | <0.0001 |
| α-syn_A53T_; Aβ_1-42_;Tau_pro-agg_ (food vs no food) | -7.090 ± 0.2317 | -7.551 to -6.629 | **** | <0.0001 |

# Table S13. The locomotion distance of *Caenorhabditis elegans* (Figure 4D, day 1 at 23℃).

| **Tukey's multiple comparisons test** | **Mean Diff.** | **95% CI of diff.** | **Summary** | **Adjusted *P* Value** |
| --- | --- | --- | --- | --- |
| WT vs *cat-1* | -8.521 | -13.18 to -3.862 | *** | 0.0004 |
| WT vs *cat-2* | -7.611 | -11.70 to -3.523 | *** | 0.0003 |
| WT vs Aβ_1-42_ | 3.827 | -0.4390 to 8.093 | * | 0.0383 |
| WT vs TDP-43 | 5.954 | 0.9566 to 10.95 | * | 0.0199 |
| WT vs Tau_pro-agg_ | 1.898 | -2.913 to 6.709 | ns | 0.4365 |
| WT vs α-syn_A53T_ | 4.601 | 0.5129 to 8.689 | * | 0.0277 |
| WT vs α-syn_A53T_;Aβ_1-42_ | 9.748 | 3.748 to 15.75 | ** | 0.0016 |
| WT vs α-syn_A53T_;TDP-43 | 0.4652 | -4.195 to 5.125 | ns | 0.8437 |
| WT vs Aβ_1-42_;Tau_pro-agg_ | -0.157 | -5.155 to 4.841 | ns | 0.9505 |
| WT vs α-syn_A53T_;Tau_pro-agg_ | -0.8843 | -5.025 to 3.256 | ns | 0.6733 |
| WT vs α-syn_A53T_;Aβ_1-42_;Tau_pro-agg_ | 1.584 | -2.119 to 5.288 | ns | 0.3989 |
| *cat-1* vs α-syn_A53T_ | 13.12 | 8.897 to 17.35 | **** | <0.0001 |
| *cat-1* vs α-syn_A53T_;Aβ_1-42_ | 18.27 | 12.18 to 24.36 | **** | <0.0001 |
| *cat-1* vs α-syn_A53T_;TDP-43 | 8.987 | 4.206 to 13.77 | *** | 0.0003 |
| *cat-1* vs α-syn_A53T_;Tau_pro-agg_ | 7.637 | 3.361 to 11.91 | *** | 0.0006 |
| *cat-1* vs α-syn_A53T_;Aβ_1-42_;Tau_pro-agg_ | 10.11 | 6.251 to 13.96 | **** | <0.0001 |
| *cat-2* vs α-syn_A53T_ | 12.21 | 8.627 to 15.80 | **** | <0.0001 |
| *cat-2* vs α-syn_A53T_;Aβ_1-42_ | 17.36 | 11.69 to 23.03 | **** | <0.0001 |
| *cat-2* vs α-syn_A53T_;TDP-43 | 8.077 | 3.851 to 12.30 | *** | 0.0002 |
| *cat-2* vs α-syn_A53T_;Tau_pro-agg_ | 6.727 | 3.082 to 10.37 | *** | 0.0004 |
| *cat-2* vs α-syn_A53T_;Aβ_1-42_;Tau_pro-agg_ | 9.196 | 6.056 to 12.34 | **** | <0.0001 |
| Aβ_1-42_ vs α-syn_A53T_;Aβ_1-42_ | 5.921 | 0.1225 to 11.72 | ** | 0.00454 |
| Aβ_1-42_ vs Aβ_1-42_;Tau_pro-agg_ | -3.984 | -8.738 to 0.7706 | * | 0.03783 |
| Aβ_1-42_ vs α-syn_A53T_;Aβ_1-42_;Tau_pro-agg_ | -2.243 | -5.610 to 1.125 | ns | 0.19 |
| TDP-43 vs α-syn_A53T_;TDP-43 | -5.489 | -10.60 to -0.3783 | * | 0.0355 |
| Tau_pro-agg_ vs Aβ_1-42_;Tau_pro-agg_ | -2.055 | -7.304 to 3.194 | ns | 0.44 |
| Tau_pro-agg_ vs α-syn_A53T_;Tau_pro-agg_ | -2.782 | -7.222 to 1.658 | ns | 0.2173 |
| Tau_pro-agg_ vs α-syn_A53T_;Aβ_1-42_;Tau_pro-agg_ | -0.3137 | -4.349 to 3.722 | ns | 0.878 |
| α-syn_A53T_ vs α-syn_A53T_;Aβ_1-42_ | 5.147 | -0.5225 to 10.82 | ** | 0.0044 |
| α-syn_A53T_ vs α-syn_A53T_;TDP-43 | -4.136 | -8.362 to 0.08978 | * | 0.0491 |
| α-syn_A53T_ vs α-syn_A53T_;Tau_pro-agg_ | -5.485 | -9.130 to -1.841 | ** | 0.0035 |
| α-syn_A53T_ vs α-syn_A53T_;Aβ_1-42_;Tau_pro-agg_ | -3.017 | -6.156 to 0.1228 | * | 0.05 |
| α-syn_A53T_;Aβ_1-42_ vs α-syn_A53T_;Aβ_1-42_;Tau_pro-agg_ | -8.164 | -13.56 to -2.765 | **** | <0.0001 |
| Aβ_1-42_;Tau_pro-agg_ vs α-syn_A53T_;Aβ_1-42_;Tau_pro-agg_ | 1.741 | -2.516 to 5.998 | ns | 0.4199 |
| α-syn_A53T_;Tau_pro-agg_ vs α-syn_A53T_;Aβ_1-42_;Tau_pro-agg_ | 2.469 | -0.7384 to 5.676 | ns | 0.1302 |

# Table S14. The fraction of animals with impaired dopaminergic neurons of *Caenorhabditis elegans* (Figure 4F, day 5 at 23℃).

| **Tukey's multiple comparisons test** | **Mean Diff.** | **95% CI of diff.** | **Summary** | **Adjusted *P* Value** |
| --- | --- | --- | --- | --- |
| WT vs α-syn_A53T_ | -0.5388 | -0.6266 to -0.4511 | **** | <0.0001 |
| WT vs α-syn_A53T_;Aβ_1-42_ | -0.7046 | -0.7924 to -0.6169 | **** | <0.0001 |
| WT vs α-syn_A53T_;TDP-43 | -0.117 | -0.2048 to -0.02930 | ** | 0.0076 |
| WT vs α-syn_A53T_;Tau_pro-agg_ | -0.2054 | -0.2931 to -0.1176 | **** | <0.0001 |
| WT vs α-syn_A53T_;Aβ_1-42_;Tau_pro-agg_ | -0.268 | -0.3557 to -0.1803 | **** | <0.0001 |
| α-syn_A53T_ vs α-syn_A53T_;Aβ_1-42_ | -0.1658 | -0.2535 to -0.07807 | *** | 0.0004 |
| α-syn_A53T_ vs α-syn_A53T_;TDP-43 | 0.4218 | 0.3341 to 0.5095 | **** | <0.0001 |
| α-syn_A53T_ vs α-syn_A53T_;Tau_pro-agg_ | 0.3335 | 0.2457 to 0.4212 | **** | <0.0001 |
| α-syn_A53T_ vs α-syn_A53T_;Aβ_1-42_;Tau_pro-agg_ | 0.2708 | 0.1831 to 0.3586 | **** | <0.0001 |
| α-syn_A53T_;Aβ_1-42_ vs α-syn_A53T_;Aβ_1-42_;Tau_pro-agg_ | 0.4366 | 0.3489 to 0.5244 | **** | <0.0001 |
| α-syn_A53T_;Tau_pro-agg_ vs α-syn_A53T_;Aβ_1-42_;Tau_pro-agg_ | -0.06263 | -0.1504 to 0.02511 | ns | 0.2308 |

# Table S15. The normalization expression level of gene SNCA, MAPT, APP and TARDBP (Figure 5A, day 1 at 23℃)*.*

| **Gene** | **Tukey's multiple comparisons test** | **Mean Diff.** | **95% CI of diff.** | **Summary** | **Adjusted *P* Value** |
| --- | --- | --- | --- | --- | --- |
| **SNCA** | WT vs. α-syn_A53T_ | -1 | -1.670 to -0.3301 | ** | 0.0029 |
|  | WT vs. α-syn_A53T_;Aβ_1-42_ | -5.259 | -5.929 to -4.589 | **** | <0.0001 |
|  | WT vs. α-syn_A53T_;Tau_pro-agg_ | -0.06892 | -0.7388 to 0.6010 | ns | 0.9992 |
|  | WT vs. α-syn_A53T_;TDP-43 | -0.6634 | -1.290 to -0.03676 | * | 0.0356 |
|  | WT vs. α-syn_A53T_;Aβ_1-42_;Tau_pro-agg_ | -0.667 | -1.337 to 0.002860 | ns | 0.0512 |
|  | α-syn_A53T_ vs. α-syn_A53T_;Aβ_1-42_ | -4.259 | -4.929 to -3.589 | **** | <0.0001 |
|  | α-syn_A53T_ vs. α-syn_A53T_;Tau_pro-agg_ | 0.9311 | 0.2612 to 1.601 | ** | 0.0051 |
|  | α-syn_A53T_ vs. α-syn_A53T_;TDP-43 | 0.3366 | -0.2900 to 0.9632 | ns | 0.5083 |
|  | α-syn_A53T_ vs. α-syn_A53T_;Aβ_1-42_;Tau_pro-agg_ | 0.333 | -0.3369 to 1.003 | ns | 0.5844 |
|  | α-syn_A53T_;Aβ_1-42_ vs. α-syn_A53T_;Aβ_1-42_;Tau_pro-agg_ | 4.592 | 3.922 to 5.262 | **** | <0.0001 |
|  | α-syn_A53T_;Tau_pro-agg_ vs. α-syn_A53T_;Aβ_1-42_;Tau_pro-agg_ | -0.5981 | -1.268 to 0.07178 | ns | 0.0919 |
| **MAPT** | WT vs. Tau_pro-agg_ | -1 | -1.073 to -0.9267 | **** | <0.0001 |
|  | WT vs. α-syn_A53T_;Tau_pro-agg_ | -0.6075 | -0.6808 to -0.5342 | **** | <0.0001 |
|  | WT vs. Aβ_1-42_;Tau_pro-agg_ | -0.4692 | -0.5425 to -0.3959 | **** | <0.0001 |
|  | WT vs. α-syn_A53T_;Aβ_1-42_;Tau_pro-agg_ | -1.049 | -1.122 to -0.9758 | **** | <0.0001 |
|  | Tau_pro-agg_ vs. α-syn_A53T_;Tau_pro-agg_ | 0.3925 | 0.3192 to 0.4658 | **** | <0.0001 |
|  | Tau_pro-agg_ vs. Aβ_1-42_;Tau_pro-agg_ | 0.5308 | 0.4575 to 0.6041 | **** | <0.0001 |
|  | Tau_pro-agg_ vs. α-syn_A53T_;Aβ_1-42_;Tau_pro-agg_ | -0.04909 | -0.1224 to 0.02421 | ns | 0.283 |
|  | α-syn_A53T_;Tau_pro-agg_ vs. α-syn_A53T_;Aβ_1-42_;Tau_pro-agg_ | -0.4416 | -0.5149 to -0.3683 | **** | <0.0001 |
|  | Aβ_1-42_;Tau_pro-agg_ vs. α-syn_A53T_;Aβ_1-42_;Tau_pro-agg_ | -0.5799 | -0.6532 to -0.5066 | **** | <0.0001 |
| **APP** | WT vs. Aβ_1-42_ | -1 | -1.568 to -0.4323 | ** | 0.0013 |
|  | WT vs. α-syn_A53T_;Aβ_1-42_ | -1.775 | -2.342 to -1.207 | **** | <0.0001 |
|  | WT vs. Aβ_1-42_;Tau_pro-agg_ | -1.278 | -1.845 to -0.7099 | *** | 0.0002 |
|  | WT vs. α-syn_A53T_;Aβ_1-42_;Tau_pro-agg_ | -2.694 | -3.262 to -2.126 | **** | <0.0001 |
|  | Aβ_1-42_ vs. α-syn_A53T_;Aβ_1-42_ | -0.7746 | -1.342 to -0.2069 | ** | 0.008 |
|  | Aβ_1-42_ vs. Aβ_1-42_;Tau_pro-agg_ | -0.2776 | -0.8453 to 0.2901 | ns | 0.5239 |
|  | Aβ_1-42_ vs. α-syn_A53T_;Aβ_1-42_;Tau_pro-agg_ | -1.694 | -2.262 to -1.126 | **** | <0.0001 |
|  | α-syn_A53T_;Aβ_1-42_ vs. α-syn_A53T_;Aβ_1-42_;Tau_pro-agg_ | -0.9195 | -1.487 to -0.3518 | ** | 0.0024 |
|  | Aβ_1-42_;Tau_pro-agg_ vs. α-syn_A53T_;Aβ_1-42_;Tau_pro-agg_ | -1.417 | -1.984 to -0.8489 | **** | <0.0001 |
| **TARDBP** | WT vs. TDP-43 | -1 | -1.110 to -0.8900 | **** | <0.0001 |
|  | WT vs. α-syn_A53T_;TDP-43 | -0.6325 | -0.7425 to -0.5225 | **** | <0.0001 |
|  | TDP-43 vs. α-syn_A53T_;TDP-43 | 0.3675 | 0.2575 to 0.4775 | **** | <0.0001 |

# Table S16. The relative expression level of gene SNCA, MAPT, APP and TDP-43 (Figure 5B, day 1 at 23℃)*.*

| **Gene** | **Tukey's multiple comparisons test** | **Mean Diff.** | **95% CI of diff.** | **Summary** | **Adjusted *P* Value** |
| --- | --- | --- | --- | --- | --- |
| **SNCA** | WT vs. α-syn_A53T_ | -79.62 | -102.1 to -57.16 | **** | <0.0001 |
|  | WT vs. α-syn_A53T_;Aβ_1-42_ | -277.1 | -306.2 to -247.9 | **** | <0.0001 |
|  | WT vs. α-syn_A53T_;TDP-43 | -55.77 | -84.93 to -26.61 | **** | <0.0001 |
|  | WT vs. α-syn_A53T_;Tau_pro-agg_ | -6.065 | -35.22 to 23.09 | ns | 0.9866 |
|  | WT vs. α-syn_A53T_;Aβ_1-42_;Tau_pro-agg_ | -58.57 | -87.73 to -29.41 | **** | <0.0001 |
|  | α-syn_A53T_ vs. α-syn_A53T_;Aβ_1-42_ | -197.5 | -229.7 to -165.3 | **** | <0.0001 |
|  | α-syn_A53T_ vs. α-syn_A53T_;TDP-43 | 23.86 | -8.336 to 56.05 | ns | 0.2376 |
|  | α-syn_A53T_ vs. α-syn_A53T_;Tau_pro-agg_ | 73.56 | 41.37 to 105.8 | **** | <0.0001 |
|  | α-syn_A53T_ vs. α-syn_A53T_;Aβ_1-42_;Tau_pro-agg_ | 21.06 | -11.13 to 53.25 | ns | 0.3618 |
|  | α-syn_A53T_;Aβ_1-42_ vs. α-syn_A53T_;Aβ_1-42_;Tau_pro-agg_ | 218.5 | 181.3 to 255.7 | **** | <0.0001 |
|  | α-syn_A53T_;Tau_pro-agg_ vs. α-syn_A53T_;Aβ_1-42_;Tau_pro-agg_ | -52.5 | -89.67 to -15.33 | ** | 0.0025 |
| **MAPT** | WT vs. Tau_pro-agg_ | -2.596 | -3.063 to -2.129 | **** | <0.0001 |
|  | WT vs. Aβ_1-42_;Tau_pro-agg_ | -0.9055 | -1.372 to -0.4388 | **** | <0.0001 |
|  | WT vs. α-syn_A53T_;Tau_pro-agg_ | -3.284 | -3.806 to -2.761 | **** | <0.0001 |
|  | WT vs. α-syn_A53T_;Aβ_1-42_;Tau_pro-agg_ | -3.286 | -3.809 to -2.763 | **** | <0.0001 |
|  | Tau_pro-agg_ vs. Aβ_1-42_;Tau_pro-agg_ | 1.69 | 1.113 to 2.268 | **** | <0.0001 |
|  | Tau_pro-agg_ vs. α-syn_A53T_;Tau_pro-agg_ | -0.6877 | -1.311 to -0.06426 | * | 0.0259 |
|  | Tau_pro-agg_ vs. α-syn_A53T_;Aβ_1-42_;Tau_pro-agg_ | -0.6903 | -1.314 to -0.06682 | * | 0.0252 |
|  | Aβ_1-42_;Tau_pro-agg_ vs. α-syn_A53T_;Aβ_1-42_;Tau_pro-agg_ | -2.381 | -3.004 to -1.757 | **** | <0.0001 |
|  | α-syn_A53T_;Tau_pro-agg_ vs. α-syn_A53T_;Aβ_1-42_;Tau_pro-agg_ | -0.00256 | -0.6691 to 0.6640 | ns | >0.9999 |
| **APP** | WT vs. Aβ_1-42_ | -10.65 | -15.12 to -6.187 | **** | <0.0001 |
|  | WT vs. Aβ_1-42_;Tau_pro-agg_ | -11.91 | -16.38 to -7.445 | **** | <0.0001 |
|  | WT vs. α-syn_A53T_;Aβ_1-42_ | -0.3989 | -5.394 to 4.596 | ns | 0.9992 |
|  | WT vs. α-syn_A53T_;Aβ_1-42_;Tau_pro-agg_ | -1.483 | -6.478 to 3.512 | ns | 0.8994 |
|  | Aβ_1-42_ vs. Aβ_1-42_;Tau_pro-agg_ | -1.258 | -6.730 to 4.214 | ns | 0.9576 |
|  | Aβ_1-42_ vs. α-syn_A53T_;Aβ_1-42_ | 10.26 | 4.345 to 16.17 | *** | 0.0004 |
|  | Aβ_1-42_ vs. α-syn_A53T_;Aβ_1-42_;Tau_pro-agg_ | 9.172 | 3.261 to 15.08 | ** | 0.0012 |
|  | Aβ_1-42_;Tau_pro-agg_ vs. α-syn_A53T_;Aβ_1-42_;Tau_pro-agg_ | 10.43 | 4.519 to 16.34 | *** | 0.0003 |
|  | α-syn_A53T_;Aβ_1-42_ vs. α-syn_A53T_;Aβ_1-42_;Tau_pro-agg_ | -1.084 | -7.403 to 5.234 | ns | 0.9853 |
| **TARDBP** | WT vs. TDP-43 | -132.2 | -163.7 to -100.8 | **** | <0.0001 |
|  | WT vs. α-syn_A53T_;TDP-43 | -86.8 | -118.2 to -55.39 | *** | 0.0004 |
|  | TDP-43 vs. α-syn_A53T_;TDP-43 | 45.43 | 14.01 to 76.85 | ** | 0.0094 |

# Table S17. The expression level of monomeric α-syn_A53T_ (Figure 5C, day 1 at 23℃) of *Caenorhabditis elegans*.

| **Tukey's multiple comparisons test** | **Mean Diff.** | **95% CI of diff.** | **Summary** | **Adjusted *P* Value** |
| --- | --- | --- | --- | --- |
| WT vs α-syn_A53T_ | -0.8727 | -1.113 to -0.6320 | **** | <0.0001 |
| WT vs α-syn_A53T_;Aβ_1-42_ | -1.251 | -1.492 to -1.011 | **** | <0.0001 |
| WT vs α-syn_A53T_;TDP-43 | -0.9922 | -1.233 to -0.7515 | **** | <0.0001 |
| WT vs α-syn_A53T_;Tau_pro-agg_ | -0.2179 | -0.459 to 0.0228 | ns | 0.0893 |
| WT vs α-syn_A53T_;Aβ_1-42_;Tau_pro-agg_ | -1.009 | -1.250 to -0.7684 | **** | <0.0001 |
| α-syn_A53T_ vs α-syn_A53T_;Aβ_1-42_ | -0.3787 | -0.6194 to -0.138 | ** | 0.0011 |
| α-syn_A53T_ vs α-syn_A53T_;TDP-43 | -0.1195 | -0.360 to 0.1212 | ns | 0.6221 |
| α-syn_A53T_ vs α-syn_A53T_;Tau_pro-agg_ | 0.6547 | 0.4141 to 0.8954 | **** | <0.0001 |
| α-syn_A53T_ vs α-syn_A53T_;Aβ_1-42_;Tau_pro-agg_ | -0.1364 | -0.377 to 0.1043 | ns | 0.4894 |
| α-syn_A53T_;Aβ_1-42_ vs α-syn_A53T_;Aβ_1-42_;Tau_pro-agg_ | 0.2423 | 0.00165 to 0.483 | * | 0.0479 |
| α-syn_A53T_;Tau_pro-agg_ vs α-syn_A53T_;Aβ_1-42_;Tau_pro-agg_ | -0.7911 | -1.032 to -0.5504 | **** | <0.0001 |

# Table S18. The expression level of aggregation protein α-syn_A53T_ of *Caenorhabditis elegans* (Figure 5D, day 1 at 23℃).

| **Tukey's multiple comparisons test** | **Band 3** | | | | **Band 7** | | | | **Band 8** | | | | **All Bands** | | | |
| --- | --- | --- | --- | --- | --- | --- | --- | --- | --- | --- | --- | --- | --- | --- | --- | --- |
|  | **Mean Diff.** | **95% CI of diff.** | **Summary** | **Adjusted *P* Value** | **Mean Diff.** | **95% CI of diff.** | **Summary** | **Adjusted *P* Value** | **Mean Diff.** | **95% CI of diff.** | **Summary** | **Adjusted *P* Value** | **Mean Diff.** | **95% CI of diff.** | **Summary** | **Adjusted *P* Value** |
| α-syn_A53T_ vs WT | 3.643 | 2.304 to 4.982 | **** | <0.0001 | 1.507 | 0.9210 to 2.093 | **** | <0.0001 | 0.1318 | -0.02405 to 0.2876 | ** | 0.0017 | 8.277 | 6.233 to 10.32 | **** | <0.0001 |
| α-syn_A53T_ vs α-syn_A53T_;Aβ_1-42_ | -4.661 | -6.000 to -3.322 | **** | <0.0001 | -2.393 | -2.979 to -1.807 | **** | <0.0001 | -0.3768 | -0.5226 to -0.2310 | * | 0.0196 | -12.77 | -14.81 to -10.72 | **** | <0.0001 |
| α-syn_A53T_ vs α-syn_A53T_;Tau_pro-agg_ | 3.437 | 2.098 to 4.776 | **** | <0.0001 | 1.356 | 0.7700 to 1.942 | **** | <0.0001 | 0.1289 | -0.02698 to 0.2847 | ** | 0.003 | 7.697 | 5.653 to 9.740 | **** | <0.0001 |
| α-syn_A53T_ vs α-syn_A53T_;TDP-43 | 2.731 | 1.391 to 4.070 | *** | 0.0003 | 1.075 | 0.4895 to 1.661 | *** | 0.0009 | 0.09266 | -0.06317 to 0.2485 | * | 0.0282 | 6.302 | 4.259 to 8.346 | **** | <0.0001 |
| α-syn_A53T_ vs α-syn_A53T_;Aβ_1-42_;Tau_pro-agg_ | 2.778 | 1.439 to 4.117 | *** | 0.0003 | 1.368 | 0.7820 to 1.954 | **** | <0.0001 | 0.1003 | -0.04546 to 0.2461 | ** | 0.0033 | 6.851 | 4.808 to 8.894 | **** | <0.0001 |

# Table S19. The DE-miRNAs of LBD group compared to wild type.

| **miRNA** | **α-syn_A53T_** | ***P*-adjust** | **α-syn_A53T_;Aβ_1-42_** | ***P*-adjust** | **α-syn_A53T_;TDP-43** | ***P*-adjust** | **α-syn_A53T_;Tau_pro-agg_** | **P-adjust** | **α-syn_A53T_;Aβ_1-42_;Tau _pro-agg_** | ***P*-adjust** |
| --- | --- | --- | --- | --- | --- | --- | --- | --- | --- | --- |
| *cel-miR-244-3p* | 1.18 | 6.88E-03 | 1.08 | 4.29E-02 | 0.99 | 6.53E-01 | -0.96 | 5.26E-01 | 0.95 | 5.58E-01 |
| *cel-miR-4816-3p* | -0.03 | 9.32E-01 | -0.09 | 7.80E-01 | -0.36 | 4.86E-01 | -1.36 | 3.57E-08 | -0.90 | 1.11E-03 |
| *cel-miR-79-5p* | 0.54 | 1.74E-02 | 0.18 | 6.24E-01 | 0.23 | 8.85E-01 | 1.24 | 1.26E-02 | 0.34 | 6.79E-01 |
| *cel-miR-71-3p* | 0.09 | 7.83E-01 | -0.21 | 6.24E-01 | 0.14 | 9.22E-01 | -1.32 | 8.31E-05 | 0.11 | 8.74E-01 |
| *cel-miR-794-3p* | 0.05 | 8.98E-01 | -0.13 | 7.80E-01 | 0.02 | 9.83E-01 | -1.61 | 3.62E-03 | -0.17 | 8.58E-01 |
| *cel-miR-354-5p* | 0.97 | 5.92E-01 | 1.24 | 6.16E-01 | 0.71 | 8.87E-01 | 3.41 | 2.89E-02 | -0.44 | 8.75E-01 |
| *cel-miR-260-3p* | 1.04 | 3.23E-03 | 1.20 | 6.06E-03 | -0.17 | 9.50E-01 | 1.26 | 1.44E-02 | -0.86 | 1.89E-01 |
| *cel-miR-1830-3p* | -0.51 | 4.66E-02 | -0.75 | 4.16E-02 | 1.45 | 1.40E-03 | 0.55 | 2.28E-01 | 0.16 | 8.24E-01 |
| *cel-miR-1830-5p* | -0.66 | 5.44E-02 | -0.72 | 1.74E-01 | -0.10 | 9.60E-01 | -1.39 | 5.49E-02 | -1.60 | 2.91E-02 |
| *cel-miR-60-3p* | -0.10 | 7.14E-01 | -0.04 | 9.31E-01 | -0.24 | 7.10E-01 | -1.66 | 1.46E-10 | -0.34 | 4.01E-01 |
| *cel-miR-236-3p* | -0.02 | 9.55E-01 | 0.04 | 9.31E-01 | -0.16 | 8.54E-01 | -1.09 | 1.36E-04 | 0.12 | 8.19E-01 |
| *cel-miR-57-5p* | 0.97 | 2.35E-05 | 1.00 | 2.46E-03 | -0.33 | 6.53E-01 | 1.31 | 3.79E-05 | -0.04 | 9.48E-01 |
| *cel-miR-2216-5p* | -1.20 | 3.34E-02 | -0.69 | 3.96E-01 | 0.42 | 8.73E-01 | -1.30 | 2.28E-01 | -0.12 | 9.39E-01 |
| *cel-miR-35-3p* | 0.76 | 4.66E-02 | 1.18 | 3.21E-02 | -0.71 | 5.08E-01 | 0.44 | 5.14E-01 | -1.70 | 2.87E-03 |
| *cel-miR-36-3p* | 0.78 | 2.82E-02 | 1.05 | 3.89E-02 | -0.27 | 8.48E-01 | 0.44 | 4.83E-01 | -0.84 | 1.58E-01 |
| *cel-miR-37-3p* | 0.74 | 2.81E-02 | 1.15 | 1.74E-02 | -0.61 | 5.08E-01 | 0.57 | 2.87E-01 | -1.45 | 3.96E-03 |
| *cel-miR-38-3p* | 1.03 | 4.58E-04 | 2.35 | 7.83E-11 | -0.58 | 4.86E-01 | 0.75 | 8.55E-02 | -0.91 | 5.23E-02 |
| *cel-miR-39-3p* | 0.76 | 5.44E-02 | 1.03 | 6.87E-02 | -0.23 | 9.14E-01 | 1.20 | 3.51E-02 | -1.12 | 8.13E-02 |
| *cel-miR-40-3p* | 0.53 | 1.92E-01 | 0.86 | 1.22E-01 | -0.98 | 2.32E-01 | 0.38 | 5.74E-01 | -1.89 | 4.08E-04 |
| *cel-miR-41-3p* | 1.05 | 9.99E-03 | 1.29 | 2.19E-02 | -0.98 | 2.49E-01 | -0.85 | 1.59E-01 | -1.08 | 9.06E-02 |
| *cel-miR-8212-3p* | -0.21 | 7.28E-01 | -0.37 | 6.24E-01 | 1.91 | 5.08E-01 | 3.58 | 1.80E-02 | 2.10 | 3.03E-01 |
| *cel-miR-355-5p* | 1.16 | 1.12E-06 | 1.90 | 8.87E-10 | -0.03 | 9.83E-01 | -0.07 | 8.96E-01 | -0.98 | 1.33E-02 |
| *cel-miR-5594-3p* | -0.95 | 4.23E-03 | -0.52 | 2.52E-01 | -0.71 | 5.12E-01 | 2.04 | 8.65E-05 | -0.24 | 8.07E-01 |
| *cel-miR-45-3p* | 0.49 | 5.29E-03 | 0.15 | 6.24E-01 | -0.65 | 5.67E-02 | -0.93 | 8.49E-05 | -1.03 | 4.55E-05 |
| *cel-miR-42-5p* | 1.33 | 7.59E-11 | 0.94 | 7.95E-04 | 0.32 | 6.66E-01 | 0.45 | 2.62E-01 | -0.10 | 8.74E-01 |
| *cel-miR-42-3p* | 1.09 | 8.87E-04 | 1.10 | 2.22E-02 | -1.15 | 8.17E-02 | 0.01 | 9.92E-01 | -1.81 | 1.61E-04 |
| *cel-miR-43-5p* | 2.16 | 7.03E-04 | 1.65 | 4.01E-02 | 0.71 | 8.07E-01 | 0.79 | 6.03E-01 | 0.79 | 6.79E-01 |
| *cel-miR-43-3p* | 0.94 | 7.62E-04 | 1.10 | 1.76E-03 | -0.86 | 9.97E-02 | 0.19 | 7.07E-01 | -1.09 | 8.04E-03 |
| *cel-miR-44-5p* | 1.49 | 1.76E-18 | 1.06 | 7.44E-06 | -0.31 | 5.41E-01 | 1.07 | 1.72E-05 | -0.68 | 2.16E-02 |
| *cel-miR-44-3p* | 0.49 | 5.29E-03 | 0.15 | 6.24E-01 | -0.65 | 5.67E-02 | -0.93 | 8.49E-05 | -1.03 | 4.55E-05 |
| *cel-miR-77-5p* | -0.01 | 9.92E-01 | 0.22 | 7.94E-01 | 0.28 | 8.98E-01 | 1.48 | 1.81E-02 | 0.43 | 6.77E-01 |
| *cel-miR-77-3p* | -0.60 | 3.05E-03 | -0.38 | 2.48E-01 | -0.38 | 5.08E-01 | -1.12 | 4.32E-05 | -0.71 | 2.73E-02 |
| *cel-miR-2217b-2-3p* | -1.19 | 2.57E-02 | -0.45 | 5.05E-01 | -0.40 | 9.00E-01 | -1.09 | 3.47E-01 | -0.04 | 9.69E-01 |
| *cel-miR-64-3p* | -0.14 | 6.67E-01 | -0.42 | 2.38E-01 | 0.10 | 9.60E-01 | -1.10 | 2.12E-02 | 0.27 | 7.21E-01 |
| *cel-miR-65-5p* | -0.41 | 3.84E-02 | -0.61 | 3.26E-02 | -0.16 | 8.35E-01 | -1.14 | 1.24E-05 | -0.50 | 1.37E-01 |
| *cel-miR-65-3p* | -0.37 | 2.56E-01 | -0.56 | 1.39E-01 | -0.03 | 9.83E-01 | -1.02 | 2.52E-02 | -0.22 | 7.52E-01 |
| *cel-miR-76-3p* | 0.07 | 8.34E-01 | 0.72 | 2.23E-02 | -0.18 | 8.48E-01 | 1.25 | 6.64E-05 | 0.64 | 9.77E-02 |
| *cel-miR-4813-5p* | 0.39 | 9.84E-02 | 0.23 | 5.45E-01 | 0.25 | 7.97E-01 | 2.36 | 1.73E-14 | -0.45 | 3.38E-01 |
| *cel-miR-4813-3p* | 0.16 | 6.61E-01 | 0.31 | 4.65E-01 | -0.86 | 8.14E-02 | 1.21 | 3.50E-04 | -0.61 | 1.57E-01 |
| *cel-miR-5549-3p* | -1.05 | 3.61E-02 | -0.31 | 6.57E-01 | -1.06 | 3.20E-01 | -1.70 | 1.26E-02 | -1.32 | 8.13E-02 |
| *cel-miR-2214-5p* | 0.08 | 8.28E-01 | 0.08 | 8.98E-01 | 0.58 | 3.99E-01 | 2.74 | 6.33E-16 | 0.59 | 2.08E-01 |
| *cel-miR-2214-3p* | 1.36 | 1.37E-02 | 1.37 | 4.41E-02 | 0.70 | 6.97E-01 | 0.99 | 2.92E-01 | 0.49 | 7.24E-01 |
| *cel-miR-231-3p* | 0.66 | 2.82E-02 | 0.53 | 2.49E-01 | 0.01 | 9.84E-01 | 1.22 | 6.03E-03 | 0.26 | 7.36E-01 |
| *cel-miR-231-5p* | 1.05 | 8.69E-09 | 0.67 | 7.25E-03 | -0.37 | 5.13E-01 | 1.71 | 4.93E-10 | -0.34 | 4.55E-01 |
| *cel-miR-1020-3p* | -0.18 | 4.42E-01 | -0.37 | 1.96E-01 | -0.32 | 5.43E-01 | -1.58 | 7.73E-10 | -0.80 | 8.12E-03 |
| *cel-miR-1832a-5p* | 0.25 | 5.21E-01 | -0.47 | 3.09E-01 | -0.14 | 9.60E-01 | 3.26 | 4.17E-12 | -0.11 | 9.08E-01 |
| *cel-miR-1832a-3p* | -0.54 | 1.62E-01 | -0.58 | 2.84E-01 | -0.66 | 5.13E-01 | 1.95 | 9.29E-05 | -1.01 | 1.26E-01 |
| *cel-miR-86-3p* | 0.76 | 4.49E-05 | 0.55 | 4.72E-02 | 0.01 | 9.84E-01 | 1.85 | 4.17E-12 | 0.66 | 5.13E-02 |
| *cel-miR-4814-3p* | 0.28 | 4.67E-01 | 0.01 | 9.86E-01 | 1.56 | 5.43E-01 | 3.73 | 3.98E-03 | 2.68 | 8.24E-02 |
| *cel-miR-2208a-5p* | -0.12 | 8.82E-01 | 0.75 | 3.05E-01 | -2.19 | 2.47E-03 | 0.98 | 1.54E-01 | -1.62 | 2.73E-02 |
| *cel-miR-2208b-5p* | 0.20 | 6.11E-01 | -0.20 | 6.97E-01 | -0.39 | 8.03E-01 | 1.23 | 4.09E-02 | -0.04 | 9.61E-01 |
| *cel-miR-4937-5p* | -0.96 | 9.13E-04 | -0.93 | 1.91E-02 | 0.23 | 8.54E-01 | 0.71 | 1.53E-01 | -1.16 | 2.59E-02 |
| *cel-miR-242-5p* | 0.90 | 1.11E-03 | 0.25 | 5.98E-01 | -0.11 | 9.60E-01 | 1.31 | 5.67E-04 | 0.13 | 8.58E-01 |
| *cel-miR-243-3p* | 0.26 | 2.32E-01 | -0.11 | 7.58E-01 | 0.06 | 9.60E-01 | 1.40 | 4.39E-08 | 0.24 | 5.62E-01 |
| *cel-miR-8191-5p* | -1.74 | 1.99E-01 | -3.70 | 4.41E-02 | 1.53 | 6.53E-01 | 1.36 | 5.18E-01 | 1.34 | 5.77E-01 |
| *cel-miR-228-5p* | 0.20 | 2.52E-01 | -0.03 | 9.31E-01 | -0.07 | 9.40E-01 | 1.59 | 4.54E-16 | -0.11 | 7.49E-01 |
| *cel-miR-790-5p* | 0.14 | 7.28E-01 | 0.76 | 8.63E-02 | -0.61 | 4.78E-01 | 1.94 | 9.67E-07 | -0.36 | 5.77E-01 |
| *cel-miR-790-3p* | 0.28 | 4.48E-01 | 0.37 | 4.77E-01 | 0.22 | 8.88E-01 | 1.36 | 3.62E-03 | 0.45 | 5.52E-01 |
| *cel-miR-8202-5p* | -0.48 | 6.15E-01 | -1.97 | 1.14E-01 | -1.05 | 5.08E-01 | -1.45 | 1.27E-01 | -2.52 | 9.05E-03 |
| *cel-miR-58b-5p* | -1.72 | 1.17E-02 | -1.67 | 1.21E-01 | 2.12 | 3.99E-01 | 2.29 | 1.41E-01 | 1.44 | 5.09E-01 |
| *cel-miR-58c-3p* | -1.21 | 1.29E-04 | -2.65 | 1.45E-06 | 0.53 | 9.24E-01 | 2.99 | 3.51E-02 | 0.94 | 6.85E-01 |
| *cel-miR-1820-5p* | 0.27 | 2.35E-01 | 0.27 | 4.40E-01 | 0.07 | 9.60E-01 | 1.47 | 2.94E-08 | 0.14 | 7.54E-01 |
| *cel-miR-2210-3p* | 0.23 | 5.58E-01 | -1.18 | 3.62E-03 | -1.25 | 2.24E-01 | 1.37 | 3.81E-02 | 1.18 | 1.23E-01 |
| *cel-miR-246-5p* | -0.33 | 4.37E-01 | -0.10 | 9.05E-01 | 0.65 | 5.40E-01 | -0.65 | 2.99E-01 | 1.46 | 1.45E-02 |
| *cel-miR-51-3p* | -0.79 | 2.05E-03 | -0.61 | 1.03E-01 | 0.41 | 5.79E-01 | -1.50 | 1.71E-05 | -0.56 | 2.28E-01 |
| *cel-miR-51-5p* | -0.51 | 1.51E-03 | 0.01 | 9.80E-01 | -0.39 | 3.19E-01 | -0.58 | 1.22E-02 | -1.13 | 8.66E-07 |
| *cel-miR-59-5p* | 1.85 | 5.19E-03 | 1.35 | 1.04E-01 | -0.27 | 9.60E-01 | -1.53 | 3.98E-01 | -0.59 | 7.94E-01 |
| *cel-miR-52-3p* | 1.36 | 7.34E-06 | 0.79 | 8.29E-02 | 0.09 | 9.60E-01 | 0.10 | 8.96E-01 | 0.30 | 7.26E-01 |
| *cel-miR-798-3p* | -0.77 | 3.95E-01 | -2.55 | 2.24E-02 | -0.25 | 9.61E-01 | -2.75 | 2.32E-01 | -1.52 | 5.59E-01 |
| *cel-miR-2217b-3-3p* | -1.23 | 3.12E-02 | -0.52 | 5.00E-01 | -0.05 | 9.83E-01 | -0.98 | 4.83E-01 | 0.28 | 8.74E-01 |
| *cel-miR-78-3p* | -1.01 | 2.25E-03 | -3.57 | 3.16E-18 | 0.29 | 8.18E-01 | -0.66 | 2.30E-01 | -1.90 | 1.52E-04 |
| *cel-miR-2217b-4-3p* | -1.23 | 3.12E-02 | -0.52 | 5.00E-01 | -0.05 | 9.83E-01 | -0.98 | 4.83E-01 | 0.28 | 8.74E-01 |
| *cel-miR-8198-3p* | -0.91 | 3.12E-02 | -3.57 | 3.82E-07 | 0.63 | 8.03E-01 | 0.59 | 6.68E-01 | -1.97 | 1.35E-01 |
| *cel-miR-789-2-5p* | -0.93 | 4.37E-04 | -1.41 | 7.73E-05 | -0.19 | 9.19E-01 | 0.58 | 2.59E-01 | 0.50 | 4.52E-01 |
| *cel-miR-2219-3p* | -0.06 | 9.64E-01 | -0.17 | 9.11E-01 | -1.93 | 1.10E-01 | -0.82 | 4.57E-01 | -2.56 | 1.48E-02 |
| *cel-miR-792-3p* | 0.08 | 8.61E-01 | 0.40 | 3.78E-01 | -0.32 | 9.24E-01 | 1.97 | 2.11E-02 | -0.25 | 8.74E-01 |
| *cel-miR-1821-3p* | 0.73 | 5.35E-03 | 0.86 | 1.76E-02 | -0.49 | 5.40E-01 | 1.20 | 2.40E-03 | -0.09 | 9.08E-01 |
| *cel-miR-8200-3p* | -2.46 | 2.20E-02 | -3.46 | 3.89E-02 | -1.52 | 6.53E-01 | -0.92 | 6.96E-01 | -0.61 | 8.41E-01 |
| *cel-miR-4922-2-3p* | 1.17 | 1.79E-02 | 0.19 | 8.37E-01 | -0.70 | 8.03E-01 | -0.15 | 9.06E-01 | -0.20 | 9.08E-01 |
| *cel-miR-4922-1-3p* | 1.17 | 1.74E-02 | 0.22 | 8.13E-01 | -0.70 | 8.03E-01 | -0.15 | 9.06E-01 | -0.20 | 9.08E-01 |
| *cel-miR-253-3p* | -1.51 | 1.84E-08 | -0.61 | 1.60E-01 | 0.29 | 8.03E-01 | -0.22 | 6.96E-01 | -0.43 | 4.87E-01 |
| *cel-miR-70-5p* | 0.05 | 8.89E-01 | -0.65 | 5.56E-02 | 0.03 | 9.83E-01 | -0.16 | 8.45E-01 | 2.48 | 4.36E-06 |
| *cel-miR-357-3p* | -0.31 | 5.74E-02 | -0.14 | 5.98E-01 | -0.06 | 9.60E-01 | -1.16 | 4.53E-07 | -0.03 | 9.54E-01 |
| *cel-miR-250-3p* | -0.21 | 2.43E-01 | 0.28 | 2.84E-01 | -0.10 | 9.05E-01 | -1.04 | 1.02E-06 | 0.41 | 1.37E-01 |
| *cel-miR-250-5p* | -0.40 | 1.12E-01 | -0.47 | 1.96E-01 | 0.01 | 9.84E-01 | -1.86 | 1.15E-08 | -0.18 | 7.49E-01 |
| *cel-miR-241-3p* | -0.16 | 3.99E-01 | -0.58 | 8.73E-03 | -0.22 | 6.79E-01 | -1.52 | 1.22E-11 | -0.38 | 2.07E-01 |
| *cel-miR-800-3p* | -0.60 | 4.72E-02 | -1.40 | 7.72E-04 | -0.84 | 5.79E-01 | -0.28 | 8.11E-01 | -0.46 | 7.27E-01 |
| *cel-miR-796-5p* | 0.93 | 9.84E-02 | 1.33 | 3.89E-02 | -1.31 | 1.17E-01 | 2.02 | 1.90E-04 | -0.55 | 5.52E-01 |
| *cel-miR-4807-3p* | 1.59 | 2.92E-05 | 2.33 | 5.39E-08 | -1.08 | 1.28E-01 | 1.56 | 8.53E-04 | 1.00 | 8.13E-02 |
| *cel-miR-4808-3p* | 0.24 | 6.15E-01 | 0.66 | 1.54E-01 | -1.47 | 1.05E-02 | -0.27 | 6.86E-01 | 0.31 | 7.08E-01 |
| *cel-miR-4809-3p* | 0.60 | 5.65E-01 | 1.90 | 3.99E-02 | -2.43 | 7.80E-02 | 0.18 | 8.96E-01 | -0.85 | 5.62E-01 |
| *cel-miR-2220-3p* | 0.80 | 3.84E-02 | 1.32 | 1.72E-03 | -1.61 | 1.40E-03 | -0.07 | 9.04E-01 | -0.25 | 7.28E-01 |
| *cel-miR-1018-3p* | 1.33 | 1.20E-04 | 1.56 | 1.03E-04 | -0.63 | 5.08E-01 | 1.99 | 1.38E-05 | 0.97 | 9.12E-02 |
| *cel-miR-8196a-5p* | -0.63 | 3.40E-01 | 0.42 | 5.47E-01 | -0.77 | 5.03E-01 | -3.64 | 1.10E-06 | -3.00 | 5.65E-06 |
| *cel-miR-74-5p* | 0.47 | 5.80E-02 | 0.10 | 8.28E-01 | 0.09 | 9.60E-01 | 1.26 | 1.97E-04 | 0.30 | 5.71E-01 |
| *cel-miR-359-3p* | -0.60 | 4.24E-02 | -0.33 | 5.03E-01 | -0.23 | 8.48E-01 | 1.13 | 5.09E-03 | -0.33 | 5.94E-01 |
| *cel-miR-785-3p* | -0.48 | 3.97E-02 | -0.19 | 6.24E-01 | -0.03 | 9.78E-01 | 1.01 | 1.76E-03 | 0.36 | 4.70E-01 |
| *cel-miR-249-3p* | 0.12 | 7.28E-01 | 0.53 | 1.54E-01 | 0.08 | 9.60E-01 | 1.70 | 1.88E-06 | 0.35 | 5.58E-01 |
| *cel-miR-247-3p* | 1.04 | 4.70E-03 | 1.71 | 7.04E-04 | -0.47 | 7.10E-01 | -0.53 | 4.60E-01 | 1.20 | 7.24E-02 |
| *cel-miR-797-5p* | 1.73 | 3.14E-07 | 2.09 | 9.69E-07 | -0.51 | 7.10E-01 | -0.68 | 3.76E-01 | 1.12 | 1.34E-01 |
| *cel-miR-797-3p* | 1.60 | 8.18E-08 | 1.31 | 4.09E-04 | 1.87 | 3.86E-01 | 2.13 | 9.88E-02 | 2.91 | 2.73E-02 |
| *cel-miR-230-3p* | 0.65 | 4.77E-03 | 0.62 | 6.56E-02 | -0.08 | 9.60E-01 | -1.08 | 7.47E-04 | -0.58 | 1.48E-01 |
| *cel-miR-1022-5p* | 0.63 | 1.26E-03 | 0.73 | 9.10E-03 | 0.35 | 5.79E-01 | 1.60 | 3.82E-08 | 0.80 | 2.49E-02 |
| *cel-miR-240-5p* | 0.25 | 2.51E-01 | 0.21 | 5.03E-01 | 0.19 | 8.03E-01 | 1.23 | 1.13E-05 | 0.92 | 3.96E-03 |
| *cel-miR-784-3p* | -1.06 | 1.12E-03 | -0.84 | 4.79E-02 | -0.18 | 9.60E-01 | 0.12 | 9.06E-01 | -0.80 | 4.96E-01 |
| *cel-miR-784-5p* | -0.67 | 4.77E-05 | -0.35 | 1.26E-01 | -0.21 | 7.07E-01 | 1.06 | 7.51E-06 | -0.47 | 1.25E-01 |
| *cel-miR-237-3p* | -0.19 | 5.99E-01 | -0.89 | 2.22E-02 | 0.20 | 8.85E-01 | -1.09 | 1.26E-02 | 0.08 | 9.08E-01 |
| *cel-miR-788-5p* | 1.23 | 6.71E-03 | 0.94 | 1.76E-01 | -1.13 | 3.20E-01 | -1.74 | 1.02E-02 | -0.01 | 9.91E-01 |
| *cel-miR-799-3p* | -0.69 | 2.81E-02 | -1.36 | 3.59E-03 | 0.53 | 8.03E-01 | 0.52 | 6.13E-01 | 0.33 | 8.09E-01 |
| *cel-miR-49-5p* | 0.04 | 8.78E-01 | -0.26 | 3.60E-01 | -0.86 | 2.57E-03 | 1.06 | 7.51E-06 | -1.34 | 1.39E-07 |
| *cel-miR-49-3p* | -0.07 | 7.28E-01 | -0.17 | 4.78E-01 | -0.43 | 1.34E-01 | 1.11 | 4.42E-10 | -0.57 | 8.04E-03 |
| *cel-miR-251-5p* | -0.35 | 2.77E-01 | -0.27 | 5.21E-01 | 0.05 | 9.78E-01 | 1.15 | 1.15E-02 | -1.09 | 4.10E-02 |
| *cel-miR-239b-5p* | 1.70 | 5.30E-05 | 0.73 | 2.38E-01 | -0.92 | 4.86E-01 | -0.32 | 7.26E-01 | 1.69 | 2.12E-02 |
| *cel-miR-239a-5p* | 0.87 | 5.99E-04 | 0.50 | 1.96E-01 | -0.50 | 4.86E-01 | -1.14 | 1.38E-03 | 0.65 | 1.37E-01 |
| *cel-miR-62-3p* | 0.36 | 8.17E-02 | 0.28 | 3.96E-01 | -0.51 | 2.49E-01 | -1.41 | 6.20E-08 | -0.58 | 8.13E-02 |
| *cel-miR-54-5p* | 0.42 | 1.08E-02 | 0.15 | 5.98E-01 | 0.16 | 8.03E-01 | 1.31 | 7.56E-09 | 0.45 | 1.34E-01 |
| *cel-miR-392-3p* | 2.06 | 3.83E-02 | 1.48 | 2.84E-01 | -0.44 | 9.60E-01 | -0.95 | 7.26E-01 | 0.29 | 9.11E-01 |
| *cel-miR-8196b-5p* | -0.33 | 6.61E-01 | 0.53 | 4.77E-01 | -0.63 | 6.26E-01 | -2.93 | 3.12E-05 | -2.45 | 3.40E-04 |
| *cel-let-7-3p* | 1.84 | 5.62E-04 | 1.47 | 6.16E-02 | -0.05 | 9.83E-01 | -1.81 | 6.53E-02 | 1.08 | 3.90E-01 |
| *cel-miR-1829c-5p* | -1.17 | 1.79E-08 | -0.60 | 4.41E-02 | 0.42 | 5.13E-01 | -0.56 | 1.39E-01 | -0.23 | 6.77E-01 |
| *cel-miR-4812-3p* | -0.85 | 2.32E-02 | -0.95 | 4.41E-02 | -0.61 | 6.80E-01 | -2.87 | 1.38E-03 | -1.51 | 8.13E-02 |
| *cel-miR-4812-5p* | -0.89 | 4.37E-04 | -0.47 | 1.74E-01 | 0.13 | 9.60E-01 | -1.42 | 7.07E-04 | -0.25 | 7.15E-01 |
| *cel-miR-1829a-5p* | -1.41 | 4.19E-40 | -1.30 | 4.32E-22 | 0.03 | 9.60E-01 | -0.15 | 5.26E-01 | -0.88 | 4.04E-06 |
| *cel-miR-2212-5p* | -0.55 | 3.56E-02 | -0.44 | 2.48E-01 | 0.41 | 7.22E-01 | 1.82 | 1.36E-04 | 0.66 | 3.46E-01 |
| *cel-miR-1819-3p* | -0.69 | 7.54E-03 | -0.55 | 1.62E-01 | 0.99 | 3.10E-01 | 3.42 | 6.47E-11 | 1.66 | 8.27E-03 |
| *cel-miR-2217b-1-3p* | -1.23 | 3.12E-02 | -0.52 | 5.00E-01 | -0.05 | 9.83E-01 | -0.98 | 4.83E-01 | 0.28 | 8.74E-01 |

# Table S20. The DE-miRNAs of WT, α-syn_A53T_;TDP-43, α-syn_A53T_;Tau_pro-agg_, and α-syn_A53T_;Aβ_1-42_;Tau_pro-agg_ compared with α-syn_A53T_.

| **miRNA** | **WT** | ***P*-adjust** | **α-syn_A53T_;TDP-43** | ***P*-adjust** | **α-syn_A53T_;Tau_pro-agg_** | ***P*-adjust** | **α-syn_A53T_;Aβ_1-42_;Tau_pro-agg_** | ***P*-adjust** |
| --- | --- | --- | --- | --- | --- | --- | --- | --- |
| *cel-miR-244-3p* | -1.18 | 6.88E-03 | -0.19 | 9.41E-01 | -2.14 | 1.36E-01 | -0.23 | 8.95E-01 |
| *cel-miR-4816-5p* | -0.43 | 6.71E-03 | -0.89 | 4.04E-03 | -0.45 | 1.92E-01 | -1.38 | 1.20E-06 |
| *cel-miR-4816-3p* | 0.03 | 9.32E-01 | -0.34 | 4.93E-01 | -1.34 | 1.63E-05 | -0.87 | 1.29E-02 |
| *cel-miR-245-3p* | 0.39 | 5.61E-02 | 0.84 | 4.01E-02 | 1.31 | 1.64E-04 | 0.57 | 2.01E-01 |
| *cel-miR-71-3p* | -0.09 | 7.83E-01 | 0.05 | 9.52E-01 | -1.41 | 8.48E-04 | 0.01 | 9.82E-01 |
| *cel-miR-1817-5p* | 0.92 | 8.93E-05 | 0.94 | 6.97E-02 | 0.20 | 7.63E-01 | 1.05 | 3.64E-02 |
| *cel-miR-795-5p* | 0.58 | 1.12E-03 | 1.52 | 1.12E-05 | 1.14 | 6.36E-04 | 0.97 | 9.54E-03 |
| *cel-miR-794-3p* | -0.05 | 8.98E-01 | -0.03 | 9.89E-01 | -1.66 | 9.16E-03 | -0.23 | 8.11E-01 |
| *cel-miR-260-3p* | -1.04 | 3.23E-03 | -1.21 | 1.07E-01 | 0.23 | 8.14E-01 | -1.90 | 6.04E-03 |
| *cel-miR-1830-3p* | 0.51 | 4.66E-02 | 1.96 | 5.65E-05 | 1.06 | 3.34E-02 | 0.67 | 2.68E-01 |
| *cel-miR-1822-3p* | 0.26 | 2.20E-01 | 0.52 | 2.71E-01 | 1.22 | 3.52E-04 | 0.22 | 6.87E-01 |
| *cel-miR-60-3p* | 0.10 | 7.14E-01 | -0.13 | 8.47E-01 | -1.56 | 2.15E-06 | -0.24 | 6.40E-01 |
| *cel-miR-236-3p* | 0.02 | 9.55E-01 | -0.14 | 8.47E-01 | -1.07 | 3.68E-03 | 0.14 | 8.11E-01 |
| *cel-miR-57-3p* | -0.94 | 3.44E-06 | -1.34 | 1.62E-03 | -1.58 | 5.82E-05 | -1.04 | 1.92E-02 |
| *cel-miR-57-5p* | -0.97 | 2.35E-05 | -1.30 | 3.19E-03 | 0.34 | 4.99E-01 | -1.01 | 3.09E-02 |
| *cel-miR-2216-5p* | 1.20 | 3.34E-02 | 1.62 | 2.26E-01 | -0.10 | 9.69E-01 | 1.08 | 4.44E-01 |
| *cel-miR-35-5p* | -0.67 | 8.27E-02 | -0.62 | 5.04E-01 | -1.60 | 9.16E-03 | -1.33 | 5.43E-02 |
| *cel-miR-35-3p* | -0.76 | 4.66E-02 | -1.47 | 5.42E-02 | -0.32 | 7.40E-01 | -2.47 | 1.44E-04 |
| *cel-miR-36-3p* | -0.78 | 2.82E-02 | -1.05 | 1.76E-01 | -0.34 | 6.88E-01 | -1.62 | 1.46E-02 |
| *cel-miR-37-3p* | -0.74 | 2.81E-02 | -1.35 | 4.14E-02 | -0.17 | 8.45E-01 | -2.19 | 1.44E-04 |
| *cel-miR-38-3p* | -1.03 | 4.58E-04 | -1.61 | 3.19E-03 | -0.28 | 6.96E-01 | -1.94 | 1.44E-04 |
| *cel-miR-39-3p* | -0.76 | 5.44E-02 | -1.00 | 2.71E-01 | 0.44 | 6.34E-01 | -1.88 | 1.11E-02 |
| *cel-miR-40-3p* | -0.53 | 1.92E-01 | -1.51 | 4.01E-02 | -0.15 | 8.75E-01 | -2.42 | 1.44E-04 |
| *cel-miR-41-3p* | -1.05 | 9.99E-03 | -2.03 | 5.48E-03 | -1.90 | 5.03E-03 | -2.13 | 3.21E-03 |
| *cel-miR-8212-3p* | 0.21 | 7.28E-01 | 2.13 | 3.27E-01 | 3.79 | 1.74E-02 | 2.31 | 2.46E-01 |
| *cel-miR-355-5p* | -1.16 | 1.12E-06 | -1.19 | 1.34E-02 | -1.24 | 4.83E-03 | -2.14 | 3.50E-07 |
| *cel-miR-5594-3p* | 0.95 | 4.23E-03 | 0.24 | 8.47E-01 | 2.99 | 9.18E-07 | 0.71 | 4.14E-01 |
| *cel-miR-45-3p* | -0.49 | 5.29E-03 | -1.14 | 2.75E-04 | -1.42 | 9.18E-07 | -1.52 | 2.67E-07 |
| *cel-miR-45-5p* | -0.89 | 3.14E-07 | -0.87 | 1.55E-02 | -1.08 | 6.75E-04 | -0.73 | 5.43E-02 |
| *cel-miR-42-5p* | -1.33 | 7.59E-11 | -1.00 | 3.16E-02 | -0.88 | 4.60E-02 | -1.43 | 7.79E-04 |
| *cel-miR-42-3p* | -1.09 | 8.87E-04 | -2.24 | 1.79E-04 | -1.08 | 9.22E-02 | -2.90 | 2.67E-07 |
| *cel-miR-43-5p* | -2.16 | 7.03E-04 | -1.46 | 4.76E-01 | -1.37 | 3.92E-01 | -1.37 | 4.44E-01 |
| *cel-miR-43-3p* | -0.94 | 7.62E-04 | -1.80 | 1.79E-04 | -0.75 | 1.72E-01 | -2.03 | 1.07E-05 |
| *cel-miR-44-5p* | -1.49 | 1.76E-18 | -1.80 | 8.42E-09 | -0.42 | 2.80E-01 | -2.17 | 6.84E-13 |
| *cel-miR-44-3p* | -0.49 | 5.29E-03 | -1.14 | 2.75E-04 | -1.42 | 9.18E-07 | -1.52 | 2.67E-07 |
| *cel-miR-2217b-2-3p* | 1.19 | 2.57E-02 | 0.79 | 6.42E-01 | 0.10 | 9.69E-01 | 1.14 | 4.14E-01 |
| *cel-miR-76-3p* | -0.07 | 8.34E-01 | -0.25 | 7.16E-01 | 1.18 | 2.95E-03 | 0.58 | 2.54E-01 |
| *cel-miR-4936-3p* | 0.73 | 8.98E-03 | 1.19 | 6.75E-02 | 1.41 | 1.33E-02 | 0.98 | 1.48E-01 |
| *cel-miR-4813-5p* | -0.39 | 9.84E-02 | -0.14 | 8.51E-01 | 1.97 | 3.22E-07 | -0.84 | 7.71E-02 |
| *cel-miR-4813-3p* | -0.16 | 6.61E-01 | -1.02 | 4.46E-02 | 1.05 | 1.98E-02 | -0.77 | 1.49E-01 |
| *cel-miR-5549-3p* | 1.05 | 3.61E-02 | -0.01 | 9.98E-01 | -0.65 | 5.43E-01 | -0.27 | 8.21E-01 |
| *cel-miR-2214-5p* | -0.08 | 8.28E-01 | 0.50 | 4.78E-01 | 2.66 | 6.54E-10 | 0.51 | 4.14E-01 |
| *cel-miR-2214-3p* | -1.36 | 1.37E-02 | -0.67 | 6.97E-01 | -0.37 | 8.13E-01 | -0.87 | 5.50E-01 |
| *cel-miR-231-5p* | -1.05 | 8.69E-09 | -1.42 | 1.21E-04 | 0.66 | 9.45E-02 | -1.39 | 1.44E-04 |
| *cel-miR-238-5p* | -0.56 | 6.05E-03 | -0.22 | 7.85E-01 | -1.31 | 2.60E-03 | -0.24 | 7.16E-01 |
| *cel-miR-90-5p* | -0.90 | 1.17E-06 | -1.05 | 6.10E-03 | -1.38 | 6.73E-05 | -0.95 | 1.49E-02 |
| *cel-miR-1020-3p* | 0.18 | 4.42E-01 | -0.14 | 8.26E-01 | -1.40 | 1.63E-05 | -0.62 | 1.26E-01 |
| *cel-miR-1832a-5p* | -0.25 | 5.21E-01 | -0.39 | 7.09E-01 | 3.01 | 6.81E-08 | -0.36 | 7.06E-01 |
| *cel-miR-1832a-3p* | 0.54 | 1.62E-01 | -0.12 | 9.41E-01 | 2.49 | 5.11E-05 | -0.47 | 6.20E-01 |
| *cel-miR-86-3p* | -0.76 | 4.49E-05 | -0.76 | 6.67E-02 | 1.09 | 1.84E-03 | -0.10 | 8.38E-01 |
| *cel-miR-4814-3p* | -0.28 | 4.67E-01 | 1.28 | 5.47E-01 | 3.45 | 1.22E-02 | 2.40 | 1.48E-01 |
| *cel-miR-2208a-5p* | 0.12 | 8.82E-01 | -2.07 | 2.99E-02 | 1.11 | 2.72E-01 | -1.50 | 1.49E-01 |
| *cel-miR-4937-5p* | 0.96 | 9.13E-04 | 1.19 | 5.42E-02 | 1.67 | 1.76E-03 | -0.20 | 8.11E-01 |
| *cel-miR-243-3p* | -0.26 | 2.32E-01 | -0.20 | 7.29E-01 | 1.14 | 6.05E-04 | -0.02 | 9.66E-01 |
| *cel-miR-228-5p* | -0.20 | 2.52E-01 | -0.27 | 5.04E-01 | 1.39 | 1.70E-08 | -0.31 | 3.72E-01 |
| *cel-miR-228-3p* | -0.81 | 1.09E-06 | -1.05 | 2.96E-03 | -0.81 | 1.65E-02 | -0.72 | 5.64E-02 |
| *cel-miR-790-5p* | -0.14 | 7.28E-01 | -0.75 | 2.94E-01 | 1.80 | 4.53E-04 | -0.50 | 4.91E-01 |
| *cel-miR-58b-5p* | 1.72 | 1.17E-02 | 3.84 | 2.88E-02 | 4.01 | 1.17E-02 | 3.16 | 8.69E-02 |
| *cel-cel-miR-58c-3p* | 1.21 | 1.29E-04 | 1.74 | 4.05E-01 | 4.19 | 2.73E-03 | 2.15 | 2.36E-01 |
| *cel-miR-1820-5p* | -0.27 | 2.35E-01 | -0.20 | 7.56E-01 | 1.19 | 5.10E-04 | -0.13 | 8.11E-01 |
| *cel-miR-246-5p* | 0.33 | 4.37E-01 | 0.98 | 2.60E-01 | -0.32 | 7.36E-01 | 1.79 | 1.11E-02 |
| *cel-miR-51-3p* | 0.79 | 2.05E-03 | 1.19 | 1.51E-02 | -0.71 | 1.79E-01 | 0.23 | 7.60E-01 |
| *cel-miR-59-5p* | -1.85 | 5.19E-03 | -2.11 | 2.71E-01 | -3.38 | 5.47E-02 | -2.44 | 1.91E-01 |
| *cel-miR-52-3p* | -1.36 | 7.34E-06 | -1.27 | 6.97E-02 | -1.26 | 5.16E-02 | -1.06 | 1.48E-01 |
| *cel-miR-2217b-3-3p* | 1.23 | 3.12E-02 | 1.17 | 5.04E-01 | 0.25 | 8.81E-01 | 1.51 | 3.20E-01 |
| *cel-miR-78-3p* | 1.01 | 2.25E-03 | 1.30 | 5.85E-02 | 0.35 | 6.80E-01 | -0.89 | 2.36E-01 |
| *cel-miR-2217b-4-3p* | 1.23 | 3.12E-02 | 1.17 | 5.04E-01 | 0.25 | 8.81E-01 | 1.51 | 3.20E-01 |
| *cel-miR-789-2-5p* | 0.93 | 4.37E-04 | 0.74 | 3.08E-01 | 1.51 | 4.60E-03 | 1.43 | 1.34E-02 |
| *cel-miR-792-3p* | -0.08 | 8.61E-01 | -0.40 | 8.25E-01 | 1.89 | 4.60E-02 | -0.33 | 8.14E-01 |
| *cel-miR-1821-3p* | -0.73 | 5.35E-03 | -1.21 | 2.55E-02 | 0.47 | 4.34E-01 | -0.82 | 1.73E-01 |
| *cel-miR-8200-3p* | 2.46 | 2.20E-02 | 0.95 | 8.07E-01 | 1.54 | 5.64E-01 | 1.85 | 1.00E+00 |
| *cel-miR-4922-2-3p* | -1.17 | 1.79E-02 | -1.86 | 1.85E-01 | -1.32 | 3.38E-01 | -1.37 | 3.52E-01 |
| *cel-miR-4922-1-3p* | -1.17 | 1.74E-02 | -1.87 | 1.85E-01 | -1.32 | 3.38E-01 | -1.37 | 3.51E-01 |
| *cel-miR-253-3p* | 1.51 | 1.84E-08 | 1.80 | 5.03E-04 | 1.30 | 1.07E-02 | 1.08 | 5.90E-02 |
| *cel-miR-70-3p* | 0.21 | 4.22E-01 | -0.36 | 5.38E-01 | 0.29 | 5.47E-01 | 1.06 | 9.54E-03 |
| *cel-miR-70-5p* | -0.05 | 8.89E-01 | -0.02 | 9.93E-01 | -0.21 | 8.17E-01 | 2.43 | 3.52E-05 |
| *cel-miR-250-5p* | 0.40 | 1.12E-01 | 0.41 | 5.35E-01 | -1.46 | 5.10E-04 | 0.22 | 7.38E-01 |
| *cel-miR-48-3p* | -0.08 | 6.88E-01 | -0.38 | 3.25E-01 | -1.07 | 9.85E-05 | -0.72 | 2.06E-02 |
| *cel-miR-241-3p* | 0.16 | 3.99E-01 | -0.06 | 9.41E-01 | -1.36 | 9.18E-07 | -0.22 | 6.18E-01 |
| *cel-miR-796-5p* | -0.93 | 9.84E-02 | -2.23 | 8.30E-03 | 1.09 | 2.42E-01 | -1.47 | 1.27E-01 |
| *cel-miR-4807-3p* | -1.59 | 2.92E-05 | -2.67 | 4.20E-05 | -0.03 | 9.86E-01 | -0.59 | 5.00E-01 |
| *cel-miR-4808-3p* | -0.24 | 6.15E-01 | -1.71 | 7.34E-03 | -0.51 | 4.91E-01 | 0.07 | 9.42E-01 |
| *cel-miR-4809-3p* | -0.60 | 5.65E-01 | -3.02 | 3.48E-02 | -0.42 | 8.26E-01 | -1.45 | 3.81E-01 |
| *cel-miR-2220-3p* | -0.80 | 3.84E-02 | -2.40 | 5.20E-05 | -0.87 | 2.01E-01 | -1.05 | 1.36E-01 |
| *cel-miR-1018-3p* | -1.33 | 1.20E-04 | -1.96 | 2.58E-03 | 0.65 | 3.76E-01 | -0.37 | 7.01E-01 |
| *cel-miR-8196a-5p* | 0.63 | 3.40E-01 | -0.14 | 9.41E-01 | -3.02 | 1.97E-03 | -2.37 | 1.29E-02 |
| *cel-miR-73-3p* | 0.40 | 1.65E-02 | -0.46 | 2.28E-01 | 1.04 | 2.33E-04 | -0.44 | 2.36E-01 |
| *cel-miR-34-5p* | -0.49 | 1.05E-02 | -1.06 | 3.10E-03 | -0.88 | 9.69E-03 | -0.54 | 1.99E-01 |
| *cel-miR-359-3p* | 0.60 | 4.24E-02 | 0.37 | 6.62E-01 | 1.73 | 4.82E-04 | 0.26 | 7.38E-01 |
| *cel-miR-785-3p* | 0.48 | 3.97E-02 | 0.45 | 4.78E-01 | 1.49 | 1.64E-04 | 0.84 | 7.54E-02 |
| *cel-miR-249-3p* | -0.12 | 7.28E-01 | -0.04 | 9.73E-01 | 1.58 | 4.95E-04 | 0.23 | 7.66E-01 |
| *cel-miR-247-3p* | -1.04 | 4.70E-03 | -1.51 | 5.42E-02 | -1.57 | 2.66E-02 | 0.15 | 8.93E-01 |
| *cel-miR-797-5p* | -1.73 | 3.14E-07 | -2.25 | 3.19E-03 | -2.41 | 8.39E-04 | -0.61 | 5.57E-01 |
| *cel-miR-797-3p* | -1.60 | 8.18E-08 | 0.27 | 9.28E-01 | 0.53 | 7.79E-01 | 1.30 | 4.32E-01 |
| *cel-miR-230-3p* | -0.65 | 4.77E-03 | -0.73 | 1.48E-01 | -1.73 | 8.16E-06 | -1.23 | 4.91E-03 |
| *cel-miR-784-3p* | 1.06 | 1.12E-03 | 0.88 | 4.78E-01 | 1.18 | 2.38E-01 | 0.26 | 8.34E-01 |
| *cel-miR-784-5p* | 0.67 | 4.77E-05 | 0.46 | 2.63E-01 | 1.72 | 1.28E-09 | 0.20 | 6.73E-01 |
| *cel-miR-237-5p* | 0.48 | 1.19E-04 | 0.83 | 3.74E-04 | 1.43 | 8.30E-12 | 0.50 | 5.43E-02 |
| *cel-miR-788-5p* | -1.23 | 6.71E-03 | -2.36 | 6.10E-03 | -2.97 | 1.87E-04 | -1.24 | 2.33E-01 |
| *cel-miR-49-5p* | -0.04 | 8.78E-01 | -0.90 | 6.10E-03 | 1.02 | 6.96E-04 | -1.38 | 5.55E-06 |
| *cel-miR-49-3p* | 0.07 | 7.28E-01 | -0.36 | 2.63E-01 | 1.18 | 1.55E-07 | -0.50 | 6.73E-02 |
| *cel-miR-251-5p* | 0.35 | 2.77E-01 | 0.40 | 6.56E-01 | 1.50 | 5.03E-03 | -0.74 | 3.02E-01 |
| *cel-miR-254-3p* | 0.26 | 2.77E-01 | -0.24 | 7.11E-01 | 1.12 | 2.18E-03 | -0.01 | 9.86E-01 |
| *cel-miR-239b-5p* | -1.70 | 5.30E-05 | -2.62 | 1.83E-03 | -2.02 | 1.33E-02 | -0.01 | 9.86E-01 |
| *cel-miR-239a-5p* | -0.87 | 5.99E-04 | -1.38 | 4.12E-03 | -2.01 | 2.59E-06 | -0.22 | 7.64E-01 |
| *cel-miR-239a-3p* | -0.88 | 5.81E-05 | -1.53 | 2.03E-04 | -1.12 | 5.27E-03 | -0.61 | 2.36E-01 |
| *cel-miR-62-3p* | -0.36 | 8.17E-02 | -0.87 | 2.47E-02 | -1.77 | 6.06E-08 | -0.94 | 1.29E-02 |
| *cel-miR-55-5p* | -0.49 | 2.08E-02 | 0.06 | 9.48E-01 | -1.46 | 2.85E-03 | 0.08 | 9.12E-01 |
| *cel-miR-392-3p* | -2.06 | 3.83E-02 | -2.51 | 3.48E-01 | -3.01 | 2.60E-01 | -1.77 | 1.00E+00 |
| *cel-miR-5592-3p* | 0.65 | 2.39E-03 | 1.11 | 6.10E-03 | 0.48 | 2.84E-01 | 1.05 | 1.15E-02 |
| *cel-miR-5592-5p* | 0.68 | 1.90E-02 | 0.77 | 2.63E-01 | 1.22 | 1.94E-02 | 0.86 | 1.69E-01 |
| *cel-miR-8196b-3p* | 0.96 | 6.94E-05 | 1.26 | 4.88E-02 | 0.13 | 8.79E-01 | 0.68 | 3.55E-01 |
| *cel-miR-8196b-5p* | 0.33 | 6.61E-01 | -0.30 | 8.47E-01 | -2.60 | 5.03E-03 | -2.12 | 3.08E-02 |
| *cel-let-7-3p* | -1.84 | 5.62E-04 | -1.88 | 1.44E-01 | -3.64 | 5.10E-04 | -0.75 | 6.20E-01 |
| *cel-miR-1829b-5p* | 0.73 | 7.90E-03 | 1.54 | 1.48E-02 | 0.12 | 8.81E-01 | 1.39 | 3.40E-02 |
| *cel-miR-1829c-5p* | 1.17 | 1.79E-08 | 1.59 | 1.21E-04 | 0.61 | 1.99E-01 | 0.94 | 4.32E-02 |
| *cel-miR-1829a-5p* | 1.41 | 4.19E-40 | 1.44 | 4.54E-12 | 1.26 | 2.55E-09 | 0.53 | 3.96E-02 |
| *cel-miR-2212-5p* | 0.55 | 3.56E-02 | 0.96 | 1.85E-01 | 2.37 | 1.29E-05 | 1.21 | 6.53E-02 |
| *cel-miR-1819-3p* | 0.69 | 7.54E-03 | 1.68 | 1.51E-02 | 4.11 | 2.74E-12 | 2.35 | 1.95E-04 |
| *cel-miR-2217b-1-3p* | 1.23 | 3.12E-02 | 1.17 | 5.04E-01 | 0.25 | 8.81E-01 | 1.51 | 3.20E-01 |

# Table S21. The DEGs of LBD group compared to wild type (Figure 8A).

| **Genes** | **α-syn_A53T_** | ***P*-adjust** | **α-syn_A53T_;Aβ_1-42_** | ***P*-adjust** | **α-syn_A53T_;TDP-43** | ***P*-adjust** | **α-syn_A53T_;Tau_pro-agg_** | ***P*-adjust** | **α-syn_A53T_;Aβ_1-42_;Tau _pro-agg_** | ***P*-adjust** |
| --- | --- | --- | --- | --- | --- | --- | --- | --- | --- | --- |
| *gsa-1* | -0.36 | 5.13E-01 | -1.17 | 4.37E-02 | -0.37 | 5.55E-01 | -1.43 | 5.21E-03 | -1.58 | 1.76E-03 |
| *Y71F9AL.9* | -0.23 | 3.57E-01 | -1.22 | 4.43E-07 | 0.37 | 1.91E-01 | -1.29 | 3.67E-08 | -1.46 | 2.45E-10 |
| *chd-7* | 0.48 | 2.47E-01 | 0.03 | 9.82E-01 | 0.27 | 5.98E-01 | -1.21 | 3.59E-03 | -1.31 | 1.37E-03 |
| *ptr-10* | 1.29 | 1.59E-01 | 0.55 | 7.32E-01 | 1.72 | 1.30E-01 | -2.45 | 1.31E-02 | -0.92 | 3.74E-01 |
| *gld-2* | -0.07 | 7.83E-01 | -0.41 | 9.53E-02 | 0.06 | 8.34E-01 | -1.13 | 3.02E-08 | -1.65 | 2.55E-16 |
| *cox-4* | -0.71 | 2.86E-02 | -1.68 | 6.36E-06 | 0.18 | 6.99E-01 | -0.84 | 2.38E-02 | -1.03 | 4.58E-03 |
| *B0454.6* | 0.50 | 6.97E-02 | 0.11 | 8.49E-01 | 0.62 | 7.89E-02 | -1.84 | 4.31E-10 | -2.17 | 1.23E-13 |
| *F58F12.1* | -0.85 | 1.79E-02 | -1.65 | 8.80E-05 | -0.03 | 9.57E-01 | -0.56 | 2.00E-01 | -1.08 | 7.63E-03 |
| *oig-8* | -1.33 | 9.15E-03 | -1.00 | 1.56E-01 | -1.56 | 2.91E-02 | -0.54 | 4.20E-01 | 0.08 | 9.09E-01 |
| *art-1* | -0.19 | 6.06E-01 | -1.37 | 1.01E-04 | 0.47 | 2.53E-01 | -0.35 | 3.66E-01 | -0.77 | 2.57E-02 |
| *skpo-3* | -0.81 | 7.88E-04 | -1.47 | 6.31E-07 | -0.90 | 8.89E-03 | -2.51 | 3.09E-19 | -3.06 | 3.10E-28 |
| *K02A2.5* | 1.92 | 5.46E-04 | 1.31 | 9.72E-02 | 1.09 | 1.75E-01 | -0.27 | 7.85E-01 | 0.55 | 4.65E-01 |
| *srlf-1* | -0.77 | 4.03E-05 | -1.19 | 3.10E-07 | 0.20 | 4.74E-01 | -1.35 | 1.50E-09 | -1.22 | 5.14E-08 |
| *F49E12.12* | 0.46 | 5.46E-01 | 0.02 | 9.95E-01 | 1.15 | 1.64E-01 | -1.92 | 7.82E-03 | -1.43 | 4.69E-02 |
| *T24B8.7* | -0.19 | 5.51E-01 | -0.29 | 4.82E-01 | -0.10 | 7.92E-01 | -1.02 | 3.68E-04 | -1.22 | 1.37E-05 |
| *dnj-20* | -0.49 | 4.02E-09 | -1.04 | 3.81E-24 | -0.09 | 4.88E-01 | -1.32 | 3.96E-39 | -0.71 | 3.62E-12 |
| *mab-3* | -0.26 | 6.68E-01 | -0.44 | 5.78E-01 | 0.14 | 8.49E-01 | -1.58 | 6.32E-03 | -1.11 | 5.09E-02 |
| *clh-1* | -1.20 | 4.84E-05 | -1.38 | 3.12E-04 | -1.30 | 2.05E-03 | -0.83 | 2.58E-02 | -1.44 | 5.48E-05 |
| *men-1* | -0.64 | 1.23E-03 | -1.20 | 5.43E-07 | -0.15 | 6.08E-01 | -0.50 | 4.06E-02 | -0.42 | 8.58E-02 |
| *K10H10.4* | 0.86 | 4.95E-02 | 0.95 | 9.37E-02 | 0.67 | 2.48E-01 | -1.92 | 1.10E-04 | -2.49 | 5.56E-07 |
| *Y53F4B.27* | 1.02 | 2.72E-01 | 0.05 | 9.87E-01 | 1.31 | 2.40E-01 | -3.23 | 5.62E-04 | -3.01 | 1.16E-03 |
| *H10E21.5* | 1.12 | 2.15E-03 | 0.36 | 5.88E-01 | 1.06 | 3.85E-02 | -1.73 | 9.57E-05 | -1.89 | 1.65E-05 |
| *K02F3.2* | -0.64 | 2.27E-02 | -1.05 | 2.05E-03 | -0.28 | 4.75E-01 | -0.63 | 5.34E-02 | -0.59 | 7.01E-02 |
| *pitp-1* | -1.08 | 4.13E-04 | -1.66 | 1.18E-05 | -0.72 | 1.02E-01 | -1.06 | 4.48E-03 | -1.38 | 1.56E-04 |
| *larp-1* | -0.29 | 1.32E-01 | -1.12 | 3.48E-08 | 0.00 | 9.95E-01 | -1.71 | 1.10E-18 | -1.91 | 2.87E-23 |
| *atp-2* | -0.60 | 6.76E-02 | -1.65 | 5.63E-06 | 0.22 | 6.25E-01 | -0.85 | 2.00E-02 | -1.10 | 1.78E-03 |
| *oatr-1* | -0.35 | 1.12E-01 | -1.11 | 3.33E-06 | 0.28 | 3.17E-01 | -0.54 | 2.52E-02 | -0.85 | 2.13E-04 |
| *nas-4* | -0.54 | 2.17E-01 | -0.92 | 8.43E-02 | -0.87 | 1.09E-01 | -2.23 | 2.64E-06 | -2.12 | 6.62E-06 |
| *R05H11.2* | 2.78 | 2.05E-05 | 1.06 | 3.26E-01 | 2.68 | 4.39E-03 | -0.59 | 5.69E-01 | -0.76 | 4.22E-01 |
| *mfsd-6* | 0.47 | 9.82E-02 | 0.34 | 4.28E-01 | 0.40 | 2.84E-01 | -1.69 | 1.51E-07 | -1.03 | 1.16E-03 |
| *tyr-2* | 0.54 | 5.82E-02 | 0.15 | 7.93E-01 | 0.55 | 1.38E-01 | -2.18 | 9.09E-13 | -1.61 | 1.69E-07 |
| *zag-1* | 1.74 | 3.19E-05 | 0.77 | 2.48E-01 | 1.51 | 1.31E-02 | -1.49 | 6.35E-03 | -1.79 | 9.71E-04 |
| *ell-1* | -0.46 | 1.04E-01 | -1.10 | 5.63E-04 | -0.17 | 6.70E-01 | -0.32 | 3.49E-01 | -0.94 | 1.79E-03 |
| *ZK180.5* | 0.95 | 2.46E-01 | 0.11 | 9.55E-01 | 0.96 | 3.34E-01 | -3.25 | 6.96E-05 | -4.49 | 1.87E-08 |
| *ogdh-1* | -0.48 | 4.51E-02 | -1.25 | 2.85E-06 | 0.06 | 8.70E-01 | -0.10 | 7.69E-01 | -0.92 | 3.64E-04 |
| *ima-3* | -0.20 | 3.88E-01 | -1.03 | 3.96E-06 | 0.26 | 3.20E-01 | -0.44 | 5.19E-02 | -1.49 | 1.48E-12 |
| *gtbp-1* | -0.16 | 5.23E-01 | -0.70 | 4.15E-03 | -0.07 | 8.19E-01 | -1.06 | 2.17E-06 | -1.50 | 6.40E-12 |
| *F01G4.6* | -0.52 | 5.79E-02 | -1.14 | 2.96E-04 | 0.07 | 8.70E-01 | -0.36 | 2.73E-01 | -0.80 | 7.67E-03 |
| *lips-5* | 1.29 | 2.56E-04 | 0.45 | 4.68E-01 | 0.93 | 6.96E-02 | -1.05 | 2.11E-02 | 0.19 | 7.01E-01 |
| *H01G02.3* | 0.60 | 4.74E-02 | 0.15 | 8.09E-01 | 0.80 | 4.52E-02 | -1.11 | 1.42E-03 | -0.53 | 1.36E-01 |
| *unc-30* | 1.16 | 3.87E-03 | 1.00 | 6.51E-02 | 1.40 | 1.11E-02 | -0.40 | 5.13E-01 | 0.56 | 2.85E-01 |
| *R05A10.6* | 1.16 | 7.42E-02 | 0.14 | 9.31E-01 | 1.33 | 1.13E-01 | -3.79 | 3.04E-05 | -5.01 | 9.69E-06 |
| *R02F11.3* | -0.56 | 2.15E-04 | -0.98 | 1.76E-07 | -0.31 | 1.63E-01 | -1.10 | 4.56E-09 | -1.02 | 4.26E-08 |
| *K11G9.5* | -1.19 | 4.48E-05 | -0.30 | 5.82E-01 | -1.37 | 1.02E-03 | -0.91 | 1.34E-02 | -1.18 | 1.04E-03 |
| *T15B7.1* | -0.86 | 1.05E-02 | -1.88 | 1.71E-06 | 0.38 | 4.26E-01 | -2.05 | 6.19E-08 | -0.73 | 6.60E-02 |
| *unc-70* | -0.16 | 6.95E-01 | -0.95 | 1.34E-02 | 0.05 | 9.27E-01 | -1.52 | 9.93E-06 | -1.69 | 6.34E-07 |
| *C05C8.7* | -0.31 | 4.62E-01 | -0.97 | 3.18E-02 | 0.11 | 8.40E-01 | -1.97 | 5.46E-07 | -0.98 | 1.47E-02 |
| *rpm-1* | 0.31 | 4.34E-01 | 0.28 | 6.35E-01 | -0.28 | 5.51E-01 | -1.53 | 6.11E-05 | -0.81 | 3.56E-02 |
| *ZK287.1* | 0.73 | 7.68E-02 | 0.42 | 5.27E-01 | 0.75 | 1.58E-01 | -1.26 | 7.60E-03 | -0.08 | 8.89E-01 |
| *F32D8.3* | 0.34 | 3.98E-01 | 0.09 | 9.15E-01 | 0.83 | 7.08E-02 | -1.10 | 7.61E-03 | -0.82 | 4.63E-02 |
| *dop-5* | 0.97 | 3.60E-02 | 0.54 | 4.62E-01 | 1.09 | 7.69E-02 | -1.19 | 3.23E-02 | -1.46 | 8.18E-03 |
| *sipa-1* | 0.55 | 3.75E-02 | 0.02 | 9.77E-01 | 0.56 | 1.11E-01 | -1.39 | 2.23E-06 | -0.89 | 2.58E-03 |
| *shw-3* | 0.49 | 1.45E-01 | 0.02 | 9.91E-01 | 0.92 | 2.47E-02 | -1.15 | 2.58E-03 | -0.77 | 4.17E-02 |
| *gar-3* | 0.88 | 1.86E-02 | 0.49 | 4.08E-01 | 0.87 | 8.71E-02 | -2.26 | 6.01E-07 | -0.93 | 3.65E-02 |
| *W06A7.4* | -1.80 | 1.72E-06 | -1.39 | 6.78E-03 | -1.71 | 1.70E-03 | -1.11 | 2.21E-02 | -1.36 | 3.89E-03 |
| *grsp-1* | -0.16 | 7.49E-01 | -0.66 | 2.11E-01 | 0.49 | 3.35E-01 | -2.46 | 2.64E-09 | -2.80 | 9.63E-12 |
| *K02E10.7* | -0.03 | 9.56E-01 | -0.81 | 4.26E-02 | 0.19 | 6.68E-01 | -1.59 | 1.85E-05 | -0.95 | 8.95E-03 |
| *B0410.3* | -0.93 | 3.74E-04 | -1.05 | 1.97E-03 | -0.96 | 1.04E-02 | -1.04 | 1.15E-03 | 0.24 | 4.93E-01 |
| *gbb-1* | 0.65 | 8.80E-02 | 0.08 | 9.30E-01 | 0.59 | 2.25E-01 | -1.65 | 9.76E-05 | -0.64 | 1.42E-01 |
| *marg-1* | -0.20 | 7.57E-01 | -1.43 | 1.95E-02 | 0.15 | 8.39E-01 | -0.63 | 2.96E-01 | -1.40 | 1.30E-02 |
| *pdi-2* | -0.27 | 5.89E-01 | -1.39 | 4.04E-03 | 0.62 | 2.50E-01 | -1.45 | 1.28E-03 | -1.68 | 1.31E-04 |
| *F46C8.8* | 0.95 | 2.05E-01 | 0.14 | 9.39E-01 | 1.29 | 1.60E-01 | -2.24 | 4.09E-03 | -3.99 | 2.32E-07 |
| *tbc-18* | 0.41 | 1.77E-01 | 0.11 | 8.60E-01 | 0.35 | 3.64E-01 | -1.31 | 7.32E-05 | -0.21 | 5.56E-01 |
| *suca-1* | -0.38 | 2.08E-01 | -1.38 | 1.34E-05 | 0.27 | 4.70E-01 | -0.97 | 1.67E-03 | -1.41 | 3.10E-06 |
| *ldb-1* | 0.63 | 1.28E-01 | 0.04 | 9.74E-01 | 0.74 | 1.51E-01 | -1.35 | 1.99E-03 | -1.29 | 3.01E-03 |
| *igeg-2* | 1.08 | 2.55E-05 | 0.44 | 2.86E-01 | 0.81 | 3.14E-02 | -0.31 | 4.10E-01 | 0.30 | 4.02E-01 |
| *F48C5.2* | 0.65 | 8.76E-02 | 0.50 | 3.60E-01 | 0.87 | 7.28E-02 | -1.30 | 2.10E-03 | -0.94 | 2.52E-02 |
| *frm-3* | 0.58 | 2.88E-01 | 0.02 | 9.94E-01 | 0.70 | 2.79E-01 | -2.57 | 3.01E-06 | -2.12 | 9.21E-05 |
| *F57G12.1* | -0.32 | 1.11E-01 | -0.59 | 1.03E-02 | -0.14 | 5.92E-01 | -1.15 | 3.54E-08 | -0.98 | 2.22E-06 |
| *nhr-25* | 1.37 | 3.42E-02 | 0.67 | 5.30E-01 | 1.55 | 7.14E-02 | -0.67 | 4.21E-01 | 0.27 | 7.47E-01 |
| *pag-3* | 1.30 | 4.61E-03 | 0.50 | 5.27E-01 | 1.42 | 2.49E-02 | -2.33 | 6.51E-04 | -1.10 | 7.45E-02 |
| *seb-3* | 0.64 | 2.41E-02 | 0.44 | 2.90E-01 | 0.50 | 1.85E-01 | -1.17 | 6.68E-04 | -0.59 | 8.57E-02 |
| *nlp-67* | 1.23 | 1.37E-02 | 0.25 | 8.10E-01 | 1.29 | 5.88E-02 | -1.83 | 2.74E-03 | -1.88 | 1.93E-03 |
| *K08B5.1* | 0.79 | 1.67E-02 | 0.06 | 9.45E-01 | 0.31 | 5.16E-01 | -1.07 | 8.40E-03 | -1.91 | 4.11E-06 |
| *cdh-9* | 0.61 | 1.25E-01 | 0.03 | 9.85E-01 | 0.87 | 7.90E-02 | -2.30 | 6.92E-07 | -1.93 | 1.81E-05 |
| *mct-6* | -0.82 | 1.95E-12 | -1.61 | 4.08E-28 | -0.21 | 2.60E-01 | -1.92 | 7.01E-40 | -2.32 | 7.06E-57 |
| *mbl-1* | -0.16 | 3.92E-01 | -0.81 | 1.04E-05 | 0.24 | 2.64E-01 | -1.32 | 3.36E-14 | -1.43 | 1.59E-16 |
| *ptrn-1* | -0.09 | 8.10E-01 | -0.85 | 1.74E-02 | 0.14 | 7.38E-01 | -1.54 | 1.34E-06 | -1.51 | 1.94E-06 |

# Table S22. The DEGs of WT, α-syn_A53T_;TDP-43, α-syn_A53T_;Tau_pro-agg_, and α-syn_A53T_;Aβ_1-42_;Tau_pro-agg_ compared with α-syn_A53T_ (Figure 8D).

| **Gene** | **WT** | ***P*-adjust** | **α-syn_A53T_;TDP-43** | ***P*-adjust** | **α-syn_A53T_;Tau_pro-agg_** | ***P*-adjust** | **α-syn_A53T_;Aβ_1-42_;Tau_pro-agg_** | ***P*-adjust** |
| --- | --- | --- | --- | --- | --- | --- | --- | --- |
| *C30F12.2* | 0.63 | 2.10E-03 | 0.16 | 8.19E-01 | 1.30 | 6.74E-07 | 1.46 | 2.19E-08 |
| *T23G11.7* | 0.04 | 8.79E-01 | 0.16 | 7.54E-01 | 1.12 | 6.15E-08 | 0.53 | 1.44E-02 |
| *cdc-48.3* | 0.30 | 3.21E-02 | 0.00 | 9.95E-01 | 1.26 | 1.06E-14 | 0.60 | 3.95E-04 |
| *T23B7.3* | 0.43 | 2.38E-01 | 0.60 | 4.02E-01 | 0.92 | 2.43E-02 | 1.04 | 1.05E-02 |
| *F46C5.10* | 0.62 | 2.37E-02 | 0.01 | 9.94E-01 | 0.51 | 1.64E-01 | 1.36 | 6.58E-05 |
| *T23G7.2* | 0.13 | 5.97E-01 | 0.02 | 9.80E-01 | 0.77 | 2.15E-03 | 1.67 | 5.31E-12 |
| *ZK809.8* | 1.21 | 6.65E-10 | 0.85 | 3.16E-02 | 1.11 | 6.44E-05 | 1.65 | 1.26E-09 |
| *C39E9.8* | 0.54 | 3.11E-03 | 0.11 | 8.62E-01 | 0.04 | 9.17E-01 | 1.50 | 5.84E-11 |
| *grl-8* | 1.11 | 2.45E-03 | 0.32 | 7.90E-01 | 1.03 | 3.44E-02 | 0.77 | 1.28E-01 |
| *ugt-38* | 1.14 | 5.08E-03 | 1.31 | 8.91E-02 | 1.10 | 3.96E-02 | 1.42 | 6.40E-03 |
| *lbp-3* | 0.83 | 2.90E-07 | 0.45 | 2.30E-01 | 0.73 | 1.41E-03 | 1.65 | 5.15E-14 |
| *cal-5* | 1.01 | 1.07E-05 | 0.23 | 7.54E-01 | 0.57 | 8.69E-02 | 0.24 | 5.06E-01 |

# Table S23. The mitochondrial morphology observation of in *Caenorhabditis elegans* (Figure 9K, day 1 at 23℃).

| **Tukey's multiple comparisons test** | **Mean Diff.** | **95% CI of diff.** | **Summary** | **Adjusted *P* Value** |
| --- | --- | --- | --- | --- |
| WT vs. Aβ_1-42_ | -0.08461 | -0.4827 to 0.3135 | ns | 0.9996 |
| WT vs. TDP-43 | -0.07507 | -0.4547 to 0.3046 | ns | 0.9998 |
| WT vs. Tau_pro-agg_ | -0.6631 | -1.051 to -0.2750 | **** | <0.0001 |
| WT vs. α-syn_A53T_ | -0.9113 | -1.322 to -0.5002 | **** | <0.0001 |
| WT vs. α-syn_A53T_;Aβ_1-42_ | -1.602 | -1.994 to -1.209 | **** | <0.0001 |
| WT vs. α-syn_A53T_;TDP-43 | -0.2903 | -0.7004 to 0.1199 | ns | 0.4281 |
| WT vs. Aβ_1-42_;Tau_pro-agg_ | -0.1801 | -0.5435 to 0.1833 | ns | 0.8626 |
| WT vs. α-syn_A53T_;Tau_pro-agg_ | -0.4422 | -0.8164 to -0.06799 | ** | 0.0072 |
| WT vs. α-syn_A53T_;Aβ_1-42_;Tau_pro-agg_ | -0.3083 | -0.6849 to 0.06830 | ns | 0.2213 |
| Aβ_1-42_ vs. α-syn_A53T_;Aβ_1-42_ | -1.517 | -1.918 to -1.117 | **** | <0.0001 |
| Aβ_1-42_ vs. Aβ_1-42_;Tau_pro-agg_ | -0.09548 | -0.4676 to 0.2767 | ns | 0.9984 |
| Aβ_1-42_ vs. α-syn_A53T_;Aβ_1-42_;Tau_pro-agg_ | -0.2237 | -0.6087 to 0.1613 | ns | 0.7091 |
| TDP-43 vs. α-syn_A53T_;TDP-43 | -0.2152 | -0.6156 to 0.1851 | ns | 0.7939 |
| Tau_pro-agg_ vs. Aβ_1-42_;Tau_pro-agg_ | 0.4831 | 0.1217 to 0.8445 | ** | 0.001 |
| Tau_pro-agg_ vs. α-syn_A53T_;Tau_pro-agg_ | 0.221 | -0.1513 to 0.5932 | ns | 0.6824 |
| Tau_pro-agg_ vs. α-syn_A53T_;Aβ_1-42_;Tau_pro-agg_ | 0.3548 | -0.01981 to 0.7295 | ns | 0.0811 |
| α-syn_A53T_ vs. α-syn_A53T_;Aβ_1-42_ | -0.6906 | -1.104 to -0.2771 | **** | <0.0001 |
| α-syn_A53T_ vs. α-syn_A53T_;TDP-43 | 0.621 | 0.1907 to 1.051 | *** | 0.0002 |
| α-syn_A53T_ vs. α-syn_A53T_;Tau_pro-agg_ | 0.4691 | 0.07297 to 0.8652 | ** | 0.007 |
| α-syn_A53T_ vs. α-syn_A53T_;Aβ_1-42_;Tau_pro-agg_ | 0.603 | 0.2046 to 1.001 | **** | <0.0001 |
| α-syn_A53T_;Aβ_1-42_ vs. α-syn_A53T_;Aβ_1-42_;Tau_pro-agg_ | 1.294 | 0.9144 to 1.673 | **** | <0.0001 |
| Aβ_1-42_;Tau_pro-agg_ vs. α-syn_A53T_;Aβ_1-42_;Tau_pro-agg_ | -0.1282 | -0.4772 to 0.2208 | ns | 0.9775 |
| α-syn_A53T_;Tau_pro-agg_ vs. α-syn_A53T_;Aβ_1-42_;Tau_pro-agg_ | 0.1339 | -0.2263 to 0.4941 | ns | 0.9757 |

# **References**

1. Wang, C., Saar, V., Leung, K.L., Chen, L., and Wong, G. (2018). Human amyloid beta peptide and tau co-expression impairs behavior and causes specific gene expression changes in Caenorhabditis elegans. Neurobiol Dis *109*, 88-101. 10.1016/j.nbd.2017.10.003.

2. Shen, L., Wang, C., Chen, L., Leung, K.L., Lo, E., Lakso, M., and Wong, G. (2020). TDP-1/TDP-43 potentiates human alpha-Synuclein (HASN) neurodegeneration in Caenorhabditis elegans. Biochim Biophys Acta Mol Basis Dis *1866*, 165876. 10.1016/j.bbadis.2020.165876.

3. Huang, X., Wang, C., Chen, L., Zhang, T., Leung, K.L., and Wong, G. (2021). Human amyloid beta and alpha-synuclein co-expression in neurons impair behavior and recapitulate features for Lewy body dementia in Caenorhabditis elegans. Biochim Biophys Acta Mol Basis Dis *1867*, 166203. 10.1016/j.bbadis.2021.166203.
